# A scoping review on the roles and tasks of peer reviewers in the manuscript review process in biomedical journals

**DOI:** 10.1186/s12916-019-1347-0

**Published:** 2019-06-20

**Authors:** Ketevan Glonti, Daniel Cauchi, Erik Cobo, Isabelle Boutron, David Moher, Darko Hren

**Affiliations:** 1 Department of Psychology, University of Split, Faculty of Humanities and Social Sciences, Split, Croatia; 2 INSERM, U1153 Epidemiology and Biostatistics Sorbonne Paris Cité Research Center (CRESS), Methods of Therapeutic Evaluation Of Chronic Diseases Team (METHODS), F-75014 Paris, France; 3 Paris Descartes University, Sorbonne Paris Cité, Paris, France; 4 Department of Public Health, Faculty of Medicine and Surgery, University of Malta, Msida, Malta; 5 Statistics and Operations Research Department, Barcelona-Tech, UPC, c/ Jordi Girona 1, C5-213, 08034 Barcelona, Spain; 6 Center for Journalology, Clinical Epidemiology Program, Ottawa Hospital Research Institute, Ottawa, Canada

**Keywords:** Biomedical, Roles, Tasks, Competencies, Journal, Peer reviewer, Scoping review

## Abstract

**Background:**

Although peer reviewers play a key role in the manuscript review process, their roles and tasks are poorly defined. Clarity around this issue is important as it may influence the quality of peer reviewer reports. This scoping review explored the roles and tasks of peer reviewers of biomedical journals.

**Methods:**

Comprehensive literature searches were conducted in Cochrane Library, Cumulative Index to Nursing and Allied Health Literature, Educational Resources Information Center, EMBASE, MEDLINE, PsycINFO, Scopus and Web of Science from inception up to May 2017. There were no date and language restrictions. We also searched for grey literature. Studies with statements mentioning roles, tasks and competencies pertaining to the role of peer reviewers in biomedical journals were eligible for inclusion. Two reviewers independently performed study screening and selection. Relevant statements were extracted, collated and classified into themes.

**Results:**

After screening 2763 citations and 600 full-text papers, 209 articles and 13 grey literature sources were included. A total of 1426 statements related to roles were extracted, resulting in 76 unique statements. These were grouped into 13 emergent themes: proficient experts in their field (3 items), dutiful/altruistic towards scientific community (7 items), familiar with journal (2 items), unbiased and ethical professionals (18 items), self-critical professionals (4 items), reliable professionals (7 items), skilled critics (15 items), respectful communicators (6 items), gatekeepers (2 items), educators (2 items), advocates for author/editor/reader (3 items) and advisors to editors (2 items). Roles that do not fall within the remit of peer reviewers were also identified (5 items).

We also extracted 2026 statements related to peer reviewers’ tasks, resulting in 73 unique statements. These were grouped under six themes: organisation and approach to reviewing (10 items), make general comments (10 items), assess and address content for each section of the manuscript (36 items), address ethical aspects (5 items), assess manuscript presentation (8 items) and provide recommendations (4 items).

**Conclusions:**

Peer reviewers are expected to perform a large number of roles and tasks for biomedical journals. These warrant further discussion and clarification in order not to overburden these key actors.

**Electronic supplementary material:**

The online version of this article (10.1186/s12916-019-1347-0) contains supplementary material, which is available to authorized users.

## Background

Evidence indicates that there is a need to improve the quality of peer reviewer reports in biomedical journals [1, 2]. Published biomedical papers may have a direct impact on clinical practice and inform policy. Therefore, it is crucial that peer reviewer reports, a screen before the diffusion of new knowledge, are of the highest quality possible to inform editors’ decision on the fate of the manuscript [3, 4].

Unlike other professional groups, many editors and peer reviewers of biomedical journals operate largely without formal training. It is assumed that having expertise as an author provides, by default, the skills necessary to be a scientific editor and/or peer reviewer. However, this assumption is problematic, potentially having a number of negative implications for the overall quality of biomedical publishing [5].

Alongside the lack of standardised training, the lack of a clear, accepted definition of the roles and tasks of peer reviewers has also been highlighted [6]. A systematic review evaluated the impact of interventions aimed at improving the quality of peer review of randomised controlled trials (RCTs) for biomedical publications. The authors concluded that clarification of the roles and tasks of peer reviewers would be a step forward in quality improvement of peer reviewing [2]. In fact, a recent study showed that the most important tasks in peer review, as perceived by peer reviewers evaluating RCTs, were not congruent with the tasks most often requested by journal editors in their guidelines to reviewers [6].

Organisations such as the Council of Science Editors provide a general overview of reviewer roles and responsibilities [7]. However, within the biomedical field, the roles and tasks of peer reviewers are often closely related to the structural characteristics of the editorial process itself. For example, some (but not all) journals require peer reviewers to assess novelty and/or clinical relevance of articles in addition to assessing scientific rigour. Journal expectations of how a reviewer report should be written may vary. Some journals encourage reviewers to follow a specific structure in their reporting, whereas other journals prefer free text. Additionally, there may be differences between journals’ requests for peer reviewer recommendations regarding whether an article should be accepted for publication or not. Furthermore, differences in roles and tasks between journals may also be linked to the organisational set-up and resources of the journals and publishers. Given these differences, we believe that it is important to distil the core roles and tasks to enable peer reviewers to meet basic, global standards. In order to do this, we first need to compile a comprehensive list of the different roles and tasks described in the literature.

While core competencies for biomedical journal editors have already been systematically identified [8] and agreed upon [9], we are unaware of any body of literature looking into peer reviewers’ roles and tasks.

The aim of this scoping review is to determine the roles and tasks of peer reviewers as depicted in biomedical literature. For the purposes of this research, we consider ‘roles’ to refer to the overarching nature of peer reviewers’ function, whereas ‘tasks’ refer more specifically to actions that fulfil these roles.

Our specific objectives were to answer the following two research questions while summarising the existing literature:

1. What are the expected roles of peer reviewers in the editorial peer review process in biomedical journals?
2. What are the range of tasks that peer reviewers are expected to perform for biomedical journals?

## Methods

This scoping review was guided by the methodological framework proposed by Arksey and O’Malley [10], as well as the amendments made to this framework by Levac et al. [11] and by the Joanna Briggs Institute [12]. The framework consists of six consecutive stages: (1) identifying the research question, (2) identifying relevant studies; (3) study selection; (4) charting the data; (5) collating, summarising and reporting results; and (6) consultation. We performed the last stage through qualitative interviews, with results to be reported separately [13]. A study protocol containing all methodological details was published before conducting this scoping review [14]. Although initially specified in the protocol, we did not carry out the review of journal guidelines to peer reviewers. Due to the extensive volume of the initially proposed work, this aspect of the research will be carried out and published separately.

We used the PRISMA-ScR (Preferred Reporting Items for Systematic reviews and Meta-Analyses extension for Scoping Reviews) checklist to report our results (Additional file 6) [15].

### Study selection: inclusion and exclusion criteria

Any article with a specific focus and/or statements mentioning roles, tasks and competencies pertaining to the contribution of peer reviewers to the journal editorial process was included. Articles referring solely to roles and tasks that were not related to manuscript peer reviewing in biomedical journals (e.g. grant peer review, professional performance review and peer review of teaching) were excluded. There were no date and language restrictions.

### Disciplines

We adopted MEDLINE’s journal selection criteria for our definition of health. This definition includes journals that are ‘predominantly devoted to reporting original investigations in the biomedical and health sciences, including research in the basic sciences; clinical trials of therapeutic agents; effectiveness of diagnostic or therapeutic techniques; or studies relating to the behavioural, epidemiological, or educational aspects of medicine’. In order to ensure feasibility of the study, we did not include journals from the disciplines of psychology, education, physical or natural sciences.

### Study designs

The review considered all study designs to be eligible. Based on findings from a preceding scoping review of competencies for scientific editors of biomedical journals [8], it was anticipated that a substantial proportion of relevant statements would be identified in publications that are not only presenting the results of research (subsequently termed ‘research-based publications’) but also in non-research-based publications including book chapters, commentaries and editorials, as well as grey literature. Therefore, we also searched for non-peer-reviewed resources on websites.

### Search strategy for peer-reviewed literature

The Peer Review of Electronic Search Strategies (PRESS) 2015 Guideline statement was used to guide the electronic literature search strategies [16]. These were further refined in collaboration with a Health Sciences Librarian. Subsequently, the following databases were searched: Cochrane Library, Cumulative Index to Nursing and Allied Health Literature (CINAHL), Educational Resources Information Centre (ERIC), EMBASE (via Ovid), PsycINFO (via Ovid), MEDLINE (via Ovid), Scopus and Web of Science. There were no date or language restrictions. The search strategy for MEDLINE can be found in the online Additional file 1. In addition, we hand-searched websites of JAMA, Nature and Science using keywords related to peer review to identify any additional literature that was not detected by the search strategy.

### Grey literature search

We searched for grey literature on websites of existing networks (e.g. EQUATOR Network, New Frontiers of Peer Review (PEERE)), biomedical journal publishers (e.g. BMJ Publishing Group, Elsevier, Springer Nature, Taylor & Francis, Wiley) and organisations that offer resources for reviewers (including educational courses, for example those provided by Cochrane and Publons). Relevant blogs, newsletters (e.g. The METRICS Research Digest), surveys and reports of authors/reviewer workshops were also searched. We further hand-searched available abstracts from the various International Congresses on Peer Review and Scientific Publication [17, 18].

### Screening

Following the execution of the search strategy, the identified records (titles and abstracts) were collated in a reference manager (Endnote) for de-duplication. The final unique set of records was imported into a systematic review paper manager (Covidence) that facilitated independent screening by two reviewers. The screening of titles and abstracts and subsequent full-text screening was performed independently by two reviewers (KG and DC). Disagreements between reviewers were resolved by consensus.

### Charting the data

A data extraction form was developed a priori to capture information on each eligible document included in the review. General study characteristics extracted were as follows: first author name, year of publication, country of first author, language of publication and study design. For grey literature, we extracted the URL, title of the document, language of publication and who produced the document.

In addition, for all documents, we collected descriptions of any statements potentially relating to the roles and tasks of peer reviewers. Two people (KG, DC) carried out the data extraction. In the first step, data were extracted from eligible full texts into Microsoft Excel by KG. Subsequently, DC compared the full text of each eligible document with the extracted data on Microsoft Excel to ensure that all relevant information had been included.

### Collating, summarising and reporting the results

Initially, all relevant statements (full sentences) related to roles from all data sources were extracted into a Microsoft Excel sheet by KG. Subsequently, each sentence was coded into smaller text units semantically as close as possible to the original, full sentence. Overlapping or duplicate text units were collated following discussion and agreement with DC, resulting in a list of unique statements for roles. Finally, we grouped these statements into emergent overarching themes to provide a better overview of results. All relevant statements (full sentences) related to tasks from all data sources were also extracted into a Microsoft Excel sheet by KG and mapped using pre-defined categories adapted from work carried out by Hirst and Altman [19]. In order to produce a meaningful list, we only included tasks that would apply to all types of studies. Tasks that are not common to all types of studies, for example, those related specifically to RCTs and systematic reviews, were not extracted (Additional files 7 and 8).

## Results

### Literature search

**Fig. 1:**
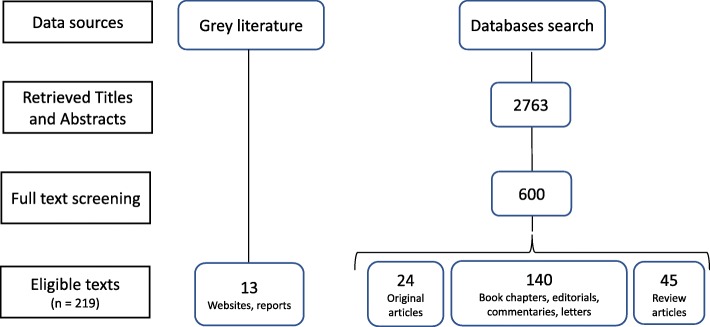
Study flow diagram

A total of 23,176 records were returned by the search strategy which included a substantial number of records related to ‘hospital peer review’. In the first step, one researcher (KG) screened all irrelevant records out by title and abstract, leaving 2763 possibly relevant articles which were then screened by two reviewers by title and abstract (KG and DC). Six hundred records were eligible for full-text screening. Disagreements regarding eligibility were resolved through discussion and achieving consensus between the two reviewers. Subsequently, 391 biomedical publications were excluded, leaving 209 publications that met the inclusion criteria (Fig. 1). From these 209 publications, there were 24 original research articles, 45 review articles and 140 book chapters, editorials, commentaries, letters and tutorials. We also included 13 grey literature sources.

### Research-based publications

A total of 24 publications from the database search were considered relevant to the roles and tasks of peer reviewers in biomedical journals (Additional file 2).

Only one of these articles was primarily focused on roles and tasks. Seven studies reported on surveys, eight were descriptive studies and two were randomised controlled trials (RCT). The remaining five articles comprised a randomised trial, two intervention studies and two systematic reviews. Publication dates ranged from 1991 to 2016. Most of the studies were published in 2013 (*n* = 4) and 2005 (*n* = 3).

All articles were written in English. Eleven of the studies’ first authors were based in the USA, three in the UK, two in France and one each in Australia, Canada, Hong Kong, India, Italy and Japan. The remaining two studies did not include details on the first authors’ country affiliation.

### Non-research-based publications

A total of 185 non-research-based publications were considered relevant.

Overall, 45 publications were review articles (Additional file 3). For the review articles, the date of publication ranged from 1974 to 2016; 2008 (*n* = 6), 2014 (*n* = 5) and 2015 (*n* = 5) were the three years with the most studies. Two review articles were in Spanish and 41 in English. This sample included 23 studies with first authors originating from the USA, five from the UK, four from Australia and Spain and one from Canada, Greece, India, Japan, Korea and Palestine.

The remaining 140 publications consisted of 122 editorials, two book chapters, 12 commentaries, two letters and two tutorials (Additional file 4). The publication dates ranged from 1983 to 2017; 2016 (*n* = 15) and 2014 (*n* = 15) were the two years with the most studies. All articles were written in English except for two articles that were written in Portuguese. This sample included 48 studies with first authors based in the USA; 10 in the UK; nine in Canada; seven in Australia; four in Brazil; three in Germany; two in Denmark, India, Portugal and Spain; and one each in Austria, Croatia, The Netherlands and Singapore.

### Search of networks and publishers

The search of networks and publishers resulted in 13 additional documents from websites. Among the sample, four documents were blogs/column, five were training/webinar documents and two were guidelines from professional associations, societies and organisations. Lastly, two were guidance documents by publishers (Additional file 5).

### Collating and summarising the data

In an effort to create a useful summary of the data, we collated and combined the statements retrieved from all included sources in Table 1 and Table 2, where we present a detailed breakdown of all themes and related statements. Each statement is linked back to the specific papers in the Additional files 2, 3, 4 and 5.

**Table 1:** Role-related statements (‘roles’ refer to the overarching nature of peer reviewers’ function. The statements are ranked by numerical frequency. Each statement is linked back to the specific papers in the Additional files 2, 3, 4 and 5)

	Item^a^		#^b^
Peer reviewers should be…			
Proficient experts in their field	1	Be expert in the subject area/matter/field and/or be familiar with/trained in research methods and statistics	70
	2	Be actively involved in research and have experience of conducting research and publishing scientific papers	15
	3	Be familiar with reporting guidelines	5
Dutiful/altruistic towards the scientific community	4	Consider peer reviewing to be a responsibility, duty and obligation to the field and to the scientific community	26
	5	Consider the act of peer reviewing as an honour and a privilege	8
	6	Indicate willingness to re-review the manuscript	7
	7	Be aware of one’s role, responsibilities and rights as a peer reviewer	4
	8	Perform reviewing task altruistically/gratis	2
	9	End one’s appointment as reviewer to create opportunity for others	1
	10	Act regularly as peer reviewer	1
Familiar with journal	11	Be familiar with journal’s mission, review process, review criteria, guidelines (i.e. both author and reviewer guidelines) and forms prior to starting the review	39
	12	Guide the substance and direction of a journal	1
Unbiased and ethical professionals	13	Declare/avoid potential or actual conflict of interest	66
	14	Maintain confidentiality of the manuscript, avoiding disclosure/discussion with others	52
	15	Be fair: evaluate manuscript in a fair manner	39
	16	Be objective: objectively judge all aspects of the manuscript	36
	17	Be unbiased in their assessment: peer reviewers should have an unbiased attitude towards an author’s gender, previous work, institution and nationality	32
	18	Review ethically: they should not use the obtained information in any way	17
	19	Be honest/frank	13
	20	Maintain integrity of the peer review process and not communicate with authors during the review process	12
	21	Inform editor if a colleague will help or has helped with review	11
	22	Review ethically: they should not copy and plagiarise	6
	23	Be aware of their own biases: peer reviewers should recognise their potential biases and hold them in check	6
	24	Upon completing the review, manuscript, illustrations and tables should be destroyed	5
	25	Review ethically: in general terms, peer reviewers are expected to undertake task in an ethical and diligent manner	4
	26	Be familiar with fundamental issues of publication integrity	4
	27	Decline review request if these cannot be performed in an unbiased manner	4
	28	Review ethically: they should not ask for their own articles to be cited	4
	29	Review ethically: they should not delay publications purposefully	4
	30	Be transparent and perform review in a transparent manner	2
Self-critical professionals	31	Prior to accepting review request, determine whether the manuscript is within one’s area of expertise (only review manuscripts in one’s own field of expertise)	35
	32	Be aware of own limitations: recognise and communicate them to the editors. If needed, recommend review by an expert (e.g. statistician)	22
	33	Be innovative and open to new ideas	13
	34	Peer reviewers should consider reviewing as a learning exercise and evaluate one’s own performance as a reviewer, i.e. read other peer reviewers’ reviews and thereby improve their own understanding of the topic and/or decision reached	8
Reliable professionals	35	Timeliness: meet journal deadline	81
	36	Consider one’s time availability prior to accepting review request	36
	37	Be willing to devote sufficient time and attention to the review task	23
	38	Respond to review requests in a timely manner	21
	39	Inform the editor as soon as possible if proposed deadline to be exceeded	12
	40	Immediately communicate to journal when cannot perform review	9
	41	Suggest other reviewers if unable to review	7
Skilled critics	42	Provide constructive criticism	87
	43	Improve manuscript	84
	44	Be thorough/comprehensive/detailed/accurate	35
	45	Be critical/sceptical: evaluate a manuscript in a critical manner	27
	46	Be specific: provide authors with specific guidance on how to improve their manuscript	26
	47	Support comments with evidence: reviewers should document their comments and substantiate their points by referring to appropriate references and resources	20
	48	Be clear: clearly explain concerns	14
	49	Provide relevant comments: offer meaningful and reasonable comments that can be addressed	12
	50	Be consistent with comments to authors and editors: comments provided to the authors should be in line with confidential comments provided to editor in order to facilitate editors’ decision-making, ensure consistency and avoid miscommunication.	11
	51	Be systematic and methodological	11
	52	Be balanced: provide a balanced critique	9
	53	Be logical: provide logical arguments	5
	54	Be concise/incisive	5
	55	Evaluate manuscripts in a consistent manner	4
	56	Have intuitive capacity to detect faults and recognise quality	2
Respectful communicators	57	Be polite/courteous/respectful in the communication with authors	41
	58	‘Do unto others as you would have them do unto you’: treat others as we expect to be treated	22
	59	Be positive: peer reviews should be written in a positive attitude and offer praise for work well done	13
	60	Be nice/kind/considerate	12
	61	Be helpful: provide helpful comments	12
	62	Be collegial: treat each manuscript as if it had been written by a valued colleague	8
Gatekeepers	63	Maintain and improve manuscript quality and scientific rigour	15
	64	Weed out unsuitable manuscripts that are not scientifically valid	11
Educators	65	Educate and mentor authors: provide a learning opportunity	15
	66	Encourage authors: peer reviewers should encourage authors to improve manuscript	11
Advocates for author/editor/reader	67	Be an advocate for the editor	6
	68	Be an advocate for the author	6
	69	Be an advocate to readers	2
Advisors to editors	70	Advise editors on the merits of manuscripts	40
	71	Provide confidential comments to editor	32
Peer reviewers should not…	72	Be decision makers: they should acknowledge that the final decision on the publication of a manuscript rests with the editor	22
	73	Be copy editors (i.e. offer editorial comments about grammar and spelling)	21
	74	Ask for unreasonable or pivotal change	11
	75	Be overtly critical or too detailed: peer reviewers should not be generous and should not ‘nit-pick’ or overwhelm the authors	9
	76	Add additional requests in subsequent reviews that are not related to the original revisions	2

^a^Corresponds to the ‘Role item(s)’ columns in the tables related to roles in the additional files

^b^Number of extracted roles statements across all data sources in the scoping review

**Table 2:** Task-related statements (‘tasks’ refer to specific actions that fulfil ‘roles’ that refer to the overarching nature of peer reviewers’ function. The statements are ranked by numerical frequency)

Theme	Item^a^	Tasks…	#^b^
Organisation and approach to review	1	Identify strengths and weaknesses	31
	2	Identify flaws	29
	3	Provide summary of key points	29
	4	Differentiate between major and minor comments	17
	5	Follows reviewer guidelines provided by the journal	11
	6	Differentiate between fatal vs. addressable flaws	10
	7	Address all aspects of the manuscript	9
	8	Differentiate between general and specific comments	6
	9	Identify missing information	5
	10	Number each statement chronologically	5
Make general comments	11	Determine validity/quality/technical merit/rigour	69
	12	Assess originality	55
	13	Assess novelty	54
	14	Assess importance/significance	48
	15	Comment upon relevance to practice/science (clinical relevance)	45
	16	Comment upon contribution to the field	42
	17	Highlight whether current literature is covered	35
	18	Determine timeliness of the manuscript—is it topical?	16
	19	Determine whether reporting guidelines were followed (i.e. appropriate selection and adherence by authors)	5
	20	Comment upon conceptual/theoretical framework	4
Assess and address content for each section of the manuscript	Title		
	21	Title is accurate	28
	Abstract		
	22	Accurate/conclusions consistent with results	26
	23	Sufficiently detailed	23
	24	Adequacy of abstract (in general)	18
	25	Use of salient keywords	7
	Introduction		
	26	Clarity of study purpose and hypothesis	50
	27	Adequacy of introduction (in general)	37
	28	Appropriateness and adequacy of the literature review	22
	29	Relevance of problem	19
	Methods		
	30	Adequacy of methods (in general)	65
	31	Study design	56
	32	Data analysis (methods and tests)	42
	33	Use of statistics	42
	34	Sampling strategy	34
	35	Clarity and validity of statistical methods	33
	36	How data was collected/reproducibility of methods	33
	37	Methods appropriate for the research question	29
	38	Risk of bias	25
	39	Definition and measurement of variables	22
	40	Inclusion/exclusion criteria	15
	41	Follow-up	12
	42	Assess different analysis parts separately	11
	43	Reliable and appropriate tools used	11
	44	Power analysis	10
	Results		
	45	Clarity of tables and figures	54
	46	Adequacy of results (general)	46
	47	Neutral and logical presentation of results	25
	48	No interpretation of results	12
	49	Accuracy of raw data/appendices	8
	Discussion/conclusion		
	50	Interpretation supported by data	92
	51	Adequacy of discussion (general)	53
	52	Study limitations addressed	22
	53	Research and policy implications (suggestions for future studies)	17
	54	Summary reflects contents of the article	13
	55	Generalizability of study conclusions	5
	References		
	56	Appropriateness and accuracy of references	52
Address ethical aspects	57	Consider general ethical aspects and report on any specific ethical concerns (including manipulation of data, plagiarism, duplicate publication, inappropriate treatment of animal or human subjects)	55
	58	Report on ethical approval	11
	59	Check specifically for plagiarism/fraud	4
	60	Highlight competing interests of authors	4
	61	No need to detect fraud	2
Assess manuscript presentation	62	Overall readability	41
	63	Presentation (general)	40
	64	Coherence/clarity and logical flow of the text	37
	65	Grammar and spelling	30
	66	Organisation of the manuscript	25
	67	Use of language	21
	68	Length of the manuscript	12
	69	Check adherence to authors’ guidelines (i.e. journal guidelines for authors)	9
Provide recommendations	70	Recommendations on publication (e.g. no/minor/major revisions, reject)	74
	71	Comment on interest to journal readership/relevance for journal scope	52
	72	Complete (numerical) rating/checklist	26
	73	Recommend another more suitable journal	2

^a^Corresponds to the ‘Role item(s)’ columns in the tables related to tasks in the additional files

^b^Number of extracted tasks statements across all data sources in the scoping review

A total of 1462 statements related to roles were extracted, resulting in 76 unique statements. These were grouped into 13 emergent themes where peer reviewers were considered to be proficient experts in their field (3 items), dutiful/altruistic towards scientific community (7 items), familiar with journal (2 items), unbiased and ethical professionals (18 items), self-critical professionals (4 items), reliable professionals (7 items), skilled critics (15 items), respectful communicators (6 items), gatekeepers (2 items), educators (2 items), advocates for author/editor/reader (3 items) and advisors to editors (2 items). Roles that do not fall within the remit of peer reviewers were also identified (5 items).

**Fig. 2:**
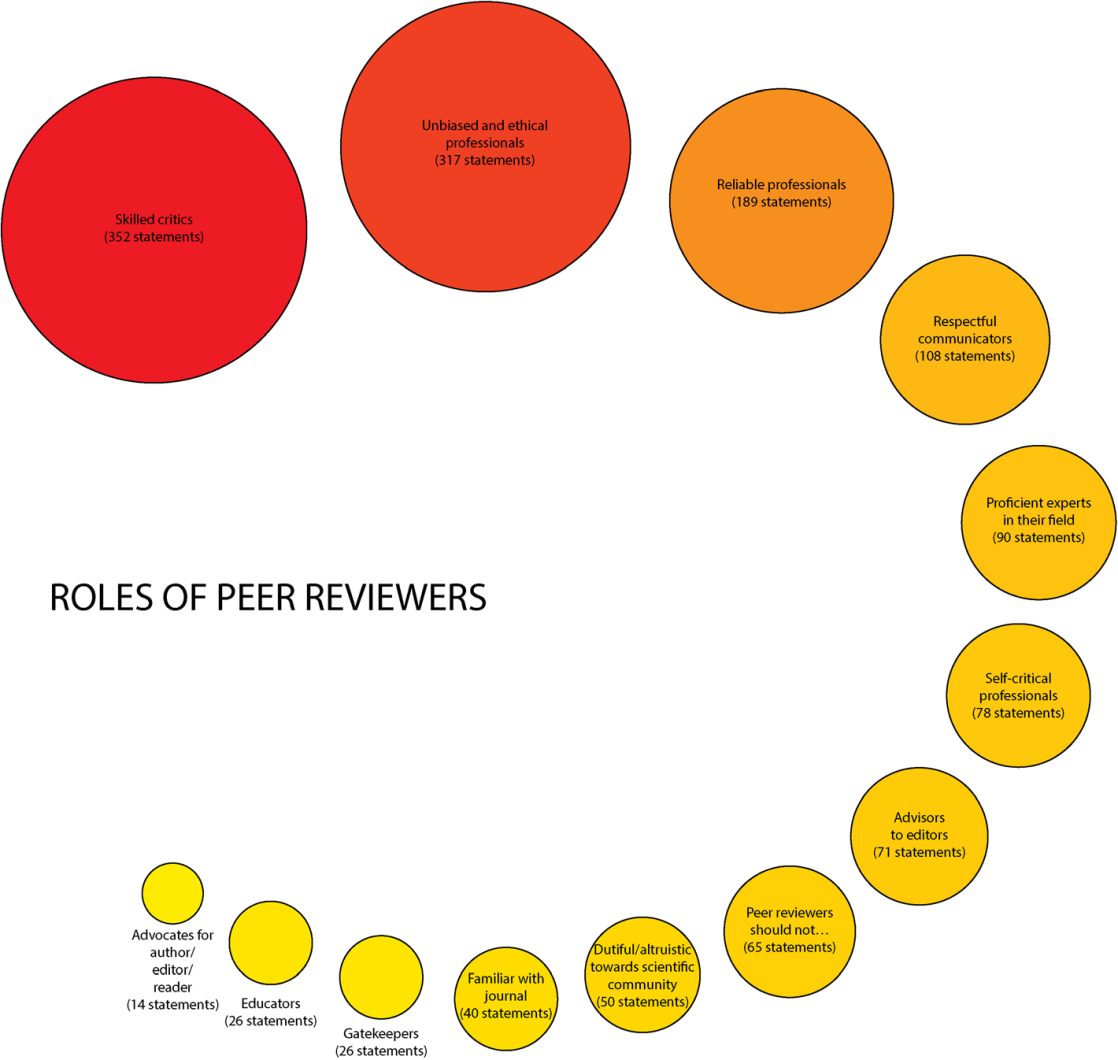
Themes related to roles of peer reviewers

The ‘skilled critics’ and ‘unbiased and ethical professionals’ themes appeared most frequently. Figure 2 shows the identified themes according to the number of associated statements, with larger circles denoting a higher number.

**Fig. 3:**
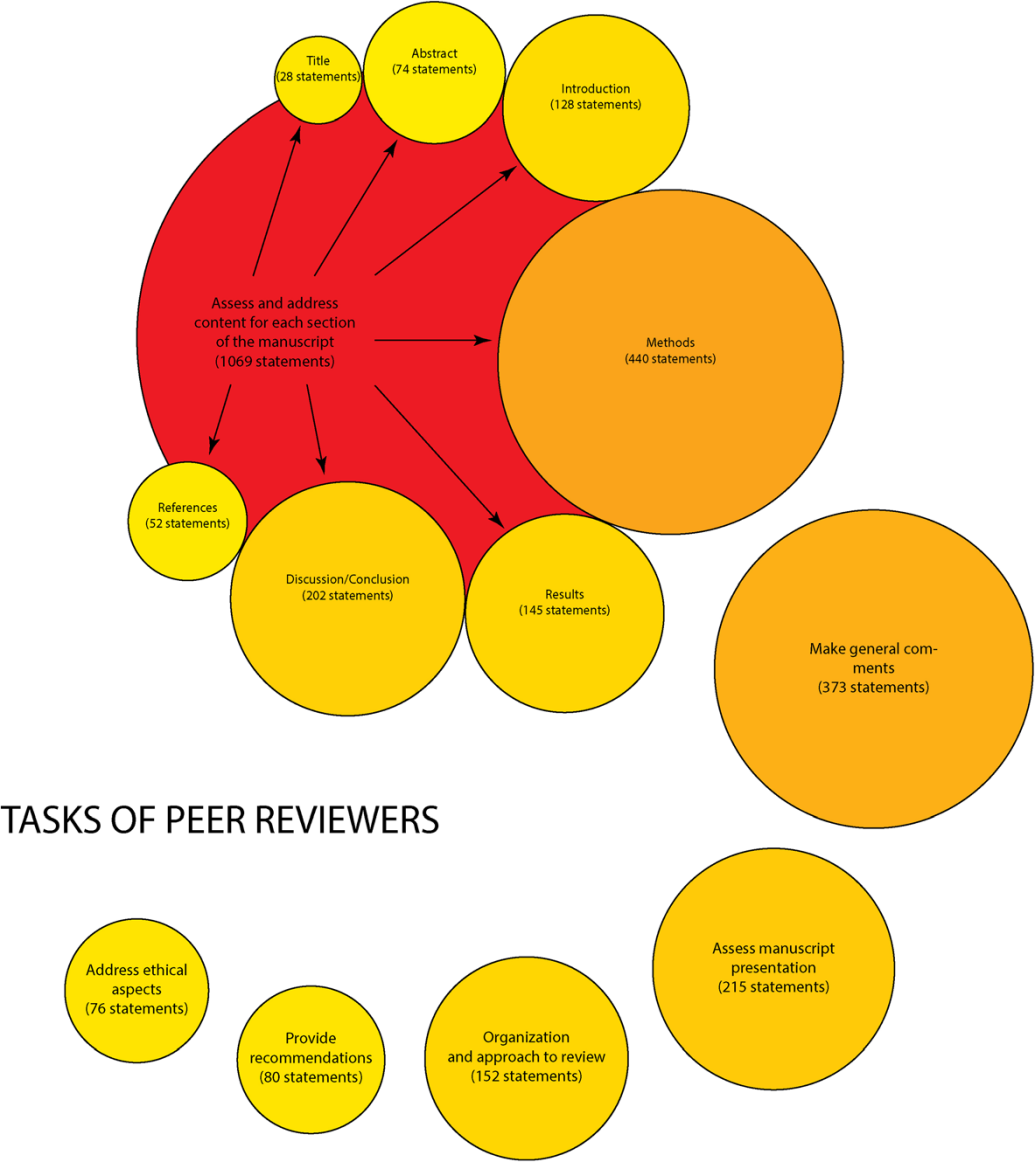
Themes related to tasks of peer reviewers

We also extracted 2026 statements related to peer reviewers’ tasks, resulting in 73 unique statements. These were grouped under six themes: organisation and approach to reviewing (10 items), make general comments (10 items), assess and address content for each section of the manuscript (36 items), address ethical aspects (5 items), assess manuscript presentation (8 items) and provide recommendations (4 items). The themes ‘assess and address content for each section of the manuscript’ had the highest number of statements while the theme related to ethical aspects had the lowest number (Fig. 3).

## Discussion

This scoping review produced a comprehensive list of roles and tasks of peer reviewers, derived from a wide range of sources. We sought to complement an existing scoping review on competencies for scientific editors of biomedical journals [8]. While the focus of the scoping review is biomedicine, it is possible that many of the roles and tasks identified could apply more broadly to the discipline of science (e.g. Science, Technology, Engineering and Mathematics (STEM)).

From our analysis, we found incongruities between the position of the peer reviewer and the position of the editor as reported in the literature. For example, the link between peer reviewers’ recommendations and editorial decision-making—where the former typically informs the latter—is often unclear. One of the roles identified in this article is for peer reviewers to keep in mind that they are not decision-makers regarding the ultimate fate of the manuscript. Such decision making is typically made by the editors who ‘Synthesize reviews and make ultimate editorial decisions in light of peer reviewers’ comments and other editors’ comments’ [9], to take this decision. At the same time, a key reviewer task emerging from this scoping review relates to the provision of a recommendation regarding the manuscript (decision-making: reject, accept, etc.). While peer reviewers should be expected to offer advice to editors on the merits of a manuscript, reviewer recommendations around whether or not to publish the manuscript might actually have a more substantial impact on final editorial decision-making than intended, thereby endangering any aspirations of editorial independence. Research indicates that peer reviewer recommendations have a direct influence on editorial decisions [20]. This becomes a problem when the quality of peer reviewer reports is questionable or when one of the many forms of bias that reviewers may display is present. Lee et al. describe bias as a ‘function of author characteristics’ which includes prestige bias, affiliation bias, nationality bias, language bias and gender bias. There might also be ‘bias as a function of reviewer characteristics’, such as when peer reviewers display content-based bias, confirmation bias, conservatism, bias against interdisciplinary research and publication bias [21].

Evidence from the field of meta-research—the study of science itself—indicates that the biomedical literature is replete with low-quality publications [20, 21] which have evidently successfully passed peer review. This in turn suggests that the filtering function of journal editors is not being properly fulfilled. Thus, the question is whether the responsibility for publication of low-quality manuscripts is shared by both peer reviewers and editors or whether it should be borne solely by editors.

Another example of tension between these two stakeholders is the overlap that exists across certain tasks. For example, a key competency of journal editors should be to ‘evaluate the scientific rigor and integrity of manuscripts’ [9]. A study that analysed editorial discourse in a high-impact journal to provide insight into editorial decision-making found that factors related to science—such as research design and methods—were most often addressed during the internal discussion among the editors [22] . Concurrently, peer reviewers are expected to dedicate time to the evaluation and scoring of manuscripts’ scientific rigour and integrity, in what seems to be a duplication of effort for potentially limited impact. Another perspective on this could be that the editor has made an evaluation and is asking the peer reviewers to do likewise, so it could be perceived as a validation of the editor’s views concerning the scientific validity of the submission. It could also be seen as a way of using collective intelligence during the review process. However, peer reviewers are primarily consulted as experts in their field whose knowledge and expertise can at times be broader than the editors’. Thus, the pertinent question here revolves around their authority as experts and whether they are simply rubber-stamping the editors’ decision.

Furthermore, the advocacy role of peer reviewers appeared several times in the included literature. According to some included articles, peer reviewers are variously expected to be advocates for authors, editors and/or readers. Whether this is a justifiable (and feasible) role is open to debate. Is it possible to be an advocate to all stakeholders simultaneously? If not, which stakeholders should take precedence and what would the order of priority be? The term ‘advocates’ needs to be unpacked and clarified in order for peer reviewers to understand what is expected from them in this particular area.

This overlap of roles, and the existence of apparently malleable boundaries between editors and peer reviewers, may have significant implications for the overall peer review process because there is the potential for misunderstandings to occur, as shown by Chauvin et al. [6]. Given the three problematic discrepancies described above, questions remain around their implications for reviewers’ roles and tasks in terms of authority and responsibility.

The 13 themes identified in the list of reviewers’ roles (Table 1) provided a clear construct of the ideal peer reviewer. Box 1 summarises these attributes, based on the most frequent statements identified in our review and the incongruities between the position of the peer reviewer and the position of the editor. Although this is a useful summary of the literature included, these roles need to be discussed critically beyond merely listing them for academic purposes. Instead, a more holistic approach, where these roles are critically discussed within the context of the broader scientific publication system in a way that acknowledges and recognises the complex and dynamic social relations that characterise the peer review process, should be adopted. Box 2 poses questions about the incongruities between the position of the peer reviewer and editor that need to be critically examined.

Complementary to the roles above, we identified 73 unique tasks that peer reviewers may variously be expected to perform. The large number of potential tasks identified is arguably excessive, especially since the majority of peer reviewers are not paid to perform these tasks and often receive little recognition for their work [23]. However, not all journals share all of these expectations. For example, a recent study on content of grading forms across a range of surgical journals found considerable variation in content, with relatively few journals requiring reviewers to address specific components of a manuscript. The study suggests that substantial variation exists in the grading criteria used to evaluate manuscripts submitted for peer review in this field, with a different emphasis placed on certain criteria correlated to journal impact factors. Grading forms of higher impact factor surgical journals more frequently addressed statistical analysis, ethical considerations and conflict of interest, whereas lower impact factor journals more commonly requested qualitative assessments of novelty or originality, scientific validity and scientific importance [24].

We also observed several contradictions. For example, there was some discrepancy as to whether detection of misconduct and fraud should fall within the remit of peer reviewers. However, in order to be able to detect fraud, it is likely that reviewers would need to check and verify the raw data of a study. Besides being impractical, this would almost certainly discourage prospective reviewers from participating in the already time-consuming peer review process. Research suggests that a small portion of the scientific community is already carrying out a disproportionate amount of peer reviewing [25], with the potential of contributing to downgraded peer review standards. Furthermore, journals often have more opportunities to check certain aspects related to misconduct, for example by using software to detect plagiarism. A study that identified ‘highly rated’ competency-related statements for biomedical editors found widespread agreement among editors that identifying and addressing allegations of fraud or plagiarism was a key competency [26] that should be performed by the editor, not by the peer reviewer.

There was also discrepancy regarding whether peer reviewers should engage in copy editing. Although the majority of included articles stated that copy editing does not fall within the duty of peer reviewers, several articles specifically mentioned that reviewers should offer grammatical and linguistic improvements. One could argue that this is the role of the copy editing team members at the journal, who are specifically trained to identify and address such aspects of the manuscript, whereas peer reviewers might not necessarily be sufficiently familiar with linguistic nuances to do so. The time potentially taken up by copy editing is also worth considering. A study found that lack of time is the principal factor for peer reviewers of biomedical journal in their decision to decline a peer review [27]. The time of peer reviewers is precious; therefore, their primary focus should be on the improvement of scientific content rather than the linguistic fine-tuning.

We found variation in the level of detail provided. Certain tasks were vaguely described. For example, statements such as ‘check adequacy of abstract’ or ‘assess manuscript presentation’ were not specific enough in terms of what exactly is required. Such generic statements are not helpful in explaining what editors expect, particularly to new or inexperienced peer reviewers. Vague guidance may result in vague peer review. One simple but straightforward way of addressing this would be to thoroughly review and revise guidance provided to peer reviewers.

The term ‘advocate’ appeared several times in the included literature. According to some included articles, peer reviewers are variously expected to be advocates for authors, editors and/or readers. Whether this is a justifiable role is open to debate. Is it possible to be an advocate to all stakeholders? If not, which stakeholders should take precedence and what would the order of priority be? The term ‘advocates’ needs to be unpacked and clarified in order for peer reviewers to understand what is expected from them in this particular area.

Based on findings from a preceding scoping review of competencies for scientific editors of biomedical journals [8], it was anticipated that a substantial proportion of relevant statements would be identified in grey literature, rather than in peer-reviewed literature. Therefore, sources of grey literature were searched to supplement the database search strategy in the identification of task and role-related statements. Due to the sheer quantity of potentially relevant grey literature available on the web (e.g. websites of publishers), we have taken a pragmatic approach and focused on selected sources from official organisations that deal with peer review and which were also identified in the scoping review of editor competencies [8]. We also included some popular training courses for peer reviewers. However, we recognise that this is by no means comprehensive and we may have missed some potentially useful documents.

We were able to extract data from articles written in English, German, Spanish and Portuguese, but we are aware that the database and grey literature searches may not have included all available relevant literature due to language restrictions. Additionally, despite our best efforts, it is possible that we may have missed some aspects of peer reviewers’ roles and tasks in our search. We preserved the wording used by authors to describe roles and tasks wherever possible and tried to ensure that any changes to wording reflected the spirit of what was being said when editing was necessary. However, it is possible that at times the subjective and selective nature of data extraction may have resulted in occasional misinterpretation of authors’ intended statements. For example, some streamlining was necessary to ensure that the final list of roles and tasks was both manageable and useful; hence, it is possible that subtle differences between tasks or roles might have been smoothened out in an effort to remove redundant or overlapping items. We expect that some missing items will appear in the next stage of our research, where statements will be refined and expanded during a qualitative study with journal editors.

## Conclusion

To our knowledge, this scoping review is the first attempt to systematically identify possible roles and tasks of peer reviewers in biomedical journals. This is the counter piece of the existing scoping review on competencies for scientific editors of biomedical journals [8].

As a standalone research piece, this study will primarily be helpful in demonstrating the extent and nature of existing literature on this topic, as well as displaying the type of roles and tasks requested (Additional files 7 and 8). As such, this will be relevant to a variety of audiences, including publishers, editors, peer reviewers and authors. For example, journal editors may be inspired to review their instructions to peer reviewers, whereas course developers might opt to update the content of training courses for peer reviewers.

In addition, a possible training initiative could include the use of ‘open reports’ (i.e. peer review reports and the authors’ responses that are published alongside the relevant articles) which, according to a systematic review (2017) of the definitions of ‘open peer review’, is one of the seven main characteristics of open peer review [28]. These can be used as an educational tool for authors, editors and peer reviewers alike to unpick the different roles and tasks and to encourage a discussion on this subject. The reports can be prepared in such a way that they would reflect the emergent themes that we identified within our scoping review. Potential settings for such an educational intervention could be events such as faculty development meetings at Universities, where authors, editors and peer reviewers often mingle. The different themes could be presented using the concept of snippets [29] which are short, generally limited to 20–30 min. The focus of a snippet is a single overriding communication objective (SOCO). Our identified themes are well suited to be transformed into snippets and can be taught in the allotted time using carefully curated open reports. This review will also inform a subsequent qualitative study with journal editors, with the aim of gaining further insight into their understanding of peer reviewers’ roles and tasks [13] and eventually laying the groundwork for the development of a set of core competencies for peer reviewers of biomedical journals that could then be facilitated through a consensus exercise.

Box 1 Construct of a peer reviewer Reviewers should be proficient experts in their field with training in research methods or statistics who—out of a sense of scientific responsibility and duty—provide unbiased, objective, thorough and specific yet constructive criticism to authors on how to improve their manuscript. Importantly, prior to commencing reviewing, self-critical reviewers should be confident about their availability and competence to review and be familiar with the journal’s mission and review criteria and guidelines. Any potential or actual conflict of interest should be declared upfront or avoided entirely, in line with ethical norms. Reviewers should offer advice to editors on the merits of the manuscript while keeping in mind that they are not decision-makers regarding the fate of the manuscript. A polite, collegial attitude that promotes education of authors is vital. Lastly, they should maintain confidentiality throughout and deliver the report in a timely matter.

Box 2 Critical questions related to roles and tasks of peer reviewers - What is the link between peer reviewers’ recommendations and editorial decision-making?

- Who is responsible for publication of low-quality manuscripts?

- Is there overlap across certain roles and tasks (e.g. expert evaluation, advisors)?

- What are the consequences of existing malleable boundaries of authority and responsibility on the review process?

## Additional files

Additional file 1: Search strategy. (DOCX 21 kb)

Additional file 2: Original articles. (DOCX 62 kb)

Additional file 3: Reviews. (DOCX 122 kb)

Additional file 4: Editorials. (DOCX 241 kb)

Additional file 5: Grey literature. (DOCX 32 kb)

Additional file 6: Prisma_ScR_Checklist. (DOCX 105 kb)

Additional file 7: Data_Roles. (XLSX 188 kb)

Additional file 8: Data_Tasks. (XLSX 178 kb)

## Data Availability

All data generated or analysed during this study are included in this published article.

## Abbreviations

EQUATOR: Enhancing Quality and Transparency in Health Research
PRISMA-ScR: Preferred Reporting Items for Systematic reviews and Meta-Analyses extension for Scoping Reviews
RCT: Randomised controlled trial
SOCO: Single overriding communication objective
STEM: Science, Technology, Engineering and Mathematics
