# Supplementary material for: A scoping review on the roles and tasks of peer reviewers in the manuscript review process in biomedical journals
Source: BMC Med. 2019 Jun 20;17:118. doi: 10.1186/s12916-019-1347-0 (PMC6585141; doi:10.1186/s12916-019-1347-0)
Supplement: Supplementary file 1 — Search strategy. (DOCX 21 kb) [file 12916_2019_1347_MOESM1_ESM.docx]

**Additional file 1 – Search strategy**

**Scoping review on the roles and tasks of peer reviewers in the biomedical journal editorial process**

Databases: Ovid MEDLINE(R) Epub Ahead of Print, In-Process & Other Non-Indexed Citations, Ovid MEDLINE(R) Daily and Ovid MEDLINE(R) 1946 to Present (April 2017 Week 2)

| 1 | ((reviewing or reviewer or peer reviewer or peer-revie* or peer review) adj5 (abilit* or aptitud* or capabilit* or capacit* or character* or competen* or criteri* or educat* or effectiv* or evaluat* or expertise or integrit* or knowledg* or learning or proficien* or qualifi* or qualify or recommend* or responsibilit* or role or roles or skill or skills or standard or standards or talent* or task or tasks or training)).tw. |
| --- | --- |
| 2 | exp *peer review, research/ |
| 3 | professional competence/ |
| 4 | responsibility/ |
| 5 | 3 or 4 |
| 6 | 2 and 5 |
| 7 | 1 or 6 |

**4821** [MEDLINE UNIQUE HITS]

**12306** [EMBASE HITS]

**3647** [PSYCINFO HITS]

Cochrane Library

Search Name: Role and tasks – Peer reviewers

Date Run: 28/02/17

ID Search Hits

#1 ((reviewing or reviewer or peer reviewer or peer-revie* or peer review) near/5 (abilit* or aptitud* or capabilit* or capacit* or character* or competen* or criteri* or educat* or effectiv* or evaluat* or expertise or integrit* or knowledg* or learning or proficien* or qualifi* or qualify or recommend* or responsibilit* or role or roles or skill or skills or standard or standards or talent* or task or tasks or training)).ti,ab,kw

#2 MeSH descriptor: [Peer Review, Research] explode all trees

#1 or #2

Results **9164**

CINAHL

# Query Limiters/Expanders Last Run Via Results

S3 S1 AND S2 Search modes - Boolean/Phrase Interface - EBSCOhost Research Databases

Search Screen - Advanced Search

Database - CINAHL Plus with Full Text 1,112

S2 TI ( (reviewing or reviewer or peer reviewer or peer-revie* or peer review) N5 (abilit* or aptitud* or capabilit* or capacit* or character* or competen* or criteri* or educat* or effectiv* or evaluat* or expertise or integrit* or knowledg* or learning or proficien* or qualifi* or qualify or recommend* or responsibilit* or role or roles or skill or skills or standard or standards or talent* or task or tasks or training) OR AB (abilit* or aptitud* or capabilit* or capacit* or character* or competen* or criteri* or educat* or effectiv* or evaluat* or expertise or integrit* or knowledg* or learning or proficien* or qualifi* or qualify or recommend* or responsibilit* or role or roles or skill or skills or standard or standards or talent* or task or tasks or training) ) Search modes - Boolean/Phrase Interface - EBSCOhost Research Databases

Search Screen - Advanced Search

Database - CINAHL Plus with Full Text 1,076,628

S1 (MH "Edit and Review+") OR (MH "Peer Review+")

Search modes - Boolean/Phrase

Interface - EBSCOhost Research Databases

Search Screen - Advanced Search

Database - CINAHL Plus with Full Text **6,926**

ERIC

2017 Feb 12

Abstract:((reviewing or reviewer or peer reviewer or peer-revie* or peer review) AND (health or medical or biomedical)

**329 hits**

Scopus

(TITLE(peer-revie*) AND ABS((abilit* or aptitud* or capabilit* or capacit* or competen* or criteri* or educat* or effectiv* or evaluat* or expertise or knowledg* or proficien* or qualif* or recommend* or responsibilit* or role)) OR ABS((skill or standard or talent* or task or training)) AND NOT TITLE-ABS("systematic review")) AND ( LIMIT-TO(SUBJAREA,"MEDI " ) OR LIMIT-TO(SUBJAREA,"SOCI " ) OR LIMIT-TO(SUBJAREA,"NURS " ) OR LIMIT-TO(SUBJAREA,"HEAL " ) )

**1440 hits**

Web of Science

# 1

**3,497** TI=(peer review OR peer-review)

Refined by: WEB OF SCIENCE CATEGORIES: ( MEDICINE GENERAL INTERNAL OR BEHAVIORAL SCIENCES OR GASTROENTEROLOGY HEPATOLOGY OR MULTIDISCIPLINARY SCIENCES OR CARDIAC CARDIOVASCULAR SYSTEMS OR MEDICAL ETHICS OR EDUCATION EDUCATIONAL RESEARCH OR IMMUNOLOGY OR OBSTETRICS GYNECOLOGY OR HEALTH CARE SCIENCES SERVICES OR NURSING OR PEDIATRICS OR LINGUISTICS OR ORTHOPEDICS OR PSYCHOLOGY EDUCATIONAL OR EDUCATION SCIENTIFIC DISCIPLINES OR PSYCHOLOGY BIOLOGICAL OR PSYCHOLOGY DEVELOPMENTAL OR RADIOLOGY NUCLEAR MEDICINE MEDICAL IMAGING OR PATHOLOGY OR PSYCHIATRY OR OPHTHALMOLOGY OR MEDICAL INFORMATICS OR SOCIAL SCIENCES INTERDISCIPLINARY OR ENDOCRINOLOGY METABOLISM OR NEUROSCIENCES OR ONCOLOGY OR TOXICOLOGY OR PRIMARY HEALTH CARE OR SOCIAL ISSUES OR PSYCHOLOGY APPLIED OR MEDICINE RESEARCH EXPERIMENTAL OR SOCIAL SCIENCES BIOMEDICAL OR EMERGENCY MEDICINE OR HEALTH POLICY SERVICES OR ANESTHESIOLOGY OR HEMATOLOGY OR CRITICAL CARE MEDICINE OR TRANSPLANTATION OR CLINICAL NEUROLOGY OR PSYCHOLOGY OR ETHICS OR OTORHINOLARYNGOLOGY OR DENTISTRY ORAL SURGERY MEDICINE OR UROLOGY NEPHROLOGY OR RESPIRATORY SYSTEM OR SPORT SCIENCES OR PSYCHOLOGY CLINICAL OR PHYSIOLOGY OR DERMATOLOGY OR REHABILITATION OR HUMANITIES MULTIDISCIPLINARY OR PSYCHOLOGY MULTIDISCIPLINARY OR SOCIOLOGY OR PERIPHERAL VASCULAR DISEASE )

Indexes=SCI-EXPANDED, SSCI, A&HCI, CPCI-S, CPCI-SSH Timespan=All years
