# Supplementary material for: A scoping review on the roles and tasks of peer reviewers in the manuscript review process in biomedical journals
Source: BMC Med. 2019 Jun 20;17:118. doi: 10.1186/s12916-019-1347-0 (PMC6585141; doi:10.1186/s12916-019-1347-0)
Supplement: Supplementary file 2 — Original articles. (DOCX 62 kb) [file 12916_2019_1347_MOESM2_ESM.docx]

**Table 1.** Roles from original articles

| **Author** | **Country** | **Language** | **Journal** | **Year** | **Design** | **Theme(s)** | **Role items^b^** | **# of roles^c^** |
| --- | --- | --- | --- | --- | --- | --- | --- | --- |
| Bruce (1) | France | English | BMC Medicine | 2016 | Systematic review | N/A | N/A | 0 |
| Chauvin, A. (2) | France | English | BMC Medicine | 2015 | Survey | Familiar with journal | 11 | 1 |
| Das Sinha, S.(3) | India | English | National Medical Journal of India | 1999 | RCT | Proficient experts in their field,  Unbiased and ethical professionals,  Skilled critics,  Respectful communicators | 1, 15, 42, 44, 57 | 5 |
| Duchesne, S. (4) | Canada | English | Neuroimage | 2008 | Descriptive study | Unbiased and ethical professionals,  Reliable professionals,  Skilled critics,  Respectful communicators,  Advisors,  Peer reviewers should not | 16, 35, 43, 44, 47, 52, 57, 71, 72 | 9 |
| Frank, E. (5) | USA | English | Preventive Medicine | 1996 | Descriptive study | Unbiased and ethical professionals,  Reliable professionals,  Skilled critics,  Respectful communicators,  Advisors | 13, 14, 16, 21, 35, 38, 44, 46, 57, 71 | 10 |
| Freda, M.(6) | USA | English | Journal of Professional Nursing | 2009 | Survey | Proficient experts in their field,  Unbiased and ethical professionals,  Reliable professionals,  Skilled critics,  Educators,  Advisors,  Peer reviewers should not | 1, 16, 35, 42, 43, 44, 46, 66, 70, 73 | 10 |
| Goodman, S.N. (7) | USA | English | Annals Intern Medicine | 1994 | Masked before-after study | N/A | N/A | 0 |
| Henly, S. J.(8) | USA | English | Nursing Outlook | 2009 | Descriptive study | Unbiased and ethical professionals,  Skilled critics,  Respectful communicators,  Advisors,  Peer reviewers should not | 15, 42, 43, 44, 47, 57, 64, 70, 71 | 9 |
| Ho, R. C.-M.(9) | Hong Kong | English | BMC Medical Research Methodology | 2013 | Survey | Skilled critics,  Gatekeepers,  Educators | 42, 63, 66 | 3 |
| Johnston, S. C. (10) | N/A | English | Annals of Neurology | 2007 | Randomized trial | Skilled critics | 43 | 1 |
| Kearney, M. H.(11) | USA | English | Research in Nursing Health | 2005 | Survey | Proficient experts in their field,  Unbiased and ethical professionals,  Reliable professionals,  Skilled critics,  Respectful communicators,  Educators,  Advisors | 1, 15, 16, 35, 42, 43, 44, 46, 50, 52, 59, 60, 61, 66, 70, 73 | 16 |
| Kelly, M. J.(12) | UK | English | Colorectal Disease | 2013 | Intervention | Dutiful/altruistic towards scientific community | 6 | 1 |
| Kliewer, M. A.(13) | USA | English | American Journal of Roentgenology | 2004 | Descriptive study | Assess manuscript presentation | 62 | 1 |
| Kliewer, M. A.(14) | USA | English | American Journal of Roentgenology | 2005 | Survey | Unbiased and ethical professionals,  Self-critical professionals,  Reliable professionals, Assess manuscript presentation | 15, 33, 37 | 3 |
| Kurihara, Y.(15) | Japan | English | American Journal of Roentgenology | 2013 | Descriptive study | Skilled critics | 42, 43 | 2 |
| Larson, B. P.(16) | USA | English | HAND | 2012 | Systematic review | Unbiased and ethical professionals | 17 | 1 |
| Lipworth, W. L.(17) | Australia | English | Social Science & Medicine | 2011 | Qualitative study | Dutiful/altruistic towards scientific community,  Unbiased and ethical professionals,  Reliable professionals,  Skilled critics,  Respectful communicators | 4, 15, 16, 35, 42, 45, 48, 50, 51, 53, 55, 60 | 12 |
| Mulligan, A.(18) | N/A | English | Oral Oncology | 2005 | Qualitative study | Dutiful/altruistic towards scientific community,  Reliable professionals,  Skilled critics | 4, 35, 42, 55 | 4 |
| Napolitani, F.(19) | Italy | English | European Journal of Internal Medicine | 2016 | Descriptive study | Unbiased and ethical professionals,  Self-critical professionals | 13, 16, 29, 31, 33 | 5 |
| Rostami, K.(20) | UK | English | Gastroenterology and Hepatology | 2011 | Descriptive study | Familiar with journal,  Unbiased and ethical professionals,  Self-critical professionals,  Reliable professionals,  Skilled critics,  Assess manuscript presentation, | 11, 13, 16, 31, 37, 42, 44, 51 | 8 |
| Schroter, S.(21) | UK | English | Journal of the Royal Society of Medicine | 2008 | RCT | Skilled critics | 43 | 1 |
| Shattell, M. M.(22) | USA | English | Journal of Nursing Scholarship | 2010 | Survey | Familiar with journal, Skilled critics | 11, 42, 43, 44 | 4 |
| Siegelman, S. S.(23) | N/A | English | Radiology | 1991 | Descriptive study | Skilled critics,  Advisors to editors | 43, 70 | 2 |
| Vintzileos, A. M.(24) | USA | English | Journal of Maternal-Fetal & Neonatal Medicine | 2013 | Survey | Reliable professionals, Skilled critics | 36, 42, 44 | 3 |
| **Total** |  |  |  |  |  |  |  | **110** |

^B^ Corresponds to item number from the list of roles (Table 1)

^C^ Number of extracted roles statements

**Table 2.** Tasks from original articles

| **Author** | **Country** | **Language** | **Journal** | **Year** | **Design** | **Task domain** | **Task items^b^** | **# of tasks^c^** |
| --- | --- | --- | --- | --- | --- | --- | --- | --- |
| Bruce, R. (1) | France | English | BMC Medicine | 2016 | Systematic review | Make general comments,  Results  +systematic review items | 11, 13, 15, 46 | 4 |
| Chauvin, A.(2) | France | English | BMC Medicine | 2015 | Survey | Organization and approach to review,  Make general comments,  Abstract,  Methods,  Results,  Discussion/Conclusion, References,  Address ethical aspects,  Assess manuscript presentation,  Provide recommendations,  + RCT specific items | 5, 13, 14, 15, 16, 17, 19, 22, 23, 30, 31, 33, 34, 36, 38, 39, 45, 47, 48, 49, 50, 52, 55, 56, 57, 58, 59, 64, 65, 67, 70, | 31 |
| Das Sinha, S.(3) | India | English | National Medical Journal of India | 1999 | RCT | Organization and approach to review,  Make general comments,  Results,  Discussion/Conclusion,  Assess manuscript presentation,  Provide recommendations, | 1, 6,  14, 31, 32, 36, 45, 50, 51, 63, 67, 70, 72, 73 | 14 |
| Duchesne, S.(4) | Canada | English | Neuroimage | 2008 | Descriptive study | Organization and approach to review,  Make general comments,  Title is accurate, Abstract,  Introduction,  Results,  Discussion/Conclusion, References,  Address ethical aspects,  Assess manuscript presentation,  Provide recommendation  + Medical imaging items | 4, 12, 13, 15, 16, 21, 24, 25, 26, 28, 29, 31, 32, 33, 34, 35, 36, 38, 40, 45, 49, 50, 52, 53, 55, 56, 57, 58, 64, 65, 67, 68, 71, 72 | 34 |
| Frank, E.(5) | USA | English | Preventive Medicine | 1996 | Descriptive study | Make general comments,  Title is accurate, Abstract,  Introduction,  Methods,  Results,  Discussion/Conclusion, References,  Address ethical aspects,  Assess manuscript presentation,  Provide recommendations | 11, 13, 14, 21, 24, 25, 27, 30, 33, 45, 46, 50, 51, 52, 56, 57, 60, 64, 70, 71 | 20 |
| Freda, M.(6) | USA | English | Journal of Professional Nursing | 2009 | Survey | Organization and approach to review, Make general comments,  Methods,  Provide recommendations | 1, 15, 17, 30, 70 | 5 |
| Goodman, S.N.(7) | USA | English | Annals Intern Medicine | 1994 | Masked before-after study | Make general comments,  Title is accurate, Abstract,  Introduction,  Methods,  Results,  Discussion/Conclusion,  Assess manuscript presentation | 15, 16, 21, 24, 26, 28, 30, 31, 34, 35, 37, 39, 45, 52, 55, 63, 66 | 17 |
| Henly, S. J.(8) | USA | English | Nursing Outlook | 2009 | Descriptive study | Make general comments, Introduction,  Methods,  Results,  Discussion/Conclusion,  References,  Assess manuscript presentation,  Provide recommendations | 11, 12, 14, 16, 20, 27, 30, 31, 32, 47, 51, 56, 64, 66, 67, 70, 72 | 17 |
| Ho, R. C.-M.(9) | Hong Kong | English | BMC Medical Research Methodology | 2013 | Survey | Make general comments,  Methods, Discussion/Conclusion, Provide recommendations | 11, 31, 33, 37, 50, 70 | 6 |
| Johnston, S. C.(10) | N/A | English | Annals of Neurology | 2007 | Randomized trial | Make general comments,  Provide recommendations | 15, 70 | 2 |
| Kearney, M. H.(11) | USA | English | Research in Nursing Health | 2005 | Survey | Organization and approach to review,  Make general comments,  Methods,  Address ethical aspects,  Provide recommendations | 1, 11, 15, 16, 17, 31, 43, 57, 70 | 9 |
| Kelly, M. J.(12) | UK | English | Colorectal Disease | 2013 | Intervention | Make general comments,  Abstract,  Introduction, Methods, Discussion/Conclusion,  Address ethical aspects,  Assess manuscript presentation | 12, 14, 15, 23, 26, 33, 37, 46, 51, 57, 63, 65 | 12 |
| Kliewer, M. A.(14) | USA | English | American Journal of Roentgenology | 2004 | Descriptive study | Organization and approach to review,  Make general comments,  Methods,  Results, Discussion/Conclusion, Assess manuscript presentation,  Provide recommendations | 2, 11, 15, 16, 26, 31, 32, 45, 50, 51, 62, 64, 65, 66, 70, 72 | 16 |
| Kliewer, M. A.(13) | USA | English | American Journal of Roentgenology | 2005 | Survey | Make general comments,  Assess manuscript presentation,  Provide recommendations | 11, 14, 62, 64, 71 | 5 |
| Kurihara, Y.(15) | Japan | English | American Journal of Roentgenology | 2013 | Descriptive study | Make general comments,  Provide recommendations | 11, 70 | 2 |
| Larson, B. P.(16) | USA | English | HAND | 2012 | Systematic review | Make general comments,  Title is accurate, Abstract,  Introduction, Methods,  Results, Discussion/Conclusion,  Address ethical aspects,  Assess manuscript presentation | 11, 12, 13, 21, 22, 23, 26, 28, 31, 33, 35, 37, 39, 43, 45, 47, 48, 50, 52, 53, 54, 56, 64, 65, 67, 69 | 26 |
| Lipworth, W. L.(17) | Australia | English | Social Science & Medicine | 2011 | Qualitative study | N/A | N/A | 0 |
| Mulligan, A.(18) | N/A | English | Oral Oncology | 2005 | Qualitative study | Address ethical aspects | 61 | 1 |
| Napolitani, F.(19) | Italy | English | European Journal of Internal Medicine | 2016 | Descriptive study | N/A | N/A | 0 |
| Rostami, K.(20) | UK | English | Gastroenterology and Hepatology | 2011 | Descriptive study | Make general comments,  Abstract, Introduction, Methods, Discussion/Conclusion, References,  Address ethical aspects,  Assess manuscript presentation,  Provide recommendations | 15, 16, 24, 26, 28, 29, 30, 31, 32, 33, 34, 36, 39, 40, 46, 47, 50, 51, 56, 57, 62, 63, 70 | 23 |
| Schroter, S.(21) | UK | English | Journal of the Royal Society of Medicine | 2008 | RCT | Organization and approach to review, Introduction, Methods,  Results,  Discussion/Conclusion,  Address ethical aspects,  Provide recommendations | 2, 4, 26, 30, 32, 34, 38, 39, 45, 50, 51, 56, 72 | 13 |
| Shattell, M. M.(22) | USA | English | Journal of Nursing Scholarship | 2010 | Survey | Organization and approach to review, Provide recommendations | 2, 5,  7, 70 | 4 |
| Siegelman, S. S.(23) | N/A | English | Radiology | 1991 | Descriptive study | Make general comments, Provide recommendations | 12, 15, 72 | 3 |
| Vintzileos, A. M.(24) | USA | English | Journal of Maternal-Fetal & Neonatal Medicine | 2013 | Survey | N/A  Systematic review specific items | N/A | 0 |
| **Total** |  |  |  |  |  |  |  | **264** |

^b^ Corresponds to item number from the list of tasks (Table 2)

^c^ Number of extracted roles statement

## References

1. Bruce R, Chauvin A, Trinquart L, Ravaud P, Boutron I. Impact of interventions to improve the quality of peer review of biomedical journals: a systematic review and meta-analysis. BMC Med. 2016;14(1):85.

2. Chauvin A, Ravaud P, Baron G, Barnes C, Boutron I. The most important tasks for peer reviewers evaluating a randomized controlled trial are not congruent with the tasks most often requested by journal editors. BMC Med. 2015;13(1):1.

3. Das Sinha S, Sahni P, Nundy S. Does exchanging comments of Indian and non-Indian reviewers improve the quality of manuscript reviews? Natl Med J India. 1999;12.

4. Duchesne S, Jannin P. Proposing a manuscript peer-review checklist. Neuroimage. 2008;39(4):1783–7.

5. Frank E. Editors’ requests of peer reviewers: a study and a proposal. Prev Med. 1996;25(2):102–4.

6. Freda MC, Kearney MH, Baggs JG, Broome ME, Dougherty M. Peer Reviewer Training and Editor Support: Results From an International Survey of Nursing Peer Reviewers. J Prof Nurs. 2009 Mar 1;25(2):101–8.

7. Goodman SN, Berlin J, Fletcher SW. Manuscript quality before and after peer review and editing at Annals of Internal Medicine. Ann Intern Med [Internet]. 1994;121. Available from: http://dx.doi.org/10.7326/0003-4819-121-1-199407010-00003

8. Henly SJ, Dougherty MC. Quality of manuscript reviews in nursing research. Nurs Outlook. 2009;57(1):18–26.

9. Ho RC-M, Mak K-K, Tao R, Lu Y, Day JR, Pan F. Views on the peer review system of biomedical journals: an online survey of academics from high-ranking universities. BMC Med Res Methodol. 2013 Jun 7;13(1):74.

10. Johnston SC, Lowenstein DH, Ferriero DM. Early editorial manuscript screening versus obligate peer review: a randomized trial. Ann Neurol [Internet]. 2007;61. Available from: http://dx.doi.org/10.1002/ana.21150

11. Kearney MH, Freda MC. Nurse editors’ views on the peer review process. Res Nurs Health. 2005;28(6):444–52.

12. Kelly M, Thomas W. Does tuition for journal referees work? A quantitative evaluation of a half‐day tuition course. Colorectal Dis. 2013;15(6):755–7.

13. Kliewer MA, Freed KS, DeLong DM, Pickhardt PJ, Provenzale JM. Reviewing the reviewers: comparison of review quality and reviewer characteristics at the American Journal of Roentgenology. AJR Am J Roentgenol. 2005 Jun;184(6):1731–5.

14. Kliewer MA, DeLong DM, Freed K, Jenkins CB, Paulson EK, Provenzale JM. Peer review at the American Journal of Roentgenology: How reviewer and manuscript characteristics affected editorial decisions on 196 major papers. Am J Roentgenol. 2004 Dec;183(6):1545–50.

15. Kurihara Y, Colletti PM. How Do Reviewers Affect the Final Outcome? Comparison of the Quality of Peer Review and Relative Acceptance Rates of Submitted Manuscripts. Am J Roentgenol. 2013 Sep;201(3):468–70.

16. Larson BP, Chung KC. A systematic review of peer review for scientific manuscripts. Hand N Y N. 2012 Mar;7(1):37–44.

17. Lipworth WL, Kerridge IH, Carter SM, Little M. Journal peer review in context: A qualitative study of the social and subjective dimensions of manuscript review in biomedical publishing. Soc Sci Med 1982. 2011 Apr;72(7):1056–63.

18. Mulligan A. Is peer review in crisis? Oral Oncol. 2005 Feb;41(2):135–41.

19. Napolitani F, Petrini C, Garattini S. Ethics of reviewing scientific publications. Eur J Intern Med. 2016 Dec 27;27:27.

20. Rostami K, Khadjooi K, Ahasaeed-Elhag R, Ishag S. How to evaluate a manuscript for publication? Gastroenterol Hepatol Bed Bench. 2011 Apr;4(2):58–62.

21. Schroter S, Black N, Evans S, Godlee F, Osorio L, Smith R. What errors do peer reviewers detect, and does training improve their ability to detect them? J R Soc Med. 2008;101(10):507–14.

22. Shattell MM, Chinn P, Thomas SP, Cowling WR. Authors’ and Editors’ Perspectives on Peer Review Quality in Three Scholarly Nursing Journals. J Nurs Scholarsh. 2010;42(1):58–65.

23. Siegelman SS. Assassins and Zealots - Variations in Peer-Review - Special Report. Radiology. 1991 Mar;178(3):637–42.

24. Vintzileos AM, Carvajal J, Islam S. Challenges in the peer review of systematic reviews and meta-analyses. J Matern Fetal Neonatal Med. 2013;26(8):768–71.
