# Supplementary material for: A scoping review on the roles and tasks of peer reviewers in the manuscript review process in biomedical journals
Source: BMC Med. 2019 Jun 20;17:118. doi: 10.1186/s12916-019-1347-0 (PMC6585141; doi:10.1186/s12916-019-1347-0)
Supplement: Supplementary file 4 — Editorials. (DOCX 241 kb) [file 12916_2019_1347_MOESM4_ESM.docx]

**Additional file 2**

**Table 1** Roles identified in book chapters, commentaries, editorials, letters, perspectives, and tutorials

| **Author** | **Country** | **Language** | **Journal** | **Year** | **Design** | **Theme(s)** | **Role(s)** | **# of role(s)** |
| --- | --- | --- | --- | --- | --- | --- | --- | --- |
| Alam, S.(1) | UK | English | BMC Medicine | 2015 | Editorial | Self-critical professionals,  Skilled critics,  Advisors | 32, 44, 70 | 3 |
| Allen, T. W.(2) | USA | English | The Journal of the American Osteopathic Association | 2013 | Commentary | Familiar with journal,  Unbiased and ethical professionals,  Self-critical professionals,  Reliable professionals,  Skilled critics,  Respectful communicators,  Advocates,  Advisors | 11, 13, 14, 15, 17, 31, 33, 35, 36, 41, 42, 44, 45, 62, 68, 71 | 16 |
| Allen, T. W.(3) | USA | English | Bariatric Surgical Practice and Patient Care | 2014 | Editorial | Dutiful/altruistic towards scientific community, Unbiased and ethical professionals, Reliable professionals,  Skilled critics,  Respectful communicators,  Advocates,  Reviewers should not | 7, 14, 15, 16, 18, 35, 44, 46, 61, 62, 67, 73 | 12 |
| Andersson, K.-E.(4) | Denmark | English | The Journal of Urology | 2011 | Editorial | Dutiful/altruistic towards scientific community,  Self-critical professionals,  Skilled critics,  Respectful communicators,  Advisors | 4, 31, 42, 57, 71 | 5 |
| Bacchetti, P.(5) | USA | English | BMJ | 2002 | Editorial | Self-critical professionals | 32 | 1 |
| Baker, D. J.(6) | USA | English | Association of periOperative Registered Nurses Journal | 2015 | Editorial | Reliable professionals, Skilled critics, Respectful communicators,  Advisors,  Reviewers should not | 35, 38, 42, 44, 45, 60, 61, 71, 73 | 9 |
| Bernstein, J.(7) | USA | English | Clinical Orthopaedics and Related Research | 2013 | Commentary | Unbiased and ethical professionals | 16 | 1 |
| Berquist, T.(8) | USA | English | American Journal of Roentgenology | 2014 | Editorial | Unbiased and ethical professionals | 23, 27 | 2 |
| Brandon, D.(9) | USA | English | Advances in Neonatal Care | 2015 | Editorial | Proficient experts in their field,  Unbiased and ethical professionals,  Reliable professionals,  Skilled critics | 1, 13, 14, 35, 43 | 5 |
| Brock, W. J.(10) | USA | English | International Journal of Toxicology | 2014 | Editorial | Unbiased and ethical professionals,  Self-critical professionals,  Reliable professionals | 13, 31, 36 | 3 |
| Byrne, R.(11) | N/A | English | EuroIntervention | 2016 | Editorial | Skilled critics,  Respectful communicators,  Advisors | 43, 45, 46, 57, 71 | 5 |
| Campion, E. W.(12) | N/A | English | New England Journal of Medicine | 2000 | Editorial | Unbiased and ethical professionals,  Advisors,  Reviewers should not | 19, 70, 72 | 3 |
| Carrió, I.(13) | Spain | English | European Journal of Nuclear Medicine and Molecular Imaging | 2006 | Editorial | Dutiful/altruistic towards scientific community,  Skilled critics | 4, 43 | 2 |
| Chew, F.(14) | USA | English | American Journal of Radiology | 1992 | Editorial | Proficient experts in their field, Familiar with journal,  Respectful communicators,  Reviewers should not | 1, 11, 61, 72, 73 | 5 |
| Christensen, N. B.(15) | N/A | English | International Journal of  Urology | 2010 | Editorial | Dutiful/altruistic towards scientific community,  Unbiased and ethical professionals,  Skilled critics,  Advisors | 5, 14, 15, 42, 51, 71 | 6 |
| Clark, R.(16) | UK | English | British Dental Journal | 2012 | Editorial | Unbiased and ethical professionals | 17 | 1 |
| Clarke, S. P.(17) | Canada | English | Canadian Journal of Nursing Research | 2006 | Editorial | Proficient experts in their field,  Skilled critics,  Gatekeepers,  Educators,  Advocates | 1, 44, 46, 64, 65, 67 | 6 |
| Cotton, P.(18) | USA | English | JAMA | 1994 | Editorial | N/A | N/A | 0 |
| Cowell, J. M.(19) | USA | English | The Journal of School Nursing | 2014 | Editorial | N/A | N/A | 0 |
| Cowell, J. M.(20) | USA | English | The Journal of School Nursing | 2015 | Editorial | Familiar with journal,  Skilled critics,  Reviewers should not | 11, 46, 49, 73 | 4 |
| Crawford, S.(21) | USA | English | Bulletin of the Medical Library Association | 1988 | Editorial | N/A | N/A | 0 |
| Cummings, P.(22) | N/A | English | Archives of Pediatrics & Adolescent Medicine | 2002 | Editorial | Unbiased and ethical professionals,  Skilled critics,  Reliable professionals,  Respectful communicators,  Advisors, Reviewers should not | 13, 40, 43, 46, 57, 58, 62, 70, 72 | 9 |
| Cusik, A.(23) | Australia | English | Australian Occupational Therapy Journal | 2016 | Editorial | Proficient experts in their field,  Unbiased and ethical professionals,  Gatekeepers,  Advisors | 1, 13, 63, 70 | 4 |
| da Cruz, I. C.(24) | Brazil | Portuguese | Online Brazilian Journal of Nursing | 2008 | Editorial | N/A | N/A | 0 |
| David, B.(25) | UK | English | European Journal of Neuroscience | 2016 | Editorial | Skilled critics, Reviewers should not | 43, 74 | 2 |
| de Araújo, C.(26) | Brazil | English | Arquivos Brasileros de Cardiololgia | 2012 | Perspective | Dutiful/altruistic towards scientific community,  Familiar with journal,  Unbiased and ethical professionals,  Self-critical professionals,  Reliable professionals, Self-critical professionals,  Skilled critics,  Advisors,  Reviewers should not | 7, 11, 14, 31, 32, 33, 35, 36, 38, 39, 42, 70, 71, 72, 75 | 15 |
| de Hon, F.(27) | Netherlands | English | Vaccine | 2013 | Editorial | Unbiased and ethical professionals,  Reliable professionals,  Skilled critics | 16, 35, 42, 43, 44, 51 | 6 |
| DeBarr, K.(28) | USA | English | Californian Journal of Health Promotion | 2007 | Editorial | Familiar with journal,  Unbiased and ethical professionals,  Self-critical professionals,  Skilled critics,  Reliable professionals,  Respectful communicators | 11, 13, 31, 34, 36, 42, 44, 47, 53, 57, 61 | 11 |
| Del Mar, C.(29) | Australia | English | BMC Medicine | 2015 | Tutorial | Unbiased and ethical professionals  Reliable professionals,  Skilled critics  Respectful communicators,  Advisors | 13, 17, 35, 42, 43, 51, 60, 71 | 8 |
| Dinis-Ribeiro, M.(30) | Portugal | English | Endoscopy | 2013 | Editorial | Proficient experts in their field,  Familiar with journal,  Unbiased and ethical professionals,  Reliable professionals,  Skilled critics | 1, 11, 13, 14, 15, 16, 23, 35, 42, 43 | 10 |
| Do Vale, E. S.(31) | Brazil | Portuguese | Anais Brasileiros de Dermatologia | 2008 | Editorial | Unbiased and ethical professionals,  Self-critical professionals,  Reliable professionals,  Skilled critics,  Respectful communicators | 13, 17, 18, 21, 24, 28, 31, 32, 33, 35, 37, 38, 39, 42, 48, 57 | 16 |
| Donato, H.(32) | Portugal | English | Acta Médica Portuguesa | 2012 | Editorial | Reliable professionals,  Skilled critics,  Advisors | 35, 43, 70 | 3 |
| Drummon, A.(33) | UK | English | British Journal of Occupational Therapy | 1996 | Editorial | Familiar with journal, Unbiased and ethical professionals,  Self-critical professionals,  Skilled critics,  Educators,  Reviewers should not | 11, 23, 32, 43, 48, 66, 75 | 7 |
| Dutta, M. J.(34) | USA | English | Health Communication | 2009 | Commentary | Proficient experts in their field,  Unbiased and ethical professionals,  Reliable professionals,  Skilled critics, Respectful communicators,  Educators | 1, 17, 35, 46, 56, 58, 66 | 7 |
| el-Azhary, R.(35) | N/A | English | International Journal of Dermatology | 2016 | Editorial | Unbiased and ethical professionals,  Skilled critics | 19, 43 | 2 |
| Emanuel, L.(36) | USA | English | Archives of Internal Medicine | 2005 | Editorial | Proficient experts in their field,  Familiar with journal, Unbiased and ethical professionals | 1, 11, 13, 15, 16 | 5 |
| Emden, C.(37) | Australia | English | Nursing Inquiry | 1996 | Editorial | Dutiful/altruistic towards scientific community,  Familiar with journal,  Unbiased and ethical professionals,  Self-critical professionals,  Reliable professionals,  Skilled critics,  Respectful communicators | 9, 11, 15, 34, 35, 40, 43, 49, 61 | 9 |
| Faggion, C. M.(38) | UK | English | British Dental Journal | 2016 | Editorial | Reviewers should not | 74 | 1 |
| Fain, J. A.(39) | USA | English | The Diabetes EDUCATOR | 2011 | Editorial | N/A | N/A | 0 |
| Feinstein, A.(40) | USA | English | Journal of Clinical Epidemiology | 1989 | Editorial | Proficient experts in their field, Reliable professionals, Skilled critics,  Gatekeepers,  Educators | 1, 37, 42, 43, 64, 65 | 6 |
| Feldstein Ewing, S.(41) | USA | English | Evidence Based Medicine | 2015 | Perspective | Familiar with journal,  Reliable professionals,  Skilled critics,  Respectful communicators | 11, 35, 43, 46, 48, 49, 57 | 7 |
| Feldman, M. D.(42) | USA | English | Journal of Geneneral Internal Medicine | 2016 | Editorial | Skilled critics | 43 | 1 |
| Ferris, L.(43) | Canada | English | Journal of Child Neurology | 2010 | Editorial | Proficient experts in their field,  Unbiased and ethical professionals,  Reliable professionals,  Skilled critics,  Respectful communicators | 1, 13, 15, 17, 35, 37, 44, 45, 48, 53, 61 | 11 |
| Fisher, R.(44) | USA | English | Epilepsia | 2004 | Editorial | Unbiased and ethical professionals,  Advisors | 13, 71 | 2 |
| Fitzpatrick, J.(45) | N/A | English | Applied Nursing Research | 2017 | Editorial | Skilled critics, Reviewers should not | 42, 43, 45, 73 | 4 |
| Flood, A.(46) | N/A | English | Health Services Research | 2004 | Editorial | Dutiful/altruistic towards scientific community,  Unbiased and ethical professionals,  Skilled critics | 6, 17, 43 | 3 |
| Fontes, B. M.(47) | Brazil | English | Arquivos Brasileiros de Oftalmologia | 2015 | Editorial | Proficient experts in their field,  Skilled critics,  Advisors | 1, 2, 43, 45, 70 | 5 |
| Foster, R.(48) | N/A | English | Journal for Specialists in Pediatric Nursing | 2008 | Editorial | Dutiful/altruistic towards scientific community,  Familiar with journal,  Unbiased and ethical professionals,  Reliable professionals,  Advisors | 4, 7, 11, 13, 14, 37, 71 | 7 |
| Friedman, D.(49) | USA | English | American Journal of Roentgenology | 1995 | Perspective | Reliable professionals,  Skilled critics | 35, 43 | 2 |
| Furness, P.(50) | UK | English | BMJ | 1994 | Commentary | Proficient experts in their field,  Skilled critics | 1, 47 | 2 |
| Gennaro, S.(51) | N/A | English | Journal of Nursing Scholarship | 2015 | Editorial | N/A | N/A | 0 |
| Gitanjali, B.(52) | India | English | Journal of Postgraduate Medicine | 2001 | Editorial | Proficient experts in their field,  Unbiased and ethical professionals,  Self-critical professionals,  Reliable professionals,  Skilled critics,  Respectful communicators,  Reviewers should not | 1, 13, 14, 17, 20, 21, 22, 29, 31, 33, 36, 37, 38, 43, 45, 57,  72 | 17 |
| Giunta, R. E.(53) | Germany | English | Handchirurgie Mikrochirurgie Plastische Chirurgie | 2012 | Editorial | Unbiased and ethical professionals, Skilled critics,  Respectful communicators,  Advisors | 16, 42, 43, 44, 58, 70 | 6 |
| Glick, M.(54) | N/A | English | Journal of the American Dental Association | 2007 | Editorial | Proficient experts in their field,  Skilled critics,  Respectful communicators,  Advisors | 1, 42, 61, 71 | 4 |
| Goldbeck-Wood, S.(55) | UK | English | BMJ | 1998 | Editorial | Reliable professionals,  Skilled critics,  Respectful communicators,  Reviewers should not | 35, 42, 43, 44, 45, 46, 47, 59, 65, 72 | 10 |
| Goodlett, C.(56) | USA | English | Alcohol | 2005 | Editorial | Unbiased and ethical professionals,  Reliable professionals,  Skilled critics,  Respectful communicators | 16, 35, 37, 38, 41, 42, 57 | 7 |
| Gough, N. R.(57) | USA | English | Science Signaling | 2009 | Editorial | Unbiased and ethical professionals,  Self-critical professionals,  Reliable professionals,  Skilled critics,  Respectful communicators,  Gatekeepers | 15, 32, 35, 40, 42, 43, 49, 51, 52, 58, 60, 64 | 12 |
| Halder, N.(58) | UK | English | Advances in Psychiatric Treatment | 2011 | Editorial | Familiar with journal,  Unbiased and ethical professionals,  Self-critical professionals  Reliable professionals,  Skilled critics,  Respectful communicators | 11, 13, 16, 31, 32, 35, 36, 47, 50, 57 | 10 |
| Harms, M.(59) | USA | English | Physiotherapy | 2006 | Editorial | Proficient experts in their field,  Skilled critics,  Respectful communicators | 1, 42, 43, 57 | 4 |
| Heddle, N.(60) | Canada | English | Transfusion | 2009 | Commentary | Proficient experts in their field,  Unbiased and ethical professionals,  Self-critical professionals,  Reliable professionals,  Skilled critics,  Respectful communicators,  Gatekeepers,  Advocates | 1, 13, 14, 16, 18, 24, 31, 34, 35, 43, 45, 58, 59, 64, 67 | 15 |
| Helton, M.(61) | USA | English | The Journal of Paediatrics | 2011 | Commentary | Unbiased and ethical professionals,  Skilled critics,  Advisors | 13, 14, 15, 16, 20, 52, 71 | 7 |
| Hernandez, L. V.(62) | USA | English | Gastrointestinal Endoscopy | 2009 | Commentary | Familiar with journal,  Self-critical professionals | 11, 34 | 2 |
| Hoyt, K. S.(63) | USA | English | Advanced Emergency Nursing Journal | 2007 | Editorial | Unbiased and ethical professionals,  Reliable professionals,  Skilled critics,  Respectful communicators,  Gatekeepers | 13, 14, 17, 19, 20, 24, 37, 42, 46, 48, 57, 63 | 12 |
| Isaacs, D.(64) | Australia | English | Journal of Paediatrics and Child Health | 2004 | Commentary | Familiar with journal,  Self-critical professionals,  Reliable professionals,  Respectful communicators,  Reviewers should not | 11, 32, 35, 57, 72, 73, 75 | 7 |
| Izumi, S.(65) | USA | English | Japan Journal of Nursing Science | 2009 | Editorial | Skilled critics, Gatekeepers,  Advisors | 43, 57, 64, 70 | 4 |
| Jacobson, R.(66) | USA | English | Academic Paediatrics | 2017 | Commentary | Proficient experts in their field,  Unbiased and ethical professionals,  Self-critical professionals,  Reliable professionals,  Skilled critics,  Respectful communicators,  Gatekeepers,  Educators,  Advisors | 1, 14, 15, 16, 20, 32, 35, 39, 41, 42, 43, 45, 46, 50, 53, 57, 60, 64, 65, 70 | 20 |
| Jain, A. K.(67) | India | English | Indian Journal of Orthopaedics | 2009 | Editorial | Unbiased and ethical professionals,  Self-critical professionals,  Reliable professionals,  Skilled critics,  Respectful communicators,  Advisors | 13, 14, 18, 19, 21, 31, 35, 42, 45, 46, 47, 57, 58, 71 | 14 |
| Kasiske, B. L.(68) | N/A | English | American Journal of Kidney Diseases | 2005 | Editorial | Skilled critics | 42, 47 | 2 |
| Katz, A.(69) | N/A | English | Oncology Nursing Forum | 2016 | Editorial | Reliable professionals,  Reviewers should not | 35, 73 | 2 |
| Kearney, M.(70) | UK | English | Research in Nursing & Health | 2016 | Editorial | Proficient experts in their field | 1, 2 | 2 |
| Kehrer, J. P.(71) | Canada | English | Toxicology | 2013 | Letter | Unbiased and ethical professionals,  Reliable professionals,  Skilled critics,  Advisors | 15, 16, 35, 44, 70 | 5 |
| Kotsis, S.(72) | N/A | English | Plastic and Reconstructive Surgery | 2014 | Editorial | Proficient experts in their field,  Unbiased and ethical professionals,  Self-critical professionals,  Reliable professionals,  Skilled critics,  Respectful communicators,  Educators,  Advisors | 1, 13, 31, 33, 36, 42, 43, 45, 46, 57, 62, 66, 70 | 13 |
| Kottner, J.(73) | Germany | English | International Journal of Nursing Studies | 2016 | Editorial | Proficient experts in their field,  Unbiased and ethical professionals,  Reliable professionals,  Reviewers should not | 1, 13, 35, 37, 39, 72 | 6 |
| Krome, R. L.(74) | N/A | English | Annals of Emergency Medicine | 1983 | Editorial | Unbiased and ethical professionals,  Advocates | 15, 69 | 2 |
| Landauer, A.(75) | Australia | English | Australian Drug and Alcohol Review | 1987 | Editorial | Proficient experts in their field,  Familiar with journal, Unbiased and ethical professionals,  Reliable professionals,  Reviewers should not | 1, 11, 23, 35, 74, 75 | 6 |
| Lau, D.(76) | N/A | English | Canadian Journal of Diabetes | 2016 | Editorial | Proficient experts in their field, | 1, 2 | 2 |
| Lichter, P. R.(77) | N/A | English | Ophthalmology | 1993 | Editorial | Proficient experts in their field,  Unbiased and ethical professionals,  Self-critical professionals,  Reliable professionals,  Skilled critics,  Advocates  Reviewers should not | 1, 14, 15, 22, 31, 36, 42, 43, 44, 45, 68, 69, 71, 72 | 14 |
| Lowe, N.(78) | N/A | English | Journal of Obstetric, Gynecologic, & Neonatal Nursing | 2007 | Editorial | Proficient experts in their field,  Familiar with journal,  Unbiased and ethical professionals,  Reliable professionals,  Skilled critics | 1, 2, 11, 17, 35, 37, 42, 44, 45 | 9 |
| Luscher, T.(79) | USA | English | Journal of the American College of Cardiology | 2016 | Letter | Unbiased and ethical professionals,  Self-critical professionals,  Skilled critics | 13, 16, 17, 31, 42, 43 | 6 |
| Mackenzie, L.(80) | Australia | English | Australian Occupational Therapy Journal | 2006 | Editorial | Skilled critics, Educators | 42, 54, 66 | 3 |
| Mannis, M. J.(81) | N/A | English | Cornea | 1997 | Editorial | Unbiased and ethical professionals,  Respectful communicators,  Reviewers should not | 16, 17, 57, 74 | 4 |
| Manske, P.(82) | N/A | English | The Journal of Hand Surgery | 1997 | Commentary | Skilled critics, Reviewers should not | 43, 72 | 2 |
| Martin-Sanchez, F.(83) | Spain | English | Emergencias | 2012 | Editorial | Proficient experts in their field,  Dutiful/altruistic towards scientific community,  Self-critical professionals,  Unbiased and ethical professionals,  Reliable professionals,  Skilled critics | 1, 2, 4, 8, 15, 33, 35, 36, 45, 49, 56 | 11 |
| Marušić, M.(84) | Croatia | English | Croatian Medical Journal | 2005 | Editorial | Proficient experts in their field,  Dutiful/altruistic towards scientific community,  Familiar with journal,  Unbiased and ethical professionals,  Reliable professionals,  Skilled critics,  Educators | 1, 4, 11, 15, 35, 36, 43, 65 | 8 |
| Merell, R.(85) | N/A | English | Telemedicine and E-Health | 2007 | Editorial | Unbiased and ethical professionals,  Reliable professionals,  Skilled critics,  Gatekeepers | 14, 35, 42, 63 | 4 |
| Minion, D.(86) | N/A | English | Journal of Vascular Surgery | 2007 | Editorial | Proficient experts in their field,  Unbiased and ethical professionals,  Reliable professionals | 1, 14, 17, 20, 35 | 5 |
| Moher, D.(87) | Canada | English | BMC Medicine | 2015 | Tutorial | Skilled critics,  Respectful communicators | 42, 57 | 2 |
| Moher, D.(88) | Canada | English | Peer review in Health Sciences | 2003 | Book chapter | Proficient experts in their field,  Familiar with journal,  Unbiased and ethical professionals,  Self-critical professionals,  Reliable professionals,  Skilled critics,  Respectful communicators,  Advisors,  Reviewers should not | 1, 3, 11, 13, 14, 18, 20, 21, 31, 32, 34, 35, 36, 37, 39, 40, 42, 43, 47, 50, 58, 70, 71, 73 | 24 |
| Moore, K. N.(89) | N/A | English | Journal of Wound, Ostomy, & Continence Nursing | 2005 | Editorial | Proficient experts in their field, Dutiful/altruistic towards scientific community,  Unbiased and ethical professionals, | 1, 4, 14, 18, | 4 |
| Morse, J. M.(90) | USA | English | Qualitative Health Research | 2014 | Editorial | Skilled critics | 42 | 1 |
| Muir-Cochrane, E.(91) | N/A | English | Nursing & Health Sciences | 2013 | Editorial | Unbiased and ethical professionals,  Skilled critics,  Respectful communicators | 13, 14, 28, 42, 44, 49, 52, 54, 57 | 9 |
| Nelson, C. A.(92) | USA | English | Dermatologic Clinics | 2009 | Editorial | Proficient experts in their field,  Unbiased and ethical professionals  Reliable professionals,  Skilled critics,  Respectful communicators,  Gatekeepers,  Reviewers should not | 3, 13, 14, 15, 35, 36, 42, 58, 63, 72 | 10 |
| Newell, F. W.(93) | USA | English | American Journal of Ophthalmology | 1990 | Editorial | N/A | N/A | 0 |
| Nexoe, J.(94) | Denmark | English | Scandinavian Journal of Primary Health Care | 2014 | Editorial | Reliable professionals, | 35, 36, 38 | 3 |
| Olson, C. M.(95) | N/A | English | American Journal of Emergency Medicine | 1990 | Editorial | Skilled critics, Advisors | 43, 70 | 2 |
| Oman, K.(96) | USA | English | Journal of Emergency Nursing | 2009 | Editorial | Unbiased and ethical professionals,  Skilled critics,  Respectful communicators,  Advisors | 13, 42, 48, 57, 58, 70, 71 | 7 |
| Pearson, G. S.(97) | USA | English | Journal of the American Psychiatric Nurses Association | 2016 | Editorial | Proficient experts in their field,  Reliable professionals,  Skilled critics,  Respectful communicators | 1, 35, 38, 39, 42, 43,  57, 58 | 8 |
| Peh, W.C.G.(98) | Singapore | English | Singapore Medical Journal | 2009 | Editorial | Proficient experts in their field,  Unbiased and ethical professionals,  Skilled critics,  Respectful communicators,  Gatekeepers | 1, 13, 14, 15, 16, 17, 18, 24, 42, 43, 54, 57, 63 | 13 |
| Perkins, D.(99) | Australia | English | Australian Journal of Rural Health | 2009 | Editorial | Proficient experts in their field,  Reliable professionals,  Skilled critics,  Gatekeepers,  Educators,  Advisors | 1, 2, 35, 43, 49, 63, 65, 70 | 8 |
| Pierson, C. A.(100) | N/A | English | Journal of the American Association of Nurse Practitioners | 2014 | Editorial | N/A | N/A | 0 |
| Pierson, C. A.(101) | N/A | English | Journal of the American Association of Nurse Practitioners | 2015 | Editorial | Unbiased and ethical professionals,  Gatekeepers,  Advisors | 13, 63, 70 | 3 |
| Pierson, C. A.(102) | N/A | English | Journal of the American Association of Nurse Practitioners | 2016 | Editorial | Skilled critics,  Advisors | 42, 70 | 2 |
| Pietrzak, W. S.(103) | N/A | English | Journal of Craniofacial Surgery | 2010 | Editorial | Proficient experts in their field,  Dutiful/altruistic towards scientific community,  Unbiased and ethical professionals,  Reliable professionals,  Gatekeepers,  Advisors,  Reviewers should not | 1, 8, 14, 35, 37, 41, 64, 70, 71, 72, 73, 74 | 12 |
| Polak, J. F.(104) | USA | English | American Journal of Roentgenology | 1995 | Perspective | Proficient experts in their field,  Unbiased and ethical professionals,  Skilled critics,  Reviewers should not | 1, 26, 43, 46, 48, 50, 54, 72, 73 | 9 |
| Poland, G. A.(105) | USA | English | Vaccine | 2013 | Editorial | Proficient experts in their field,  Unbiased and ethical professionals,  Self-critical professionals,  Reliable professionals  Advisors | 1, 13, 33, 35, 70 | 5 |
| Prado, A.(106) | N/A | English | Plastic and Reconstructive Surgery | 2009 | Editorial | Familiar with journal,  Reliable professionals,  Skilled critics,  Respectful communicators,  Gatekeepers,  Educators | 11, 35, 43, 45, 49, 59, 64, 65 | 8 |
| Pyne, D.(107) | N/A | English | International Journal of Sports Physiology and Performance | 2011 | Editorial | Reliable professionals,  Skilled critics,  Advisors,  Reviewers should not | 36, 41, 42, 46, 71, 73, 74 | 7 |
| Quan, S. F.(108) | USA | English | Journal of Clinical Sleep Medicine | 2014 | Editorial | Reliable professionals,  Advisors | 35, 36, 70 | 3 |
| Raff, H.(109) | USA | English | Advances in Physiology Education | 2013 | Editorial | Unbiased and ethical professionals,  Respectful communicators | 17, 58 | 2 |
| Ramsden, V. R.(110) | Canada | English | Canadian Family Physician | 2014 | Editorial | Proficient experts in their field,  Unbiased and ethical professionals,  Self-critical professionals,  Reliable professionals,  Skilled critics,  Respectful communicators,  Educators | 1, 13, 14, 15, 19, 31, 35, 38, 39, 40, 42, 46, 49, 57, 65 | 15 |
| Relman, A. S.(111) | USA | English | Western Journal of Medicine | 1990 | Commentary | Advisors,  Reviewers should not | 70, 72 | 2 |
| Rennie, D.(112) | USA | English | American Journal of Diseases of Children | 1988 | Editorial | Proficient experts in their field,  Unbiased and ethical professionals,  Reliable professionals,  Skilled critics | 1, 14, 17, 35, 43 | 5 |
| Resnick, B.(113) | USA | English | Geriatric Nursing | 2010 | Editorial | Dutiful/altruistic towards scientific community | 4, 5 | 2 |
| Reyes B, H.(114) | N/A | English | Revista Medica De Chile | 2013 | Editorial | Unbiased and ethical professionals,  Reliable professionals,  Skilled critics, | 13, 19, 35, 38, 42, | 6 |
| Richardson, D.(115) | N/A | English | JAVA - Journal of the Association for Vascular Access | 2006 | Editorial | Unbiased and ethical professionals,  Reliable professionals,  Skilled critics,  Respectful communicators | 13, 14, 35, 38, 42, 57 | 5 |
| Riss, P.(116) | N/A | English | International Urogynecology Journal | 2012 | Editorial | Unbiased and ethical professionals,  Reliable professionals,  Skilled critics,  Respectful communicators,  Advisors | 13, 37, 43, 51, 57, 71 | 6 |
| Roberts, J.(117) | N/A | English | Journal of Sexual Medicine | 2008 | Editorial | Skilled critics, Gatekeepers,  Reviewers should not | 43, 45, 64, 73 | 4 |
| Roberts, L. W.(118) | N/A | English | Academic Psychiatry Win | 2002 | Editorial | Proficient experts in their field,  Unbiased and ethical professionals,  Self-critical professionals,  Reliable professionals,  Skilled critics,  Respectful communicators,  Educators,  Advisors,  Reviewers should not | 1, 13, 14, 17, 19, 22, 31, 35, 42, 60, 62, 65, 66, 70, 75 | 15 |
| Rogers, L. F.(119) | USA | English | American Journal of Roentgenology | 2002 | Editorial | Skilled critics,  Reviewers should not | 43, 73 | 2 |
| Sadun, A.(120) | N/A | English | Ophthalmology | 2002 | Editorial | Proficient experts in their field,  Skilled critics,  Respectful communicators,  Advisors,  Reviewers should not | 1, 42, 47, 59, 70, 75 | 6 |
| Salasche, S. J.(121) | N/A | English | Dermatologic Surgery | 1997 | Editorial | Proficient experts in their field,  Unbiased and ethical professionals,  Reliable professionals,  Skilled critics | 1, 13, 16, 17, 26, 37, 43, 44 | 8 |
| Saper, C. B.(122) | N/A | English | Annals of Neurology | 2014 | Editorial | Reliable professionals,  Skilled critics,  Respectful communicators | 36, 37, 42, 50, 60 | 5 |
| Sciortino, J. E.(123) | N/A | English | Cuaj-Canadian Urological Association Journal | 2013 | Editorial | Unbiased and ethical professionals,  Self-critical professionals,  Reliable professionals,  Skilled critics,  Respectful communicators,  Gatekeepers | 13, 14, 15, 16, 27, 31, 35, 36, 42, 58, 63 | 11 |
| Sellke, F. W.(124) | N/A | English | Journal of Thoracic and Cardiovascular Surgery | 2003 | Editorial | Proficient experts in their field,  Unbiased and ethical professionals,  Skilled critics,  Respectful communicators | 1, 17, 42, 47, 57 | 5 |
| Sohail, S.(125) | N/A | English | Journal of the College of Physicians and Surgeons Pakistan | 2015 | Editorial | Proficient experts in their field,  Unbiased and ethical professionals,  Reliable professionals,  Skilled critics | 1, 13, 36, 37, 43, 45 | 6 |
| Spolarich, A. E.(126) | USA | English | Canadian Journal of Dental Hygiene | 2014 | Editorial | Proficient experts in their field,  Dutiful/altruistic towards scientific community,  Familiar with journal,  Reliable professionals,  Skilled critics,  Educators,  Advisors,  Reviewers should not | 1, 4, 5, 11, 35, 38, 39,  42, 43, 44, 45, 47, 51, 53, 65, 70, 71, 74, 76 | 19 |
| Stein, K. F.(127) | USA | English | Journal of the American Psychiatric Nurses Association | 2014 | Editorial | Proficient experts in their field,  Unbiased and ethical professionals,  Self-critical professionals,  Reliable professionals,  Skilled critics | 1, 13, 14, 16, 17, 18, 31, 37, 42, 43, 44 | 11 |
| Swartz, M. K.(128) | N/A | English | Journal of Pediatric Health Care | 2008 | Editorial | Dutiful/altruistic towards scientific community,  Familiar with journal,  Unbiased and ethical professionals,  Self-critical professionals,  Reliable professionals,  Skilled critics,  Respectful communicators,  Gatekeepers,  Educators, | 4, 5, 12, 13, 14, 19, 25, 27, 31, 35, 42, 43, 44, 45, 48, 49, 62, 63, 65, 66 | 20 |
| Taylor, F. R.(129) | N/A | English | Headache | 2009 | Editorial | Dutiful/altruistic towards scientific community,  Familiar with journal,  Unbiased and ethical professionals,  Self-critical professionals  Reliable professionals,  Skilled critics | 4, 11, 13, 15, 23, 26, 31, 32, 36, 37, 52 | 11 |
| Thombs, B. D.(130) | Canada | English | Canadian Medical Association Journal | 2012 | Editorial | Unbiased and ethical professionals,  Skilled critics,  Advisors | 13, 28, 43, 70 | 4 |
| Twentyman, P.(131) | UK | English | British Journal of Cancer | 1991 | Editorial | Unbiased and ethical professionals,  Advisors | 14, 17, 18, 22, 71 | 4 |
| Van Norman, G. A.(132) | N/A | English | Clinical Ethics in Anesthesiology: A Case-Based Textbook | 2011 | Book chapter | Proficient experts in their field,  Unbiased and ethical professionals,  Skilled critics,  Respectful communicators | 1, 13, 14, 15, 25, 52, 62 | 7 |
| Vintzileos, A. M.(133) | USA | English | Journal of Ultrasound in Medicine | 2010 | Editorial | Proficient experts in their field,  Unbiased and ethical professionals,  Reliable professionals,  Skilled critics | 13, 14, 35, 36, 42, 43, 44 | 7 |
| Wagner, P.D.(134) | USA | English | Journal of Applied Physiology | 2016 | Editorial | Unbiased and ethical professionals,  Reliable professionals,  Skilled critics,  Respectful communicators,  Advisors,  Reviewers should not | 13, 14, 18, 20, 21, 27, 35, 42, 47, 60,  71, 74 | 12 |
| Wick, G.(135) | Austria | English | International Archives of Allergy and Immunology | 1996 | Editorial | Unbiased and ethical professionals,  Skilled critics,  Respectful communicators | 15, 42, 43, 59, 60 | 5 |
| Wierzbinski-Cross, H.(136) | N/A | English | Journal for Nurses in Professional Development | 2017 | Editorial | Dutiful/altruistic towards scientific community,  Familiar with journal,  Unbiased and ethical professionals,  Self-critical professionals  Reliable professionals,  Skilled critics,  Advisors | 4, 11, 13, 14, 16, 19, 31, 35, 36, 43, 52, 71 | 12 |
| Wilder, R. S.(137) | N/A | English | Journal of Dental Hygiene | 2014 | Editorial | Proficient experts in their field,  Reliable professionals,  Respectful communicators | 1, 2, 35, 38,  57, 58 | 6 |
| Wolf, L. A.(138) | N/A | English | Journal of Emergency Nursing | 2016 | Editorial | Proficient experts in their field,  Skilled critics | 1, 3, 43 | 3 |
| Young, S. N.(139) | Canada | English | Journal of Psychiatry & Neuroscience | 2003 | Editorial | Proficient experts in their field,  Reliable professionals,  Respectful communicators,  Reviewers should not | 1, 37, 61, 72 | 4 |
| Zinsky, R.(140) | Germany | English | Breathe | 2014 | Editorial | Self-critical professionals,  Reliable professionals,  Skilled critics | 31, 37, 43 | 3 |
| **Total** |  |  |  |  |  |  |  | 884 |

^B^ Corresponds to item number from the list of roles (Table 3)

^C^ Number of extracted roles statements

**Table 2** Tasks identified in book chapters, commentaries, editorials, letters, perspectives, and tutorials

| **Author** | **Country** | **Language** | **Journal** | **Year** | **Design** | **Theme(s)** | **Tasks** | **# of tasks** |
| --- | --- | --- | --- | --- | --- | --- | --- | --- |
| Alam, S.(1) | UK | English | BMC Medicine | 2015 | Editorial | Organization and approach to review,  Methods,  Discussion/Conclusion | 2, 6, 30, 38, 42, 50 | 6 |
| Allen, T. W.(2) | USA | English | The Journal of the American Osteopathic Association | 2013 | Commentary | Organization and approach to review,  Make general comments, References,  Address ethical aspects,  Assess manuscript presentation,  Provide recommendations | 3, 11, 12, 13, 14, 17, 18, 56, 57, 62, 64, 71 | 12 |
| Allen, T. W.(3) | USA | English | Bariatric Surgical Practice and Patient Care | 2014 | Editorial | Organization and approach to review, Results,  Discussion/Conclusion | 9, 46, 50 | 3 |
| Andersson, K.-E.(4) | Denmark | English | The Journal of Urology | 2011 | Editorial | Make general comments,  Title is accurate,  Abstract, Introduction, Methods,  Results,  Discussion/  Conclusion, References | 17, 18, 21, 22, 23, 26, 28, 31, 33, 36, 41, 45, 46, 47, 49, 50, 51, 54, 56 | 19 |
| Bacchetti, P.(5) | USA | English | BMJ | 2002 | Editorial | Organization and approach to review, Methods,  Discussion/Conclusion | 2, 34, 50 | 3 |
| Baker, D. J.(6) | USA | English | Association of periOperative Registered Nurses Journal | 2015 | Editorial | Organization and approach to review, Make general comments,  Provide recommendations | 5, 11, 14, 17, 70, 71, 72 | 7 |
| Bernstein, J.(7) | USA | English | Clinical Orthopaedics and Related Research | 2013 | Commentary | Make general comments,  Results,  Discussion/ConclusionReferences | 11, 46, 50, 56 | 4 |
| Berquist, T.(8) | USA | English | American Journal of Roentgenology | 2014 | Editorial | N/A | N/A | 0 |
| Brandon, D.(9) | USA | English | Advances in Neonatal Care | 2015 | Editorial | Make general comments,  Assess manuscript presentation,  Provide recommendations | 13, 62, 63, 70 | 4 |
| Brock, W. J.(10) | USA | English | International Journal of Toxicology | 2014 | Editorial | Organization and approach to review,  Make general comments, Discussion/Conclusion  Address ethical aspects | 2, 11, 50, 57 | 4 |
| Byrne, R.(11) | N/A | English | EuroIntervention | 2016 | Editorial | Organization and approach to review,  Make general comments,  Provide recommendations | 2, 3, 10, 11, 12, 13, 17, 70 | 8 |
| Campion, E. W.(12) | N/A | English | New England Journal of Medicine | 2000 | Editorial | Make general comments,  Provide recommendations | 11, 13, 15, 18, 70 | 5 |
| Carrió, I.(13) | Spain | English | European Journal of Nuclear Medicine and Molecular Imaging | 2006 | Editorial | Organization and approach to review, Make general comments,  Provide recommendations | 1, 11, 15, 70 | 4 |
| Chew, F.(14) | USA | English | American Journal of Radiology | 1992 | Editorial | Organization and approach to review,  Make general comments, Introduction, Methods,  Discussion/  Conclusion,  Address ethical aspects,  Provide recommendations | 3, 10, 12, 14, 26, 31, 36, 37, 50, 57, 70 | 11 |
| Christensen, N. B.(15) | N/A | English | International Journal of  Urology | 2010 | Editorial | Make general comments,  Title is accurate, Methods,  Abstract, Introduction, Methods,  Results,  Discussion/ConclusionReferences,  Address ethical aspects | 11, 12, 13, 15, 17, 19, 21, 22, 26, 37, 40, 45, 50, 54, 56, 58, 67 | 17 |
| Clark, R.(16) | UK | English | British Dental Journal | 2012 | Editorial | N/A | N/A | 0 |
| Clarke, S. P.(17) | Canada | English | Canadian Journal of Nursing Research | 2006 | Editorial | Organization and approach to review | 3, 6 | 2 |
| Cotton, P.(18) | USA | English | JAMA | 1994 | Editorial | Organization and approach to review,  Make general comments,  Methods,  Discussion/Conclusion  Assess manuscript presentation | 2, 12, 17, 31, 32, 34, 36, 37, 38, 41, 42, 50, 53, 64 | 14 |
| Cowell, J. M.(19) | USA | English | The Journal of School Nursing | 2014 | Editorial | Make general comments,  Methods,  Assess manuscript presentation | 15, 17, 31, 43, 64 | 5 |
| Cowell, J. M.(20) | USA | English | The Journal of School Nursing | 2015 | Editorial | Organization and approach to review,  Make general comments, Discussion/Conclusion | 3, 14, 51, 53 | 4 |
| Crawford, S.(21) | USA | English | Bulletin of the Medical Library Association | 1988 | Editorial | Make general comments,  Methods, Discussion/ConclusionAssess manuscript presentation,  Provide recommendations | 14, 15, 16, 37, 50, 51, 63, 64, 66, 70, 71 | 11 |
| Cummings, P.(22) | N/A | English | Archives of Pediatrics & Adolescent Medicine | 2002 | Editorial | Organization and approach to review, Make general comments,  Title is accurate, Abstract, Introduction, Methods,  Results, Discussion/Conclusion  References,  Address ethical aspects,  Provide recommendations | 6, 8, 9, 17, 21, 22, 23, 26, 30, 31, 32, 45, 46, 47, 50, 52, 56, 57, 64, 65, 67, 68, 70 | 23 |
| Cusick, A.(23) | Australia | English | Australian Occupational Therapy Journal | 2016 | Editorial | Provide recommendations | 70 | 1 |
| da Cruz, I. C.(24) | Brazil | Portuguese | Online Brazilian Journal of Nursing | 2008 | Editorial | Make general comments,  Title is accurate, Abstract, Introduction, Methods,  Results, Discussion/Conclusion  References,  Address ethical aspects,  Assess manuscript presentation,  Provide recommendations | 11, 12, 13, 15, 16, 17, 21, 22, 26, 28, 30, 33, 45, 50, 51, 55, 56, 57, 63, 64, 67, 72 | 22 |
| David, B.(25) | UK | English | European Journal of Neuroscience | 2016 | Editorial | Organization and approach to review,  Make general comments, Introduction, Methods,  Results, Address ethical aspects, Assess manuscript presentation,  Provide recommendations | 2, 3, 4, 12, 14, 16, 26, 31, 32, 33, 37, 38, 47, 57, 62, 72 | 16 |
| de Araújo, C.(26) | Brazil | English | Arquivos Brasileros de Cardiololgia | 2012 | Perspective | Organization and approach to review,  Make general comments,  Title is accurate, Abstract, Introduction, Methods,  Results,  References,  Assess manuscript presentation,  Provide recommendations | 3, 8, 11, 12, 16, 21, 22, 23, 25, 27, 30, 31, 33, 34, 36, 45, 46, 51, 56, 62, 63, 65, 66, 67, 68, 70, 71, 72 | 28 |
| de Hon, F.(27) | Netherlands | English | Vaccine | 2013 | Editorial | Organization and approach to review,  Make general comments, Introduction, Methods,  Results, Discussion/ConclusionAddress ethical aspects,  Assess manuscript presentation | 1, 12, 13, 14, 16, 26, 33, 45, 50, 57, 62 | 11 |
| DeBarr, K.(28) | USA | English | Californian Journal of Health Promotion | 2007 | Editorial | Make general comments,  Title is accurate, Abstract, Introduction, Methods, Discussion/ConclusionAddress ethical aspects | 15, 21, 22, 23, 26, 28, 31, 32, 34, 36, 37, 43, 50, 58 | 14 |
| Del Mar, C.(29) | Australia | English | BMC Medicine | 2015 | Tutorial | Organization and approach to review,  Make general comments,  Methods, Discussion/ConclusionAddress ethical aspects | 1, 4, 5, 6, 11, 17, 19, 32, 33, 36, 50, 52, 58 | 13 |
| Dinis-Ribeiro, M.(30) | Portugal | English | Endoscopy | 2013 | Editorial | Organization and approach to review, Make general comments,  Abstract, Methods, Results, Discussion/ConclusionAddress ethical aspects,  Assess manuscript presentation,  Provide recommendations | 2, 6, 11, 12, 17, 22, 23, 34, 35, 43, 45, 46, 47, 50, 51, 57, 62, 64, 70, 71 | 20 |
| Do Vale, E. S.(31) | Brazil | Portuguese | Anais Brasileiros de Dermatologia | 2008 | Editorial | Make general comments,  Assess manuscript presentation,  Provide recommendations | 12, 15, 63, 71 | 4 |
| Donato, H.(32) | Portugal | English | Acta Médica Portuguesa | 2012 | Editorial | N/A | N/A | 0 |
| Drummon, A.(33) | UK | English | British Journal of Occupational Therapy | 1996 | Editorial | Title is accurate, Abstract, Introduction, Methods,  Results, Discussion/Conclusion  References,  Assess manuscript presentation,  Provide recommendations | 21, 22, 23, 25, 26, 27, 28, 29, 30, 31, 32, 33, 34, 35, 36, 37, 38, 39, 40, 41, 42, 44, 45, 46, 48, 50, 51, 53, 54, 56, 62, 63, 64, 65, 66, 67, 68, 69, 71 | 39 |
| Dutta, M. J. (34) | USA | English | Health Communication | 2009 | Commentary | N/A | N/A | 0 |
| el-Azhary, R.(35) | N/A | English | International Journal of Dermatology | 2016 | Editorial | Provide recommendations | 70 | 1 |
| Emanuel, L.(36) | USA | English | Archives of Internal Medicine | 2005 | Editorial | Make general comments,  Provide recommendations | 11, 71, 72 | 3 |
| Emden, C.(37) | Australia | English | Nursing Inquiry | 1996 | Editorial | Organization and approach to review | 5 | 1 |
| Faggion, C. M.(38) | UK | English | British Dental Journal | 2016 | Editorial | Make general comments,  Provide recommendations | 11, 15, 71 | 3 |
| Fain, J. A.(39) | USA | English | The Diabetes EDUCATOR | 2011 | Editorial | Make general comments,  Assess manuscript presentation | 13, 15, 16, 18, 62, 64 | 6 |
| Feinstein, A.(40) | USA | English | Journal of Clinical Epidemiology | 1989 | Editorial | N/A | N/A | 0 |
| Feldstein Ewing, S.(41) | USA | English | Evidence Based Medicine | 2015 | Perspective | Organization and approach to review,  Make general comments, Discussion/Conclusion | 7, 17, 50 | 3 |
| Feldman, M. D.(42) | USA | English | Journal of Geneneral Internal Medicine | 2016 | Editorial | Organization and approach to review,  Make general comments,  Provide recommendations | 1, 16, 70 | 3 |
| Ferris, L.(43) | Canada | English | Journal of Child Neurology | 2010 | Editorial | Organization and approach to review, Provide recommendations | 1, 71 | 2 |
| Fisher, R.(44) | USA | English | Epilepsia | 2004 | Editorial | Introduction, Methods,  Results, Discussion/ConclusionReferences,  Assess manuscript presentation,  Provide recommendations | 26, 27, 29, 30, 31, 32, 33, 45, 50, 51, 56, 62, 63, 68, 71 | 15 |
| Fitzpatrick, J.(45) | N/A | English | Applied Nursing Research | 2017 | Editorial | Organization and approach to review,  Make general comments,  Assess manuscript presentation | 2, 11, 15, 62, 64 | 5 |
| Flood, A.(46) | N/A | English | Health Services Research | 2004 | Editorial | Organization and approach to review, Introduction,  Address ethical aspects,  Assess manuscript presentation,  Provide recommendations, Provide recommendations | 2, 4, 26, 27, 57, 62, 63, 70, 71, 72 | 10 |
| Fontes, B. M.(47) | Brazil | English | Arquivos Brasileiros de Oftalmologia | 2015 | Editorial | Organization and approach to review, Methods,  Address ethical aspects | 2, 30, 38, 57 | 4 |
| Foster, R.(48) | N/A | English | Journal for Specialists in Pediatric Nursing | 2008 | Editorial | Address ethical aspects | 57 | 1 |
| Friedman, D.(49) | USA | English | American Journal of Roentgenology | 1995 | Perspective | Organization and approach to review, Make general comments, Discussion/ConclusionAssess manuscript presentation,  Provide recommendations | 3, 11, 50, 69, 71, 72 | 6 |
| Furness, P.(50) | UK | English | BMJ | 1994 | Commentary | N/A | N/A | 0 |
| Gennaro, S.(51) | N/A | English | Journal of Nursing Scholarship | 2015 | Editorial | Make general comments, References,  Assess manuscript presentation | 11, 13, 16, 56, 62, 64 | 6 |
| Gitanjali, B.(52) | India | English | Journal of Postgraduate Medicine | 2001 | Editorial | Address ethical aspects,  Provide recommendations | 57, 59, 70 | 3 |
| Giunta, R. E.(53) | Germany | English | Handchirurgie Mikrochirurgie Plastische Chirurgie | 2012 | Editorial | Organization and approach to review, Make general comments,  Title is accurate, Abstract, Introduction, Methods, Discussion/ConclusionReferences,  Assess manuscript presentation,  Assess manuscript presentation,  Provide recommendations | 1, 12, 21, 24, 26, 27, 29, 30, 46, 51, 56, 62, 63, 65, 70 | 15 |
| Glick, M.(54) | N/A | English | Journal of the American Dental Association | 2007 | Editorial | Organization and approach to review, Methods,  Provide recommendations | 2, 9, 30, 70 | 4 |
| Goldbeck-Wood, S.(55) | UK | English | BMJ | 1998 | Editorial | Make general comments,  Methods, Discussion/ConclusionProvide recommendations | 12, 14, 31, 50, 70 | 5 |
| Goodlett, C.(56) | USA | English | Alcohol | 2005 | Editorial | Make general comments,  Methods,  Results, Discussion/Conclusion | 13, 14, 15, 30, 46, 50 | 6 |
| Gough, N. R.(57) | USA | English | Science Signaling | 2009 | Editorial | Organization and approach to review,  Make general comments,  Methods,  Results, Discussion/ConclusionProvide recommendations | 6, 13, 33, 35, 45, 50, 70 | 7 |
| Halder, N.(58) | UK | English | Advances in Psychiatric Treatment | 2011 | Editorial | Organization and approach to review | 2, 4, 10 | 3 |
| Harms, M.(59) | USA | English | Physiotherapy | 2006 | Editorial | N/A | N/A | 0 |
| Heddle, N.(60) | Canada | English | Transfusion | 2009 | Commentary | Organization and approach to review,  Make general comments, Introduction, Methods,  Results, Discussion/ConclusionReferences,  Assess manuscript presentation,  Provide recommendations | 3, 4, 16, 26, 27, 30, 46, 50, 51, 52, 56, 63, 65, 69, 72 | 15 |
| Helton, M.(61) | USA | English | The Journal of Paediatrics | 2011 | Commentary | Make general comments,  Address ethical aspects,  Provide recommendations | 11, 13, 14, 57, 71 | 5 |
| Hernandez, L. V.(62) | USA | English | Gastrointestinal Endoscopy | 2009 | Commentary | Abstract, Introduction, Methods,  Results, Discussion/ConclusionAssess manuscript presentation | 24, 26, 27, 30, 31, 33, 35, 36, 40, 46, 51, 62, 63 | 13 |
| Hoyt, K. S.(63) | USA | English | Advanced Emergency Nursing Journal | 2007 | Editorial | Organization and approach to review,  Make general comments,  Title is accurate, Abstract, Introduction,  Results, Discussion/ConclusionReferences,  Address ethical aspects,  Assess manuscript presentation,  Provide recommendations | 1, 11, 15, 18, 21, 22, 23, 26, 27, 45, 50, 51, 56, 57, 62, 63, 65, 66, 67, 68, 70, 71 | 22 |
| Isaacs, D.(64) | Australia | English | Journal of Paediatrics and Child Health | 2004 | Commentary | N/A | N/A | 0 |
| Izumi, S.(65) | USA | English | Japan Journal of Nursing Science | 2009 | Editorial | Make general comments,  Assess manuscript presentation,  Assess manuscript presentation,  Provide recommendations | 11, 12, 14, 62, 64, 71 | 6 |
| Jacobson, R.(66) | USA | English | Academic Paediatrics | 2017 | Commentary | Organization and approach to review,  Make general comments,  Title is accurate, Abstract,  Methods,  Results, Discussion/ConclusionReferences,  Address ethical aspects,  Provide recommendations | 1, 6, 7, 18, 21, 24, 27, 30, 46, 51, 56, 57, 63, 67, 70 | 15 |
| Jain, A. K.(67) | India | English | Indian Journal of Orthopaedics | 2009 | Editorial | Organization and approach to review,  Make general comments,  Results, Discussion/ConclusionAssess manuscript presentation,  Provide recommendations | 1, 13, 14, 46, 51, 63, 70 | 7 |
| Kasiske, B. L.(68) | N/A | English | American Journal of Kidney Diseases | 2005 | Editorial | Organization and approach to review,  Make general comments,  Methods,  Results, Discussion/ConclusionAssess manuscript presentation | 1, 12, 31, 32, 36, 45, 50, 62, 66 | 9 |
| Katz, A.(69) | N/A | English | Oncology Nursing Forum | 2016 | Editorial | Make general comments, Methods, Discussion/Conclusion | 13, 14, 16, 17, 30, 50, 53 | 7 |
| Kearney, M.(70) | UK | English | Research in Nursing & Health | 2016 | Editorial | Organization and approach to review,  Make general comments,  Methods,  Results, Discussion/Conclusion, Address ethical aspects,  Assess manuscript presentation | 1,3, 4, 13, 16, 30, 31, 34, 37, 46, 50, 52, 53, 60, 66, 67, | 16 |
| Kehrer, J. P.(71) | Canada | English | Toxicology | 2013 | Letter | Make general comments | 11, 13, 14 | 3 |
| Kotsis, S.(72) | N/A | English | Plastic and Reconstructive Surgery | 2014 | Editorial | Organization and approach to review, Make general comments, Introduction, Methods,  Results, Discussion/ConclusionReferences,  Address ethical aspects,  Provide recommendations, Provide recommendations | 3, 4, 12, 16, 26, 27, 28, 30, 31, 32, 33, 34, 35, 36, 38, 39, 40, 41, 42, 44, 45, 46, 47, 50, 51, 52, 53, 56, 57, 69, 70, 71 | 32 |
| Kottner, J.(73) | Germany | English | International Journal of Nursing Studies | 2016 | Editorial | Organization and approach to review, Make general comments, Introduction, Methods,  Results, Discussion/ConclusionReferences,  Provide recommendations | 1, 2, 10, 11, 13, 14, 15, 16, 19, 26, 27, 30, 45, 47, 50, 56, 70, 71 | 18 |
| Krome, R. L.(74) | N/A | English | Annals of Emergency Medicine | 1983 | Editorial | Make general comments,  Abstract, Introduction, Methods,  Results, Discussion/ConclusionReferences,  Provide recommendations | 11, 12, 24, 27, 30, 45, 46, 51, 56, 70, 71, 72 | 12 |
| Landauer, A.(75) | Australia | English | Australian Drug and Alcohol Review | 1987 | Editorial | Make general comments, Introduction, Methods, Discussion/ConclusionReferences,  Address ethical aspects,  Assess manuscript presentation,  Provide recommendations | 12, 14, 17, 27, 31, 47, 50, 56, 57, 62, 63, 70, 71 | 13 |
| Lau, D.(76) | N/A | English | Canadian Journal of Diabetes | 2016 | Editorial | Make general comments, Discussion/ConclusionProvide recommendations | 11, 12, 13, 16, 36, 50, 70, 72 | 8 |
| Lichter, P. R.(77) | N/A | English | Ophthalmology | 1993 | Editorial | Abstract, Introduction, Methods, Results, Discussion/Conclusion, References, Assess manuscript presentation, | 24, 26, 27, 28, 29, 30, 32, 35, 36, 37, 38, 45, 46, 47, 49, 50, 51, 54, 56, 63, 65 | 21 |
| Lowe, N.(78) | N/A | English | Journal of Obstetric, Gynecologic, & Neonatal Nursing | 2007 | Editorial | N/A | N/A | 0 |
| Luscher, T.(79) | USA | English | Journal of the American College of Cardiology | 2016 | Letter | Make general comments,  Methods, Discussion/Conclusion | 13, 14, 31, 32, 35, 50 | 6 |
| Mackenzie, L.(80) | Australia | English | Australian Occupational Therapy Journal | 2006 | Editorial | Organization and approach to review, Provide recommendations | 5, 70 | 2 |
| Mannis, M. J.(81) | N/A | English | Cornea | 1997 | Editorial | N/A | N/A | 0 |
| Manske, P.(82) | N/A | English | The Journal of Hand Surgery | 1997 | Commentary | Provide recommendations | 70 | 1 |
| Martin-Sanchez, F.(83) | Spain | English | Emergencias | 2012 | Editorial | N/A | N/A | 0 |
| Marušić, M.(84) | Croatia | English | Croatian Medical Journal | 2005 | Editorial | Title is accurate, Abstract, Introduction, Methods,  Results, Discussion/ConclusionAddress ethical aspects | 21, 24, 27, 30, 46, 51, 57 | 7 |
| Merell, R.(85) | N/A | English | Telemedicine and E-Health | 2007 | Editorial | Make general comments,  Methods, Discussion/ConclusionAssess manuscript presentation | 17, 31, 53, 65, 66 | 5 |
| Minion, D.(86) | N/A | English | Journal of Vascular Surgery | 2007 | Editorial | Make general comments,  Address ethical aspects,  Provide recommendations | 11, 12, 57, 70, 71 | 5 |
| Moher, D.(87) | Canada | English | BMC Medicine | 2015 | Tutorial | N/A | N/A | 0 |
| Moher, D.(88) | Canada | English | Peer review in Medical Science | 2003 | Book chapter | Organization and approach to review, Make general comments, Discussion/ConclusionAssess manuscript presentation,  Provide recommendations | 1, 3, 5, 7, 8, 10, 12, 50, 63, 64, 70, 71 | 12 |
| Moore, K. N.(89) | N/A | English | Journal of Wound, Ostomy, & Continence Nursing | 2005 | Editorial | Make general comments,  Methods,  Results, Discussion/ConclusionAddress ethical aspects | 11, 17, 34, 35, 38, 46, 48, 50, 51, 59, 61 | 11 |
| Morse, J. M. (90) | USA | English | Qualitative Health Research | 2014 | Editorial | Provide recommendations | 70 | 1 |
| Muir-Cochrane, E.(91) | N/A | English | Nursing & Health Sciences | 2013 | Editorial | Organization and approach to review, Assess manuscript presentation | 3, 7, 64, 66 | 4 |
| Nelson, C. A.(92) | USA | English | Dermatologic Clinics | 2009 | Editorial | Organization and approach to review, Abstract, Introduction, Methods,  Results,  References,  Address ethical aspects,  Provide recommendations | 1, 4, 16, 18, 22, 23, 25, 26, 27, 28, 30, 31, 32, 33, 34, 35, 36, 37, 38, 39, 40, 41, 42, 44, 45, 46, 48, 49, 50, 51, 52, 56, 57, 58, 71 | 35 |
| Newell, F. W.(93) | USA | English | American Journal of Ophthalmology | 1990 | Editorial | Make general comments,  Methods,  Results,  References,  Assess manuscript presentation,  Provide recommendations | 11, 12, 13, 14, 16, 30, 33, 34, 36, 45, 49, 50, 56, 62, 63, 66, 71 | 17 |
| Nexoe, J.(94) | Denmark | English | Scandinavian Journal of Primary Health Care | 2014 | Editorial | N/A | N/A | 0 |
| Olson, C. M.(95) | N/A | English | American Journal of Emergency Medicine | 1990 | Editorial | Make general comments, Discussion/ConclusionAssess manuscript presentation,  Provide recommendations | 11, 12, 14, 18, 50, 63, 71 | 7 |
| Oman, K.(96) | USA | English | Journal of Emergency Nursing | 2009 | Editorial | Organization and approach to review,  Make general comments,  Assess manuscript presentation,  Provide recommendations, Provide recommendations | 1, 3, 13, 15, 18, 62, 70, 71, 72 | 9 |
| Pearson, G. S. | USA | English | Journal of the American Psychiatric Nurses Association | 2016 | Editorial | Make general comments,  Methods,  Results, Discussion/ConclusionAddress ethical aspects,  Provide recommendations | 12, 15, 16, 17, 32, 36, 43, 45, 47, 50, 53, 57, 71 | 13 |
| Peh, W.C.G.(98) | Singapore | English | Singapore Medical Journal | 2009 | Editorial | Organization and approach to review,  Title is accurate, Abstract,  Methods,  Results, Discussion/ConclusionReferences,  Assess manuscript presentation,  Provide recommendations | 1, 2, 3, 8, 21, 24, 27, 28, 30, 46, 51, 56, 62, 63, 71 | 15 |
| Perkins, D.(99) | Australia | English | Australian Journal of Rural Health | 2009 | Editorial | N/A | N/A | 0 |
| Pierson, C. A.(100) | N/A | English | Journal of the American Association of Nurse Practitioners | 2014 | Editorial | Make general comments, Introduction, Methods, Results, Discussion/Conclusion, Address ethical aspects, Provide recommendations, | 13, 14, 16, 26, 27, 28, 30, 32, 33, 34, 36, 38, 45, 50, 51, 53, 57, 71, | 18 |
| Pierson, C. A.(101) | N/A | English | Journal of the American Association of Nurse Practitioners | 2015 | Editorial | Make general comments,  Methods,  Address ethical aspects | 13, 16, 38, 57, 60 | 5 |
| Pierson, C. A.(102) | N/A | English | Journal of the American Association of Nurse Practitioners | 2016 | Editorial | Introduction, Methods, Discussion/Conclusion | 26, 27, 28, 30, 50, 51, 54 | 7 |
| Pietrzak, W. S.(103) | N/A | English | Journal of Craniofacial Surgery | 2010 | Editorial | Organization and approach to review, Make general comments,  Title is accurate, Abstract, Introduction, Methods,  Results, Discussion/ConclusionReferences,  Address ethical aspects,  Assess manuscript presentation,  Provide recommendations | 3, 7, 12, 14, 21, 24, 27, 30, 45, 46, 51, 56, 57, 62, 65, 66, 67, 68, 70, 71, 72 | 21 |
| Polak, J. F.(104) | USA | English | American Journal of Roentgenology | 1995 | Perspective | Organization and approach to review, Abstract, Introduction,  Results, Discussion/ConclusionReferences,  Provide recommendations | 3, 24, 27, 30, 45, 46, 51, 56, 70 | 9 |
| Poland, G. A.(105) | USA | English | Vaccine | 2013 | Editorial | Make general comments,  Methods,  References,  Address ethical aspects,  Provide recommendations | 12, 13, 17, 56, 57, 59, 71 | 7 |
| Prado, A.(106) | N/A | English | Plastic and Reconstructive Surgery | 2009 | Editorial | Make general comments,  Methods, Discussion/ConclusionAddress ethical aspects,  Assess manuscript presentation,  Provide recommendations | 11, 12, 13, 14, 31, 50, 57, 64, 65, 67, 70 | 11 |
| Pyne, D.(107) | N/A | English | International Journal of Sports Physiology and Performance | 2011 | Editorial | Organization and approach to review, Make general comments,  Methods,  Results,  Provide recommendations | 8, 11, 12, 15, 16, 31, 35, 45, 66, 72 | 10 |
| Quan, S. F.(108) | USA | English | Journal of Clinical Sleep Medicine | 2014 | Editorial | Make general comments,  Assess manuscript presentation | 11, 13, 62 | 3 |
| Raff, H.(109) | USA | English | Advances in Physiology Education | 2013 | Editorial | N/A | N/A | 0 |
| Ramsden, V. R.(110) | Canada | English | Canadian Family Physician | 2014 | Editorial | N/A | N/A | 0 |
| Relman, A. S.(111) | USA | English | Western Journal of Medicine | 1990 | Commentary | Make general comments,  Methods | 12, 14, 30, 31, 32 | 5 |
| Rennie, D.(112) | USA | English | American Journal of Diseases of Children | 1988 | Editorial | N/A | N/A | 0 |
| Resnick, B.(113) | USA | English | Geriatric Nursing | 2010 | Editorial | Organization and approach to review, Abstract,  Methods,  Results, Discussion/ConclusionReferences,  Address ethical aspects,  Assess manuscript presentation | 1, 3, 22, 30, 35, 45, 46, 48, 50, 51, 56, 57, 63 | 13 |
| Reyes B, H.(114) | N/A | English | Revista Medica De Chile | 2013 | Editorial | N/A | N/A | 0 |
| Richardson, D.(115) | N/A | English | JAVA - Journal of the Association for Vascular Access | 2006 | Editorial | N/A | N/A | 0 |
| Riss, P.(116) | N/A | English | International Urogynecology Journal | 2012 | Editorial | Organization and approach to review, Abstract, Introduction, Methods,  Results,  Provide recommendations | 1, 3, 8, 24, 27, 30, 46, 51, 70 | 9 |
| Roberts, J.(117) | N/A | English | Journal of Sexual Medicine | 2008 | Editorial | Make general comments, Methods, Results, Address ethical aspects, Provide recommendations | 12, 35, 38, 49, 60, 70 | 6 |
| Roberts, L. W.(118) | N/A | English | Academic Psychiatry Win | 2002 | Editorial | Make general comments,  Title is accurate, Abstract, Introduction, Methods,  Results, Discussion/ConclusionReferences,  Address ethical aspects,  Assess manuscript presentation | 15, 16, 20, 21, 24, 26, 29, 31, 33, 34, 35, 36, 46, 50, 51, 56, 57, 63, | 18 |
| Rogers, L. F.(119) | USA | English | American Journal of Roentgenology | 2002 | Editorial | Make general comments,  Methods,  Results, Discussion/ConclusionReferences,  Assess manuscript presentation,  Provide recommendations | 13, 17, 33, 34, 45, 50, 56, 62, 70, 72 | 10 |
| Sadun, A.(120) | N/A | English | Ophthalmology | 2002 | Editorial | Make general comments,  Methods, Discussion/Conclusion | 11, 38, 51, 55 | 4 |
| Salasche, S. J.(121) | N/A | English | Dermatologic Surgery | 1997 | Editorial | Make general comments,  Provide recommendations | 11, 12, 13, 70 | 4 |
| Saper, C. B.(122) | N/A | English | Annals of Neurology | 2014 | Editorial | Organization and approach to review,  Make general comments,  Address ethical aspects,  Assess manuscript presentation | 3, 4, 7, 14, 57, 62, 65, 66 | 8 |
| Sciortino, J. E.(123) | N/A | English | Cuaj-Canadian Urological Association Journal | 2013 | Editorial | Organization and approach to review, Methods, Discussion/Conclusion | 1, 31, 35, 50 | 4 |
| Sellke, F. W.(124) | N/A | English | Journal of Thoracic and Cardiovascular Surgery | 2003 | Editorial | Methods, Results, Discussion/ConclusionAddress ethical aspects | 30, 46, 50, 57 | 4 |
| Sohail, S.(125) | N/A | English | Journal of the College of Physicians and Surgeons Pakistan | 2015 | Editorial | N/A | N/A | 0 |
| Spolarich, A. E.(126) | USA | English | Canadian Journal of Dental Hygiene | 2014 | Editorial | Organization and approach to review, Make general comments,  Results, Discussion/ConclusionReferences,  Assess manuscript presentation,  Provide recommendations | 1, 13, 45, 50, 56, 64, 68, 69, 70, 71 | 10 |
| Stein, K. F.(127) | USA | English | Journal of the American Psychiatric Nurses Association | 2014 | Editorial | Organization and approach to review, Methods | 7, 30 | 2 |
| Swartz, M. K.(128) | N/A | English | Journal of Pediatric Health Care | 2008 | Editorial | Organization and approach to review,  Make general comments,  Address ethical aspects,  Assess manuscript presentation | 1, 5, 11, 12, 15, 57, 63 | 7 |
| Taylor, F. R.(129) | N/A | English | Headache | 2009 | Editorial | Make general comments,  Assess manuscript presentation | 11, 12, 14, 64 | 4 |
| Thombs, B. D.(130) | Canada | English | Canadian Medical Association Journal | 2012 | Editorial | N/A | N/A | 0 |
| Twentyman, P.(131) | UK | English | British Journal of Cancer | 1991 | Editorial | Organization and approach to review, Results, Discussion/ConclusionReferences,  Assess manuscript presentation | 4, 45, 50, 56, 65 | 5 |
| Van Norman, G. A.(132) | N/A | English | Clinical Ethics in Anesthesiology: A Case-Based Textbook | 2011 | Book chapter | N/A | N/A | 0 |
| Vintzileos, A. M.(133) | USA | English | Journal of Ultrasound in Medicine | 2010 | Editorial | Organization and approach to review,  Make general comments,  Methods, Discussion/ConclusionAddress ethical aspects | 2, 16, 26, 30, 31, 32, 34, 35, 39, 40, 50, 51, 52, 53, 54, 57, 58 | 17 |
| Wagner, P.D. (134) | USA | English | Journal of Applied Physiology | 2016 | Editorial | Address ethical aspects | 57 | 1 |
| Wick, G.(135) | Austria | English | International Archives of Allergy and Immunology | 1996 | Editorial | Make general comments | 11, 12, 13 | 3 |
| Wierzbinski-Cross, H.(136) | N/A | English | Journal for Nurses in Professional Development | 2017 | Editorial | Make general comments, Abstract, Introduction, Methods, Results, Discussion/Conclusion, References, Assess manuscript presentation, Provide recommendations, | 11, 15, 16, 17, 18, 22, 26, 29, 31, 32, 34, 45, 51, 56, 62, 65, 66, 68, 69, 70, 71 | 21 |
| Wilder, R. S.(137) | N/A | English | Journal of Dental Hygiene | 2014 | Editorial | Methods, Discussion/ConclusionReferences,  Assess manuscript presentation | 35, 53, 56, 66 | 4 |
| Wolf, L. A.(138) | N/A | English | Journal of Emergency Nursing | 2016 | Editorial | Make general comments,  Methods,  Results,  Assess manuscript presentation | 17, 33, 34, 37, 43, 44, 46, 66, 67 | 9 |
| Young, S. N. (139) | Canada | English | Journal of Psychiatry & Neuroscience | 2003 | Editorial | Methods | 30, 33 | 2 |
| Zinsky, R.(140) | Germany | English | Breathe | 2014 | Editorial | Organization and approach to review, Make general comments, Introduction, Methods | 1, 3, 4, 13, 27, 30 | 6 |
| **Total** |  |  |  |  |  |  |  | 1085 |

^B^ Corresponds to item number from the list of tasks (Table 4)

^C^ Number of extracted task statements

## References

1. Alam S, Patel J. Peer review: tips from field experts for junior reviewers. Bmc Med. 2015 Nov;13.

2. Allen TW. Peer review guidance: how do you write a good review? JAOA J Am Osteopath Assoc. 2013 Dec;113(12):916–20.

3. Allen TW. Conducting Proper Peer Review for a Journal. Bariatr Surg Pract Patient Care. 2014 Mar;9(1):18–20.

4. Andersson KE. Peer Review: “A Critique of the Critics.” J Urol. 2011 Sep;186(3):777–8.

5. Bacchetti P. Peer review of statistics in medical research: the other problem. Br Med J. 2002 May;324(7348):1271–3.

6. Baker JD. Artistry of Peer Review. Aorn J. 2015 Jan;101(1):4–11.

7. Bernstein J, Wager E, Heckman JD, Zeller JL. Free for Service: The Inadequate Incentives for Quality Peer Review. Clin Orthop. 2013 Oct;471(10):3093–7.

8. Berquist TH. Peer Review: Should We Modify Our Process? Am J Roentgenol. 2014 Mar;202(3):463–4.

9. Brandon D, McGrath JM. Conducting a Peer Review Novice or Expert. Adv Neonatal Care. 2015 Dec;15(6):365–6.

10. Brock WJ. Strengthening the Peer Review Process for the International Journal of Toxicology. Int J Toxicol. 2014 Sep;33(5):351–2.

11. Byrne RA. Peer review at EuroIntervention - a rough guide and an expression of thanks. Eurointervention. 2016 Nov;12(10):1197-+.

12. Campion EW, Curfman GD, Drazen JM. Tracking the peer-review process. N Engl J Med. 2000 Nov;343(20):1485–6.

13. Carrio I. On cloning research, peer review and the possibility of fraud. Eur J Nucl Med Mol Imaging. 2006 Mar;33(3):235–6.

14. Chew FS. Manuscript Peer-Review - General Concepts and the Ajr Process. Am J Roentgenol. 1993 Feb;160(2):409–11.

15. Christensen NB, Yokomizo A. How to Peer Review. Int J Urol. 2010 Sep;17(9):754–754.

16. Clark RKF. Peer review: a view based on recent experience as an author and reviewer. Br Dent J. 2012 Aug;213(4):153–4.

17. Clarke SP. Reviewing peer review: the three reviewers you meet at submission time. Can J Nurs Res. 2006 Dec;38(4):5–9.

18. Cotton P. Flaws Documented, Reforms Debated at Congress on Journal Peer-Review (Vol 270, Pg 2775, 1993). Jama-J Am Med Assoc. 1994 Jul;272(1):11–11.

19. Cowell JM. Importance of Peer Review. J Sch Nurs. 2014 Dec;30(6):394–5.

20. Cowell JM. Peer-Review Responsibility. J Sch Nurs. 2015 Dec;31(6):395.

21. Crawford S. Peer review and the evaluation of manuscripts. Bull Med Libr Assoc. 1988 Jan;76(1):75–7.

22. Cummings P, Rivara FP. Reviewing manuscripts for Archives of Pediatrics & Adolescent Medicine. Arch Pediatr Adolesc Med. 2002 Jan;156(1):11–3.

23. Cusick A. Peer review: Least-worst approach or the very best we can do? Aust Occup Ther J. 2016 Feb;63(1):1–4.

24. da Cruz IC. Peer review: The case of online Brazilian Journal of nursing. Online Braz J Nurs [Internet]. 2008;7(3). Available from: https://www.scopus.com/inward/record.uri?eid=2-s2.0-77953333040&partnerID=40&md5=6bb5b65861b5f7542e86b5f1b9e91e6c

25. David B, Karadottir RT. Writing a constructive peer review: a young PI perspective. Eur J Neurosci. 2016 Dec;44(11):2873–6.

26. de Araujo CGS. Peer Review: a Constantly-Evolving Scientific Process. Arq Bras Cardiol. 2012 Feb;98(2):E32–5.

27. de Hon F, Poland GA. Submission of articles to Vaccine: A fast and fair peer review process. Vaccine. 2013 Jul;31(32):3207–8.

28. DeBarr K. Meaningful peer review. Californian J Health Promot. 2007;5(3):70–2.

29. Del Mar C, Hoffmann TC. A guide to performing a peer review of randomised controlled trials. BMC Med. 2015 Nov 2;13:1–7.

30. Dinis-Ribeiro M, Vakil N, Ponchon T. The Editors’ guide for peer review of papers submitted to Endoscopy. Endoscopy. 2013 Jan;45(1):48–50.

31. Do Vale ES, Gontijo B, Marques SA. Obligations and responsibilities of the peer reviewers. [Portuguese] Deveres e responsabilidades dos pareceristas. An Bras Dermatol. 2008 Jul;83(4):281–2.

32. Donato H, Marinho RT. Acta Medica Portuguesa and Peer-review: Quick and Brutal! Acta Med Port. 2012 Sep;25(5):261–2.

33. Drummond A. Reviewing a research article [Internet]. 1996 [cited 1959 Jan 1]. Available from: http://ovidsp.ovid.com/ovidweb.cgi?T=JS&PAGE=reference&D=psyc3&NEWS=N&AN=1999-00420-003

34. Dutta MJ. The Ten Commandments of Reviewing: The Promise of a Kinder, Gentler Discipline! [Internet]. 2006 [cited 1995 Jan 18]. Available from: http://ovidsp.ovid.com/ovidweb.cgi?T=JS&PAGE=reference&D=psyc5&NEWS=N&AN=2006-12308-011

35. el-Azhary RA. The last bastion of collegiality: the peer-review process in the era of open access. Int J Dermatol. 2016 Mar;55(3):247–8.

36. Emanuel LL, Greenland P. Peer review and professionalism at the Archives of Internal Medicine. Arch Intern Med. 2005 Dec;165(22):2559–60.

37. Emden C. Manuscript reviewing: too long a concealed form of scholarship? [see comments]. Nurs Inq. 1996;(4):195–9.

38. Faggion CM. Improving the peer-review process from the perspective of an author and reviewer. Br Dent J. 2016 Feb;220(4):167–8.

39. Fain JA. Guidelines informing the peer review process [Internet]. 2011 [cited 2004 Jan 4]. Available from: http://ovidsp.ovid.com/ovidweb.cgi?T=JS&PAGE=reference&D=psyc8&NEWS=N&AN=2011-21278-001

40. Feinstein AR, Spitzer WO. The Peer-Review Process - and an Acknowledgment of Our Peerless Reviewers. J Clin Epidemiol. 1989;42(1):1–4.

41. Feldstein Ewing SW, Saitz R. Peer review of human studies run amok: A break in the fiduciary relation between scientists and the public. Evid Based Med. 2015;20(1):1–2.

42. Feldman MD, Kravitz RL. Peer Review at JGIM. J Gen Intern Med. 2016 Dec;31(12):1401–7.

43. Ferris LE, Brumback RA. Academic merit, promotion, and journal peer reviewing: The role of academic institutions in providing proper recognition [Internet]. 2010. Available from: http://ovidsp.ovid.com/ovidweb.cgi?T=JS&PAGE=reference&D=psyc7&NEWS=N&AN=2010-08642-001

44. Fisher RS, Powers LE. Peer-reviewed publication: A view from inside. Epilepsia. 2004 Aug;45(8):889–94.

45. Fitzpatrick JJ. The Peer Review Process Revisited. Appl Nurs Res. 2017 Feb;33:186–186.

46. Flood AB. From the editors: External peer review at HSR. Health Serv Res. 2004 Oct;39(5):1235–50.

47. Fontes BM. Peer review. Arq Bras Oftalmol. 2015 Jan;78(1):V–VIII.

48. Foster RL. A primer on peer review. J Spec Pediatr Nurs. 2008 Jan;13(1):1–3.

49. Friedman DP. Manuscript Peer-Review at the Ajr - Facts, Figures, and Quality Assessment. Am J Roentgenol. 1995 Apr;164(4):1007–9.

50. Furness P. Promoting Research into Peer-Review - Referees Should Provide References. Br Med J. 1994 Aug;309(6953):539–539.

51. Gennaro S. Peer Review: We Can’t Do Without You. J Nurs Scholarsh. 2015 Nov;47(6):485–6.

52. Gitanjali B. Peer review - Process, perspectives and the path ahead. J Postgrad Med. 2001;47(3):210–4.

53. Giunta RE, Prommersberger KJ. A guide to the peer review of scientific papers. [German] Empfehlungen fur die Begutachtung von wissenschaftlichen Manuskripten im Peer-Review"-Prozess. Handchir Mikrochir Plast Chir. 2012;44(4):193–7.

54. Glick M. Peer review: An inexact but essential part of scientific publishing (vol 138, pg 568, 2007). J Am Dent Assoc. 2007 Jun;138(6):730–730.

55. Goldbeck-Wood S. What makes a good reviewer of manuscripts? The BMJ invites you to join its peer review process. Br Med J. 1998 Jan;316(7125):86–86.

56. Goodlett CR. Peer review: Honoring service and facilitating communication with electronic submission and review. Alcohol. 2006 Nov;37(3):125–7.

57. Gough NR. Training for peer review. Sci Signal [Internet]. 2009;2(85). Available from: https://www.scopus.com/inward/record.uri?eid=2-s2.0-70449727093&doi=10.1126%2fscisignal.285tr2&partnerID=40&md5=9e0b5836bfb1d4056660326264e6996f

58. Halder N, Ramsay R, Tyrer P, Casey P. Peer reviewing made easy. Adv Psychiatr Treat. 2011;17(2):150–7.

59. Harms M. Peer review: the firewall of science. Physiotherapy. 2006 Dec;92(4):193–4.

60. Heddle NM, Ness PM. Reviewing manuscripts: tips and responsibilities. Transfusion (Paris). 2009 Nov;49(11):2265–8.

61. Helton ML, Balistreri WF. Peering into Peer-Review. J Pediatr. 2011 Jul;159(1):150–1.

62. Hernandez LV. Becoming a reviewer is good for you-the peer-review process. Gastrointest Endosc. 2009 Dec;70(6):1159–60.

63. Hoyt KS, Proehl JA. Peer review for professional publications. Adv Emerg Nurs J. 2007;29(3):260–4.

64. Isaacs D. Who teaches the referee? J Paediatr Child Health. 2004 Jul;40(7):397–8.

65. Izumi S. Roles and rewards of journal peer reviewers. Jpn J Nurs Sci JJNS. 2009 Dec;6(2):67–9.

66. Jacobson RM, Fairbrother G, Sheldrick RC, Szilagyi PG. The Role of the Peer Reviewer. Acad Pediatr. 2017 Mar;17(2):105–6.

67. Jain AK. Peer review: Heart and soul of scientific publication. Indian J Orthop. 2009 Jan;43(1):3–5.

68. Kasiske BL, Chavers BM, Foley RN, Swan SK, Rosenberg ME. Just rewards: continuing medical education credit for peer review of manuscripts. Am J Kidney Dis. 2005 Dec;46(6):995–6.

69. Katz A. Upon Further Review: Peer Process Vital to Publishing. Oncol Nurs Forum. 2016 Nov;43(6):675–6.

70. Kearney MH. Rigorous Peer Review is Worth the Effort. Res Nurs Health. 2016 Dec;39(6):393–5.

71. Kehrer JP. Editors’ and reviewers’ roles in promoting quality publications. Toxicol Lett. 2013 Aug 28;221:S37.

72. Kotsis SV, Chung KC. Manuscript rejection: How to submit a revision and tips on being a good peer reviewer. Plast Reconstr Surg. 2014;133(4):958–64.

73. Kottner J, Norman I. How to peer review and revise manuscripts submitted for publication in academic nursing journals. Int J Nurs Stud. 2016 Dec;64:A1–3.

74. Krome RL. Peer-Review. Ann Emerg Med. 1983;12(1):57–57.

75. Landauer AA. Peer Review and the Editorial Process. Aust Drug Alcohol Rev. 1987;6(2):107–9.

76. Lau DCW. Tackling Peer Review: How to Improve Reviews and Minimize Abuse. Can J Diabetes. 2016 Apr;40(2):105–6.

77. Lichter PR. Demystifying the Peer-Review Process. Ophthalmology. 1993 Dec;100(12):1749–50.

78. Lowe NK. Peer (review) pressure. Jognn-J Obstet Gynecol Neonatal Nurs. 2007 Mar;36(2):115–115.

79. Luscher TF. RESPONSE: Why Do We Need Peer Review? J Am Coll Cardiol. 2016 May;67(17):2081–2.

80. Mackenzie L. Peer review revisited [Internet]. 2006. Available from: http://ovidsp.ovid.com/ovidweb.cgi?T=JS&PAGE=reference&D=psyc5&NEWS=N&AN=2006-20698-001

81. Mannis MJ, Sugar J. Peer review and Cornea. Cornea. 1997 Nov;16(6):601–601.

82. Manske PR. A review of peer review. J Hand Surg-Am Vol. 1997 Sep;22A(5):767–71.

83. Martin-Sanchez FJ, Miro O. Reflections on the peer review of manuscripts sent to Emergencias. Emergencias. 2012 Apr;24(2):81–3.

84. Marušić M, Sambunjak D, Marušić A. Guide for peer reviewers of scientific articles in the Croatian Medical Journal. Croat Med J. 2005;46(2):326–32.

85. Merrell RC, Doarn CR. The importance of peer review. Telemed J E Health. 2007 Dec;13(6):613–4.

86. Minion D, Sorial E, Endean E. Ethics of guidelines for reviewers of medical manuscripts. J Vasc Surg. 2007 Aug;46(2):391–3.

87. Moher D. Optimal strategies to consider when peer reviewing a systematic review and meta-analysis. Bmc Med. 2015 Nov;13.

88. Moher D, Jadad A. How to peer review a manuscript. Peer Rev Health Sci BMJ Books Lond. 2003;183–90.

89. Moore KN. Keeping up journal integrity: the peer-review process. J Wound Ostomy Continence Nurs. 2005 Jan;32(1):3–5.

90. Morse JM. Cooperative scholarship: The peer review process [Internet]. 2014 [cited 2012 Jan 10]. Available from: http://ovidsp.ovid.com/ovidweb.cgi?T=JS&PAGE=reference&D=psyc11&NEWS=N&AN=2014-45705-001

91. Muir-Cochrane E. What do journal editors want? ... and everything you wanted to know about the peer review process for journal publication. Nurs Health Sci. 2013 Sep;15(3):263–4.

92. Nelson CA, Freeman SR, Dellavalle RP. Reviewing dermatology manuscripts and publications. Dermatol Clin. 2009 Apr;27(2):201–viii.

93. Newell FW. Peer-Review. Am J Ophthalmol. 1990 Feb;109(2):221–3.

94. Nexoe J. As you are a recognized expert in this field, we kindly ask if you would be willing to review this manuscript [Internet]. 2014. Available from: http://ovidsp.ovid.com/ovidweb.cgi?T=JS&PAGE=reference&D=psyc11&NEWS=N&AN=2014-55575-001

95. Olson CM. Peer-Review of the Biomedical Literature. Am J Emerg Med. 1990 Jul;8(4):356–8.

96. Oman K. Peer Review: The Art of Supporting Colleagues and Advancing Our Profession. J Emerg Nurs. 2009 Jul;35(4):278–278.

97. Pearson GS. Peer Review. J Am Psychiatr Nurses Assoc. 2016 Sep;22(5):365–6.

98. Peh WCG, Ng KH. Role of the manuscript reviewer. Singapore Med J. 2009 Oct;50(10):931–3; quiz 934.

99. Perkins D. Peer review: The key to quality and relevance. Aust J Rural Health. 2009 Oct;17(5):229–30.

100. Pierson CA. Some thoughts on how to do good quality peer review. J Am Assoc Nurse Pract. 2014 Feb;26(2):57–8.

101. Pierson CA. Peer Review Week 2015. J Am Assoc Nurse Pract. 2015 Nov;27(11):605–605.

102. Pierson CA. Make your peer reviews more organized and systematic with checklists. J Am Assoc Nurse Pract. 2016 Dec;28(12):637–8.

103. Pietrzak WS. A practical guide to effective journal peer reviewing. J Craniofac Surg. 2010 May;21(3):631–6.

104. Polak JF. The Role of the Manuscript Reviewer in the Peer-Review Process. Am J Roentgenol. 1995 Sep;165(3):685–8.

105. Poland GA. The Importance of Peer Review. Vaccine. 2013 Jan;31(4):567–83.

106. Prado A, Andrades P. Standing up for the professionalism of peer reviewers of plastic surgery manuscripts. Plast Reconstr Surg. 2009 Dec;124(6):2185–6.

107. Pyne D. Improving the Peer Review Process. Int J Sports Physiol Perform. 2011 Jun;6(2):145–6.

108. Quan SF. Expediting Peer Review: Just Say No. J Clin Sleep Med. 2014;10(9):941–941.

109. Raff H, Brown D. Civil, sensible, and constructive peer review in APS journals. Adv Physiol Educ. 2013 Sep;37(3):211–2.

110. Ramsden VR, Pimlott N, Woollard R, Kvern B, Handford C, Dunikowski L, et al. Becoming a peer reviewer: Engaging in sharing and gaining knowledge. Can Fam Physician. 2014 Dec;60(12):1158–60.

111. Relman AS. Peer-Review in Scientific Journals - What Good Is It. West J Med. 1990 Nov;153(5):520–2.

112. Rennie D, Knoll E. Investigating Peer-Review. Ann Intern Med. 1988 Aug;109(3):181–181.

113. Resnick B. The Peer Review Process: Some Gentle Reminders for Us All. Geriatr Nur (Lond). 2010 Sep;31(5):321–3.

114. Reyes B H, Andresen H M, Palma H J. The manuscripts’ review process in Revista Médica de Chile and its peer-reviewers during the year 2012. Rev Med Chil. 2013;141(7):903–8.

115. Richardson D, Vesely T, Costa N, Dean S, Moureau N, Wise M. JAVA revises peer-reviewer guidelines. J Assoc Vasc Access. 2006;11(2):98–100.

116. Riss P. The peer review process I: submitting a manuscript. Int Urogynecology J. 2012 Mar;23(3):253–4.

117. Roberts J, Schena D. Peer review and The journal of sexual medicine: Management and collaborative effort. J Sex Med. 2008 May;5(5):1029–32.

118. Roberts LW. On the centrality of peer review. Acad Psychiatry. 2002 Win;26(4):221–2.

119. Rogers LF. Peer reviewers: Reviewing manuscripts for the AJR. Am J Roentgenol. 2002 May;178(5):1051–2.

120. Sadun A, Cox TA, Minckler D. Peer review: Integrating input from authors, reviewers, and editors. Ophthalmology. 2002 Aug;109(8):1419–20.

121. Salasche SJ. How to “‘peer review’” a medical journal manuscript. Dermatol Surg. 1997 Jun;23(6):423–8.

122. Saper CB. Academic publishing, part I: Peering into the review process. Ann Neurol. 2014 Feb;75(2):175–7.

123. Sciortino JE, Siemens DR. The editorial process: Peer review. Cuaj-Can Urol Assoc J. 2013 Jul;7(7–8):225–7.

124. Sellke FW. The peer-review process in medical publishing: A reviewer’s perspective. J Thorac Cardiovasc Surg. 2003 Dec;126(6):1683–5.

125. Sohail S. Fortifying the External Peer Review: An Editorial Perspective. Jcpsp-J Coll Physicians Surg Pak. 2015 Jan;25(1):2–3.

126. Spolarich AE, Wilder RS. Becoming an effective journal reviewer. Can J Dent Hyg. 2014 Nov;48(4):197–9.

127. Stein KF. Can These Results Be Trusted: The Peer Review Process and Its Role in Quality Assurance. J Am Psychiatr Nurses Assoc. 2014 May;20(3):178–178.

128. Swartz MK. The Importance of Peer Review. J Pediatr Health Care. 2008 Nov;22(6):333–4.

129. Taylor FR. Peer Review: The Success of Headache Depends On Us! Headache. 2009 Apr;49:S66–9.

130. Thombs BD, Razykov I. A solution to inappropriate self-citation via peer review. Can Med Assoc J. 2012 Nov;184(16):1864–1864.

131. Twentyman P, Selby P. The Process of Peer-Review. Br J Cancer. 1991 Feb;63(2):168–70.

132. Van Norman GA, Jackson S. Publication ethics: Obligations of authors, Peer-Reviewers, and editors. In: Clinical Ethics in Anesthesiology: A Case-Based Textbook [Internet]. Cambridge University Press; 2010. p. 209–16. Available from: https://www.scopus.com/inward/record.uri?eid=2-s2.0-84926980442&doi=10.1017%2fCBO9780511841361.037&partnerID=40&md5=45e7cf9ffb6d6cd7ef49243a0cf9bb53

133. Vintzileos AM, Ananth CV. The Art of Peer-Reviewing an Original Research Paper Important Tips and Guidelines. J Ultrasound Med. 2010 Apr;29(4):513–8.

134. Wagner PD, Bates JHT. Maintaining the integrity of peer review. J Appl Physiol. 2016 Mar;120(5):479–80.

135. Wick G. The peer review system - Still no alternative in sight. Int Arch Allergy Immunol. 1996 Jan;109(1):1–2.

136. Wierzbinski-Cross H. Peer Review. J Nurses Prof Dev. 2017;33(2):102–4.

137. Wilder RS. Becoming a reviewer for a peer-reviewed journal. J Dent Hyg. 2014 Oct;88(5):262.

138. Wolf LA. The Peer Review Process. J Emerg Nurs. 2016 Sep;42(5):454–6.

139. Young SN. Peer review of manuscripts: theory and practice. J Psychiatry Neurosci. 2003 Sep;28(5):327–30.

140. Zinsky R, Skoczynski S, Jacinto T, Bjerg A. Doing Science: Peer reviewing. Breathe. 2014 Dec;10(4):337–40.
