# Supplementary material for: A scoping review on the roles and tasks of peer reviewers in the manuscript review process in biomedical journals
Source: BMC Med. 2019 Jun 20;17:118. doi: 10.1186/s12916-019-1347-0 (PMC6585141; doi:10.1186/s12916-019-1347-0)
Supplement: Supplementary file 5 — Grey literature. (DOCX 32 kb) [file 12916_2019_1347_MOESM5_ESM.docx]

**Additional file 5 – Search of networks and publishers**

**Table 1** Roles from networks and publishers

| **Title** | **Editorial group / organisation** | **Author** | **Source type** | **Theme(s)** | **Role item(s)** | **# of role (s)** |
| --- | --- | --- | --- | --- | --- | --- |
| A beginner’s guide to peer review: Part One | BioMed Central (BMC) | Jigisha Patel | Blog  <http://blogs.biomedcentral.com/bmcblog/2015/05/13/beginners-guide-peer-review-part-one/> | Proficient experts in their field,  Familiar with journal, Unbiased and ethical professionals,  Self-critical professionals,  Reliable professionals | 1, 2, 11, 13, 15, 17, 31, 36, 38 | 9 |
| A beginner’s guide to peer review: Part Two | BioMed Central (BMC) | Jigisha Patel | Blog  <http://blogs.biomedcentral.com/bmcblog/2015/06/08/beginners-guide-peer-review-part-two/> | Dutiful/altruistic towards scientific community | 6 | 1 |
| A beginner’s guide to peer review: Part Three | BioMed Central (BMC) | Jigisha Patel | Blog  <http://blogs.biomedcentral.com/bmcblog/2015/07/09/beginners-guide-peer-review-part-three/> | Reviewers should not… | 76 | 1 |
| The peer review process – what happens when you send your  manuscript to a journal | BioMed Central (BMC) | Hilary Logan,  Diana Marshal,  Liz Hoffman | Webinar  <http://www.healthsystemsglobal.org/upload/other/HSG-BMC-webinar-2.pdf> | Proficient experts in their field,  Dutiful/altruistic towards scientific community, Reviewers should not… | 1, 2 ,4, 72 | 4 |
| COPE Ethical Guidelines for Peer Reviewers | Committee on Publication Ethics (COPE) | Irene Hames | Guidelines  <https://publicationethics.org/files/Ethical_guidelines_for_peer_reviewers_0.pdf> | Dutiful/altruistic towards scientific community, Familiar with journal, Unbiased and ethical professionals,  Self-critical professionals, Reliable professionals,  Skilled critics,  Respectful communicators, Advisors,  Reviewers should not… | 4, 11, 13, 14, 15, 16, 17, 18, 19, 20, 21, 29, 31, 32, 35, 36, 38, 39, 42, 43, 46, 47, 51, 57, 71, 73 | 26 |
| Peer reviewer training part II: What do editors want from reviewers? | BMJ | Trish Groves | Training  <https://www.bmj.com/about-bmj/resources-reviewers/training-materials> | Dutiful/altruistic towards scientific community, Unbiased and ethical professionals,  Reliable professionals,  Skilled critics,  Respectful communicators, Reviewers should not… | 6, 13, 14, 35, 39, 42, 43, 57, 58, 61, 72 | 11 |
| How to write a thorough peer review | Nature | Mathew Stiller-Reeve | Career column  <https://www.nature.com/articles/d41586-018-06991-0> | Dutiful/altruistic towards scientific community,  Self-critical professionals, Skilled critics,  Respectful communicators, Reviewers should not… | 6, 32, 42, 57, 58, 73 | 6 |
| Focus on Peer Review - Peer reviewers responsibility | Nature masterclass | Andrea Aguilar | Training  <https://masterclasses.nature.com/courses/205> | Familiar with journal, Unbiased and ethical professionals,  Reliable professionals,  Skilled critics | 11, 14, 15, 35, 36, 42 | 6 |
| Focus on Peer Review -Review strategies | Nature masterclass | Andrea Aguilar | Training  <https://masterclasses.nature.com/courses/205> |  |  | 0 |
| What are 'excellent' reviews? | Publons | Publons | Training  <https://publons.freshdesk.com/support/solutions/articles/12000022369-what-are-excellent-reviews-> | Reliable professionals,  Skilled critics,  Respectful communicators, Reviewers should not… | 35, 42, 44, 48, 49, 61, 74 | 7 |
| Reviewer roles and responsibilities | Council of Science Editors | Council of Science Editors | Guidelines  <https://www.councilscienceeditors.org/> | Familiar with journal, Unbiased and ethical professionals,  Reliable professionals,  Skilled critics,  Respectful communicators | 11, 13, 14, 15, 17, 18, 20, 24, 35, 40, 42, 57, 59 | 13 |
| Reviewer guidelines and best practice | Taylor & Francis | Leila Jones | Guide  [https://editorresources.taylorandfrancisgroup.com/reviewers-guidelines-and-best-practice/#](https://editorresources.taylorandfrancisgroup.com/reviewers-guidelines-and-best-practice/) | Proficient experts in their field,  Dutiful/altruistic towards scientific community, Familiar with journal, Unbiased and ethical professionals,  Self-critical professionals, Reliable professionals,  Skilled critics | 1, 5, 11, 13, 34, 36, 39, 43, 46 | 9 |
| How to perform a peer review | Wiley | Wiley | Guide  <https://authorservices.wiley.com/Reviewers/journal-reviewers/how-to-perform-a-peer-review/index.html> | Dutiful/altruistic towards scientific community, Unbiased and ethical professionals,  Self-critical professionals, Reliable professionals,  Skilled critics,  Respectful communicators, Advisors,  Reviewers should not… | 4, 6, 13, 14, 15, 17, 18, 19, 20, 21, 28, 29, 31, 32, 34, 35, 36, 38, 39, 40, 41, 42, 45, 51, 57, 59 , 71, 73, 74 | 29 |
| **Total** |  |  |  |  |  | **122** |

**Table 2** Tasks from networks and publishers

| **Title** | **Editorial group / organisation** | **Author** | **Source type** | **Theme(s)** | **Task item(s)** | **# of task (s)** |
| --- | --- | --- | --- | --- | --- | --- |
| A beginner’s guide to peer review: Part One | BioMed Central (BMC) | Jigisha Patel | Blog  <http://blogs.biomedcentral.com/bmcblog/2015/05/13/beginners-guide-peer-review-part-one/> | Make general comments, Introduction,  Methods, Discussion/Conclusion | 13, 15, 26, 31, 50 | 5 |
| A beginner’s guide to peer review: Part Two | BioMed Central (BMC) | Jigisha Patel | Blog  <http://blogs.biomedcentral.com/bmcblog/2015/06/08/beginners-guide-peer-review-part-two/> | Organization and approach to review,  Make general comments, Introduction,  Methods,  Results, Discussion/Conclusion, References,  Address ethical aspects, Assess manuscript presentation,  Provide recommendations | 3, 4, 13, 19, 26, 27, 31, 33, 37, 45, 46, 50, 56, 57, 58, 60, 62, 70 | 18 |
| A beginner’s guide to peer review: Part Three | BioMed Central (BMC) | Jigisha Patel | Blog  <http://blogs.biomedcentral.com/bmcblog/2015/07/09/beginners-guide-peer-review-part-three/> | Provide recommendations | 70 | 1 |
| The peer review process – what happens when you send your  manuscript to a journal | BioMed Central (BMC) | Hilary Logan,  Diana Marshal,  Liz Hoffman | Webinar  <http://www.healthsystemsglobal.org/upload/other/HSG-BMC-webinar-2.pdf> | Make general comments, Methods, Discussion/Conclusion, Address ethical aspects, Assess manuscript presentation,  Provide recommendations | 11, 13, 14, 16, 30, 32, 50, 57, 63, 64, 67, 71 | 12 |
| COPE Ethical Guidelines for Peer Reviewers | Committee on Publication Ethics (COPE) | Irene Hames | Guidelines  <https://publicationethics.org/files/Ethical_guidelines_for_peer_reviewers_0.pdf> | N/A | N/A | 0 |
| Peer reviewer training part II: What do editors want from reviewers? | BMJ | Trish Groves | Training  https://www.bmj.com/about-bmj/resources-reviewers/training-materials | Organization and approach to review,  Make general comments, Abstract,  Methods,  Results, Discussion/Conclusion, References,  Address ethical aspects | 1, 12, 14, 15, 17, 24, 30, 31, 32, 33, 34, 36, 37, 45, 50, 56, 57 | 17 |
| How to write a thorough peer review | Nature | Mathew Stiller-Reeve | Career column  <https://www.nature.com/articles/d41586-018-06991-0> | Organization and approach to review,  Abstract,  Introduction,  Methods,  Results,  Assess manuscript presentation | 4, 23, 29, 37, 45, 47, 48, 67 | 8 |
| Focus on Peer Review (peer reviewers responsibility) | Nature masterclass | Andrea Aguilar | Training | N/A | N/A | 0 |
| Focus on Peer Review (review strategies) | Nature masterclass | Andrea Aguilar | Training | Organization and approach to review,  Make general comments, Methods,  Results, Discussion/Conclusion, Assess manuscript presentation,  Provide recommendations | 2,3,4,5, 11, 13, 18, 31, 45, 50, 64, 70 | 12 |
| What are 'excellent' reviews? | Publons | Publons | Training  <https://publons.freshdesk.com/support/solutions/articles/12000022369-what-are-excellent-reviews-> | Make general comments, Title is accurate,  Abstract,  Introduction,  Methods,  Results, Discussion/Conclusion, References,  Address ethical aspects, Assess manuscript presentation | 14, 21, 22, 26, 27, 29, 30, 31, 32, 33, 35, 36, 37, 39, 43, 46, 47, 50, 51, 56, 57, 66 | 22 |
| Reviewer roles and responsibilities | Council of Science Editors | Council of Science Editors | Guidelines  <https://www.councilscienceeditors.org/> | Abstract,  Introduction, Methods,  Results, Discussion/Conclusion | 24, 26, 29, 37, 45, 47, 48, 50 | 8 |
| Reviewer guidelines and best practice | Taylor & Francis | Leila Jones | Guide  [https://editorresources.taylorandfrancisgroup.com/reviewers-guidelines-and-best-practice/#](https://editorresources.taylorandfrancisgroup.com/reviewers-guidelines-and-best-practice/) | Make general comments, Methods,  References,  Provide recommendations | 12,16, 36, 37, 56, 70, 71 | 7 |
| How to perform a peer review | Wiley | Wiley | Guide  <https://authorservices.wiley.com/Reviewers/journal-reviewers/how-to-perform-a-peer-review/index.html> | N/A | N/A | 0 |
| **Total** |  |  |  |  |  | **110** |
