# Supplementary material for: A scoping review on the roles and tasks of peer reviewers in the manuscript review process in biomedical journals
Source: BMC Med. 2019 Jun 20;17:118. doi: 10.1186/s12916-019-1347-0 (PMC6585141; doi:10.1186/s12916-019-1347-0)
Supplement: Supplementary file 3 — Reviews. (DOCX 122 kb) [file 12916_2019_1347_MOESM3_ESM.docx]

### Additional file 3

**Table 1** Roles from review articles

| **Author** | **Country** | **Language** | **Journal** | **Year** | **Design** | **Theme(s)** | **Role item(s)^b^** | **# of role(s)^c^** |
| --- | --- | --- | --- | --- | --- | --- | --- | --- |
| Alexander, G.(1) | USA | English | Maternal & Child Health Journal | 2005 | Review article | Dutiful/altruistic towards scientific community,  Unbiased and ethical professionals,  Self-critical professionals,  Reliable professionals,  Skilled critics | 4, 14, 17, 19, 25, 32, 35, 42, 43, 44, 48, 58, 63 | 13 |
| Alfonso, F.(2) | Spain | Spanish | Archivos de Cardiologia de Mexico | 2010 | Review article | Proficient experts in their field,  Dutiful/altruistic towards scientific community,  Unbiased and ethical professionals,  Reliable professionals,  Skilled critics,  Educators,  Advocates | 1, 4, 5, 13, 14, 16, 35, 40, 41, 42, 43, 44, 47, 51, 54, 65, 67 | 17 |
| Ali, P. A.(3) | UK | English | Nursing Open | 2016 | Review article | Unbiased and ethical professionals,  Self-critical professionals,  Reliable professionals,  Skilled critics | 13, 14, 16, 18, 31, 35, 42 | 7 |
| Barroga, E.(4) | Japan | English | Journal of Korean Medical Science | 2014 | Review article | Proficient experts in their field,  Dutiful/altruistic towards scientific community,  Skilled critics,  Educators, | 1, 2, 3, 10, 45, 65, | 6 |
| Baxt, W. G.(5) | USA | English | Annals of Emergency Medicine | 1998 | Review article | Skilled critics | 43, 44, 55 | 3 |
| Bayne, S. C.(6) | USA | English | The Journal of Prosthetic Dentistry | 2003 | Review article | Dutiful/altruistic towards scientific community,  Familiar with journal,  Reliable professionals,  Skilled critics,  Advocates,  Reviewers should not | 7, 11, 36, 37, 46, 67, 73 | 7 |
| Bingham, C. L.(7) | Australia | English | THE LANCET | 1998 | Review article | Proficient experts in their field,  Reliable professionals,  Skilled critics,  Respectful communicators | 1, 35, 47, 50, 58 | 5 |
| Brand, R. A. (8) | USA | English | Clinical Orthopaedics and Related Research | 2012 | Review article | Unbiased and ethical professionals,  Skilled critics,  Respectful communicators | 16, 42, 46, 58 | 4 |
| Cervera, M. L. G.(9) | Colombia | Spanish | Salud Uninorte | 2008 | Review article | Proficient experts in their field,  Unbiased and ethical professionals,  Self-critical professionals,  Reliable professionals,  Skilled critics,  Respectful communicators,  Advisors | 1, 13, 14, 15, 16, 33, 35, 38, 42, 58, 70 | 11 |
| Cho, Y.G.(10) | Korea | English | Korean Journal of Family Medicine | 2013 | Review article | Unbiased and ethical professionals,  Self-critical professionals, Reliable professionals | 13, 14, 18, 31, 36 | 5 |
| Dhammi, I. K.(11) | India | English | Indian Journal of Orthopaedics | 2013 | Review article | Unbiased and ethical professionals,  Self-critical professionals,  Reliable professionals | 13, 31, 36 | 3 |
| Doi, S. A. R.(12) | Australia | English | Accountability in Research-Policies and Quality Assurance | 2016 | Review article | N/A | N/A | 0 |
| Einarson, T. R.(13) | Canada | English | Journal of Population Therapeutics and Clinical Pharmacology | 2012 | Review article | Dutiful/altruistic towards scientific community,  Unbiased and ethical professionals,  Self-critical professionals,  Reliable professionals,  Skilled critics,  Respectful communicators,  Advisors | 4, 6, 15, 16, 30, 32, 35, 42, 43, 44, 45, 46, 58, 70 | 14 |
| Felton, G.(14) | USA | English | Journal of Professional Nursing | 1995 | Review article | Gatekeepers | 63 | 1 |
| Gasparyan, A. Y. (15) | UK | English | Rheumatology International | 2011 | Review article | Reliable professionals | 35, 38 | 2 |
| Greenwood, D. C.(16) | UK | English | BMC Medicine | 2015 | Review article | Self-critical professionals,  Skilled critics,  Advisors | 32, 42, 48, 70 | 4 |
| Happell, B.(17) | Australia | English | International Journal of Psychiatric Nursing Research | 2008 | Review article | Familiar with journal,  Self-critical professionals,  Unbiased and ethical professionals,  Reliable professionals,  Skilled critics,  Respectful communicators,  Educators | 11, 14, 23, 32, 34, 36, 40, 42, 43, 46, 48, 58, 59, 66 | 14 |
| Hill, J.(18) | USA | English | Journal of Electrocardiology | 2016 | Review article | Proficient experts in their field,  Dutiful/altruistic towards scientific community,  Unbiased and ethical professionals,  Reliable professionals,  Skilled critics,  Respectful communicators, Gatekeepers,  Advocates,  Advisors,  Reviewers should not | 1, 2, 4, 14, 15, 35, 42, 45, 50, 51, 58, 63, 67, 68, 71, 72, 73 | 17 |
| Hojat, M.(19) | USA | English | Advances in Health Sciences Education | 2003 | Review article | Unbiased and ethical professionals,  Self-critical professionals,  Reliable professionals,  Skilled critics,  Respectful communicators,  Educators,  Advocates | 13, 32, 36,  42, 43, 47, 57, 59, 65, 68 | 10 |
| Ingelfinger, F. J.(20) | USA | English | New England Journal of Medicine | 1974 | Review article | Unbiased and ethical professionals,  Unbiased and ethical professionals | 14, 20 | 2 |
| Jasper, M.(21) | UK | English | Nursing Inquiry | 2014 | Review article | Proficient experts in their field,  Familiar with journal,  Unbiased and ethical professionals,  Skilled critics,  Respectful communicators,  Gatekeepers,  Advisors | 1, 2, 11, 16, 42, 43, 60, 61, 64, 70 | 10 |
| Knudson, D. V.(22) | USA | English | Research Quarterly for Exercise and Sport | 2014 | Review article | Unbiased and ethical professionals,  Self-critical professionals,  Skilled critics | 15, 33, 42, 43 | 4 |
| Lawrence, D.(23) | Australia | English | Journal of the Canandian Chiropractice Association | 2008 | Review article | Unbiased and ethical professionals,  Advisors,  Reviewers should not | 13, 18, 70, 73 | 4 |
| Lovejoy, T. I.(24) | USA | English | Annals of Behavioral Medicine | 2011 | Review article | Unbiased and ethical professionals,  Self-critical professionals,  Reliable professionals,  Skilled critics,  Respectful communicators,  Advisors | 13, 31, 36, 38, 43, 57, 70 | 7 |
| Lyne, P.(25) | UK | English | Physiotherapy Practice | 1989 | Review article | Proficient experts in their field,  Unbiased and ethical professionals,  Educators | 1, 17, 66 | 3 |
| Matthews, J. B.(26) | USA | English | Journal of Gastrointestinal Surgery | 2014 | Review article | Dutiful/altruistic towards scientific community | 4, 5 | 2 |
| Mayden, K.(27) | USA | English | Journal of the Advanced Practitioner in Oncology | 2012 | Review article | Proficient experts in their field,  Dutiful/altruistic towards scientific community,  Familiar with journal,  Unbiased and ethical professionals,  Reliable professionals,  Skilled critics,  Respectful communicators,  Advocates,  Advisors,  Reviewers should not | 1, 2, 4, 11, 14, 15, 16, 18, 35, 37, 42, 43, 44, 45, 48, 50, 55, 59, 68, 70, 75 | 21 |
| Manchikanti, L.(28) | USA | English | Pain Physician Journal | 2015 | Review article | Unbiased and ethical professionals,  Reliable professionals | 15, 17, 26, 30, 35 | 5 |
| Neale, A. V.(29) | N/A | English | Journal of the American Board of Family Medicine | 2006 | Review article | Familiar with journal,  Unbiased and ethical professionals,  Self-critical professionals,  Reliable professionals, Skilled critics,  Advisors | 11, 13, 14, 16, 20, 21, 22, 31, 33, 35, 43, 52, 71 | 13 |
| Onitilo, A. A.(30) | USA | English | Accountability in Research-Policies and Quality Assurance | 2013 | Review article | Skilled critics,  Advisors | 43, 70, 71 | 3 |
| Piqué Angordans, J.(31) | Spain | English | Index de Enfermeria | 2008 | Review article | Proficient experts in their field,  Self-critical professionals,  Unbiased and ethical professionals,  Reliable professionals,  Skilled critics,  Respectful communicators | 1, 2, 17, 25, 32, 33, 35, 42, 57 | 9 |
| Ribera, J. M.(32) | Spain | English | Medicina Clinica | 2005 | Review article | N/A | N/A | 0 |
| Ruben, R. J.(33) | USA | English | International Journal of Pediatric Otorhinolaryngology | 2003 | Review article | Proficient experts in their field,  Reliable professionals,  Educators | 1, 35, 65 | 3 |
| Schriger, D. L.(34) | USA | English | Annals of Emergency Medicine | 2015 | Review article | Unbiased and ethical professionals,  Skilled critics,  Advisors | 17, 43, 70 | 3 |
| Shashok, K.(35) | Spain | English | Medscape General Medicine | 2005 | Review article | Skilled critics | 43 | 1 |
| Siedlecki, S. L.(36) | USA | English | Nursing | 2015 | Review article | Proficient experts in their field,  Familiar with journal,  Unbiased and ethical professionals,  Self-critical professionals,  Reliable professionals,  Skilled critics,  Respectful communicators,  Reviewers should not | 1, 11, 13, 14, 15, 16, 22, 31, 35, 36, 43, 44, 47, 59, 72 | 15 |
| Siegel, V.(37) | USA | English | Disease Models & Mechanisms | 2008 | Review article | Proficient experts in their field,  Familiar with journal | 1, 11 | 2 |
| Simons-Morton, B.(38) | USA | English | Health Education & Behavior | 2012 | Review article | Self-critical professionals,  Reliable professionals,  Skilled critics,  Respectful communicators,  Reviewers should not | 35, 38, 42, 43, 46, 59, 60, 62, 75 | 9 |
| Simpson, K. J.(39) | USA | English | International Journal of Exercise Science | 2008 | Review article | Dutiful/altruistic towards scientific community,  Familiar with journal,  Self-critical professionals,  Skilled critics,  Gatekeepers,  Advisors,  Reviewers should not | 4, 11, 31, 42, 43, 59, 63, 71, 75 | 9 |
| Skaik, Y.(40) | Palestine | English | Pakistan Journal of Medical Sciences | 2015 | Review article | Proficient experts in their field,  Advisors,  Reviewers should not | 1, 70, 72 | 3 |
| Stahel, P. F.(41) | USA | English | BMC Medicine | 2016 | Review article | Proficient experts in their field,  Dutiful/altruistic towards scientific community,  Unbiased and ethical professionals,  Reliable professionals,  Gatekeepers | 1, 4, 5, 17, 35, 38, 63 | 7 |
| Tandon, R.(42) | USA | English | Asian Journal of Psychiatry | 2014 | Review article | Dutiful/altruistic towards scientific community,  Unbiased and ethical professionals,  Self-critical professionals,  Skilled critics,  Respectful communicators,  Advisors | 4, 13, 21, 32, 43, 46, 57, 71 | 8 |
| Triaridis, S.(43) | Greece | English | Hippokratia | 2010 | Review article | Dutiful/altruistic towards scientific community,  Unbiased and ethical professionals,  Self-critical professionals,  Reliable professionals,  Skilled critics,  Advocates | 4, 13, 14, 19, 21, 31, 32, 36, 42, 45, 47, 68 | 12 |
| Wagner A. K.(44) | USA | English | American Journal of Physical Medicine and Rehabilitation | 2003 | Review article | Proficient experts in their field,  Unbiased and ethical professionals,  Reliable professionals,  Skilled critics,  Reviewers should not | 1, 2, 36, 47, 74 | 5 |
| Weil, J.(45) | N/A | English | Journal of Genetic Counseling | 2004 | Review article | Proficient experts in their field,  Familiar with journal,  Skilled critics | 1, 11, 49 | 3 |
| **Total** |  |  |  |  |  |  |  | **306** |

^B^ Corresponds to item number from the list of roles (Table 1)

^C^ Number of extracted roles statements

**Table 2** Tasks from review articles

| **Author** | **Country** | **Language** | **Journal** | **Year** | **Design** | **Theme(s)** | **Task items^b^** | **# of tasks^c^** |
| --- | --- | --- | --- | --- | --- | --- | --- | --- |
| Alexander, G.(1) | USA | English | Maternal & Child Health Journal | 2005 | Review article | Organization and approach to review,  Make general comments,  Title is accurate,  Abstract,  Introduction, Methods,  Results, Discussion/Conclusion, References,  Assess manuscript presentation,  Provide recommendations | 3, 7, 12, 14, 15, 16, 17, 21, 22, 23, 26, 30, 31, 32, 33, 34, 35, 36, 39, 45, 47, 50, 52, 53, 54, 56, 62, 63, 65, 66, 68, 70, 71 | 33 |
| Alfonso, F.(2) | Spain | Spanish | Archivos de Cardiologia de Mexico | 2010 | Review article | Organization and approach to review,  Make general comments,  Methods, Discussion/Conclusion, Assess manuscript presentation | 1, 2, 11, 12, 14, 30, 50, 54, 62, 63, 64, 71 | 12 |
| Ali, P. A.(3) | UK | English | Nursing Open | 2016 | Review article | N/A | N/A | 0 |
| Barroga, E.(4) | Japan | English | Journal of Korean Medical Science | 2014 | Review article | Make general comments,  Results | 11, 13, 14, 49 | 4 |
| Baxt, W. G.(5) | USA | English | Annals of Emergency Medicine | 1998 | Review article | Make general comments, Introduction, Discussion/Conclusion, Provide recommendations | 11, 12, 26, 50, 72 | 5 |
| Bayne, S. C.(6) | USA | English | The Journal of Prosthetic Dentistry | 2003 | Review article | Provide recommendations | 70 | 1 |
| Bingham, C. L.(7) | Australia | English | THE LANCET | 1998 | Review article | Organization and approach to review,  Make general comments,  Provide recommendations | 3, 13, 17, 70, 72 | 5 |
| Brand, R. A. (8) | USA | English | Clinical Orthopaedics and Related Research | 2012 | Review article | Organization and approach to review, Make general comments, Introduction, Methods,  Results, Discussion/Conclusion, Assess manuscript presentation,  Provide recommendations | 2, 6, 9, 11, 13, 16, 17, 26, 28, 30, 31, 32, 33, 34, 35, 36, 37, 38, 39, 40, 41, 42, 45, 46, 52, 54, 63, 70 | 28 |
| Cervera, M. L. G.(9) | Colombia | Spanish | Salud Uninorte | 2008 | Review article | Organization and approach to review,  Make general comments | 2, 11, 13, 14, 15, 17 | 6 |
| Cho, Y.G.(10) | Korea | English | Korean Journal of Family Medicine | 2013 | Review article | Organization and approach to review, Make general comments,  Title is accurate,  Abstract,  Introduction,  Methods,  Results, Discussion/Conclusion, References,  Assess manuscript presentation,  Provide recommendations | 3, 11, 12, 13, 14, 16, 21, 22, 23, 26, 28, 35, 37, 38, 45, 47, 50, 56, 64, 70, 72 | 21 |
| Dhammi, I. K.(11) | India | English | Indian Journal of Orthopaedics | 2013 | Review article | Organization and approach to review,  Make general comments,  Title is accurate,  Abstract,  Introduction, Methods,  Results, Discussion/Conclusion, References,  Assess manuscript presentation | 1, 11, 12, 13, 17, 20, 21, 22, 23, 26, 27, 31, 32, 33, 34, 36, 37, 38, 39, 40, 41, 43, 44, 45, 47, 50, 51, 52, 53, 56, 58, 63 | 32 |
| Doi, S. A. R.(12) | Australia | English | Accountability in Research-Policies and Quality Assurance | 2016 | Review article | Make general comments, Introduction, Methods,  Results, Discussion/Conclusion | 11, 12, 15, 26, 27, 28, 30, 31, 46, 50 | 10 |
| Einarson, T. R.(13) | Canada | English | Journal of Population Therapeutics and Clinical Pharmacology | 2012 | Review article | Make general comments,  Methods,  Address ethical aspects,  Provide recommendations | 11, 13, 15, 31, 32, 34, 35, 38, 57, 70, 71 | 11 |
| Felton, G.(14) | USA | English | Journal of Professional Nursing | 1995 | Review article | Organization and approach to review, Make general comments, Introduction, Methods,  Assess manuscript presentation,  Provide recommendations | 2, 11, 15, 18, 26, 29, 30, 32, 33, 63, 70, 71 | 12 |
| Gasparyan, A. Y.(15) | UK | English | Rheumatology International | 2011 | Review article | Make general comments, Introduction, Methods,  Results,  References,  Assess manuscript presentation,  Provide recommendations | 13, 26, 29, 30, 45, 56, 67, 70 | 8 |
| Greenwood, D. C.(16) | UK | English | BMC Medicine | 2015 | Review article | Methods,  Results, Discussion/Conclusion, Assess manuscript presentation | 31, 32, 33, 34, 35, 36, 38, 39, 40, 41, 42, 44, 47, 48, 50, 51, 52, 64, 69 | 19 |
| Happell, B.(17) | Australia | English | International Journal of Psychiatric Nursing Research | 2008 | Review article | Make general comments,  Results, Discussion/Conclusion, Assess manuscript presentation,  Provide recommendations | 11, 16, 17, 47, 50, 64, 71 | 7 |
| Hill, J.(18) | USA | English | Journal of Electrocardiology | 2016 | Review article | Abstract,  Introduction, Methods,  Results, Discussion/Conclusion, References,  Provide recommendations | 22, 23, 27, 29, 30, 32, 36, 45, 46, 48, 50, 51, 52, 56, 70 | 15 |
| Hojat, M.(19) | USA | English | Advances in Health Sciences Education | 2003 | Review article | Organization and approach to review | 1 | 1 |
| Ingelfinger, F. J.(20) | USA | English | New England Journal of Medicine | 1974 | Review article | N/A | N/A | 0 |
| Jasper, M.(21) | UK | English | Nursing Inquiry | 2014 | Review article | Organization and approach to review,  Make general comments,  Methods,  Assess manuscript presentation,  Provide recommendations | 1, 11, 12, 14, 15, 17, 18, 30, 62, 64, 71 | 11 |
| Knudson, D. V.(22) | USA | English | Research Quarterly for Exercise and Sport | 2014 | Review article | Make general comments | 11, 16 | 2 |
| Lawrence, D.(23) | Australia | English | Journal of the Canandian Chiropractice Association | 2008 | Review article | Organization and approach to review, Make general comments,  Methods,  Title is accurate, Abstract,  Results, Discussion/Conclusion, Assess manuscript presentation,  Provide recommendations, | 3, 12, 14, 17, 21, 23, 43, 45, 50, 67, 68, 69, 71 | 13 |
| Lovejoy, T. I.(24) | USA | English | Annals of Behavioral Medicine | 2011 | Review article | Organization and approach to review, Make general comments,  Title is accurate, Introduction,  Abstract,  Methods,  Results, Discussion/Conclusion, Assess manuscript presentation,  Provide recommendations | 1, 4, 11, 16, 21, 22, 23, 25, 26, 28, 29, 35, 37, 45, 50, 62, 73 | 17 |
| Lyne, P.(25) | UK | English | Physiotherapy Practice | 1989 | Review article | Title is accurate, Introduction, Methods,  Results, Discussion/Conclusion, Assess manuscript presentation, | 21, 26, 27, 28, 30, 31, 32, 33, 34, 35, 36, 37, 38, 39, 40, 41, 42, 44, 46, 47, 48, 50, 51, 54, 62, 63, 68 | 27 |
| Matthews, J. B.(26) | USA | English | Journal of Gastrointestinal Surgery | 2014 | Review article | Make general comments,  Methods,  Results, Discussion/Conclusion, Assess manuscript presentation,  Provide recommendations | 11, 13, 30, 32, 35, 46, 48, 50, 51, 63, 70, 71 | 12 |
| Mayden, K.(27) | USA | English | Journal of the Advanced Practitioner in Oncology | 2012 | Review article | Abstract,  Introduction, Methods,  Results, Discussion/Conclusion, References,  Address ethical aspects,  Assess manuscript presentation | 22, 23, 26, 27, 30, 31, 32, 33, 34, 35, 36, 37, 38, 39, 40, 41, 42, 44, 45, 46, 47, 50, 51, 52, 53, 56, 57, 62, 63, 66 | 30 |
| Manchikanti, L.(28) | USA | English | Pain Physician Journal | 2015 | Review article | Make general comments,  Methods,  Address ethical aspects,  Assess manuscript presentation,  Provide recommendations | 11, 12, 14, 30, 31, 57, 64, 70 | 8 |
| Neale, A. V.(29) | N/A | English | Journal of the American Board of Family Medicine | 2006 | Review article | Organization and approach to review,  Make general comments,  Title is accurate, Abstract,  Introduction, Methods,  Results, Discussion/Conclusion,  References,  Provide recommendations | 3, 13, 16, 21, 22, 23, 27, 28, 30, 31, 32, 33, 34, 35, 36, 37, 38, 39, 41, 42, 44, 45, 46, 50, 51, 52, 56, 71, 72 | 29 |
| Onitilo, A. A.(30) | USA | English | Accountability in Research-Policies and Quality Assurance | 2013 | Review article | Organization and approach to review, Make general comments,  Methods,  Results, Discussion/Conclusion, References,  Assess manuscript presentation,  Provide recommendations | 5, 11, 12, 15, 17, 18, 20, 43, 45, 46, 51, 53, 56, 64, 66, 70 | 16 |
| Piqué Angordans, J.(31) | Spain | English | Index de Enfermeria | 2008 | Review article | N/A | N/A | 0 |
| Ribera, J. M.(32) | Spain | English | Medicina Clinica | 2005 | Review article | Organization and approach to review,  Make general comments,  Title is accurate,  Abstract,  Introduction, Methods, Discussion/Conclusion, Address ethical aspects,  Assess manuscript presentation,  Provide recommendations | 3, 16, 21, 22, 23, 26, 27, 29, 30, 31, 33, 34, 35, 36, 39, 40, 50, 51, 52, 54, 57, 58, 63, 72 | 24 |
| Ruben, R. J.(33) | USA | English | International Journal of Pediatric Otorhinolaryngology | 2003 | Review article | Organization and approach to review, Methods, Discussion/Conclusion, References | 2, 31, 32, 50, 51, 56 | 6 |
| Schriger, D. L.(34) | USA | English | Annals of Emergency Medicine | 2015 | Review article | N/A | N/A | 0 |
| Shashok, K.(35) | Spain | English | MedGenMed Medscape General Medicine | 2005 | Review article | Organization and approach to review, Methods,  Results, Discussion/Conclusion | 1, 30, 31, 46, 50 | 5 |
| Siedlecki, S. L.(36) | USA | English | Nursing | 2015 | Review article | Organization and approach to review | 9 | 1 |
| Siegel, V.(37) | USA | English | Disease Models & Mechanisms | 2008 | Review article | Organization and approach to review, Make general comments,Title is accurate, Abstract,  Introduction, Methods,  Results, Discussion/Conclusion,  Assess manuscript presentation | 2, 11, 12, 14, 16, 21, 23, 24, 26, 27, 28, 29, 30, 35, 37, 45, 50, 52, 66 | 19 |
| Simons-Morton, B.(38) | USA | English | Health Education & Behavior | 2012 | Review article | Organization and approach to review,  Make general comments,  Abstract,  Introduction, Methods,  Results, Discussion/Conclusion, Address ethical aspects,  Assess manuscript presentation,  Provide recommendations | 2, 13, 15, 16, 18, 22, 23, 26, 27, 28, 29, 30, 31, 32, 33, 34, 37, 39, 45, 46, 47, 50, 51, 52, 53, 54, 57, 62, 63, 66, 67, 70 | 32 |
| Simpson, K. J.(39) | USA | English | International Journal of Exercise Science | 2008 | Review article | Organization and approach to review, Make general comments,  Abstract,  Introduction, Methods,  Results, Discussion/Conclusion, References,  Address ethical aspects,  Assess manuscript presentation,  Provide recommendations | 2, 11, 12, 13, 14, 16, 22, 23, 25, 26, 27, 30, 32, 33, 34, 39, 45, 46, 47, 50, 51, 52, 56, 57, 62, 63, 65, 66, 67, 70, 71 | 31 |
| Skaik, Y.(40) | Palestine | English | Pakistan Journal of Medical Sciences | 2015 | Review article | Make general comments,  Methods, Discussion/Conclusion | 12, 13, 14, 30, 33, 50 | 6 |
| Stahel, P. F.(41) | USA | English | BMC Medicine | 2016 | Review article | Make general comments,  Title is accurate, Abstract,  Introduction, Methods,  Results, Discussion/Conclusion, Provide recommendations | 13, 14, 15, 16, 21, 24, 26, 27, 29, 30, 39, 46, 50, 51, 52, 70 | 16 |
| Tandon, R.(42) | USA | English | Asian Journal of Psychiatry | 2014 | Review article | Organization and approach to review,  Make general comments,  Address ethical aspects,  Assess manuscript presentation,  Provide recommendations | 1, 3, 11, 14, 57, 62, 64, 70, 71 | 9 |
| Triaridis, S.(43) | Greece | English | Hippokratia | 2010 | Review article | Organization and approach to review, Make general comments, Discussion/Conclusion, References,  Address ethical aspects | 2, 12, 14, 50, 56, 57, 58 | 7 |
| Wagner A. K.(44) | USA | English | American Journal of Physical Medicine and Rehabilitation | 2003 | Review article | Make general comments,  Abstract,  Methods, Address ethical aspects,  Assess manuscript presentation,  Provide recommendations | 13, 15, 22, 33, 57, 64, 66, 71 | 8 |
| Weil, J.(45) | N/A | English | Journal of Genetic Counseling | 2004 | Review article | Make general comments, Introduction, Methods, Discussion/Conclusion, Assess manuscript presentation, Provide recommendations, | 16, 28, 31, 32, 50, 66, 70, | 8 |
| **Total** |  |  |  |  |  |  |  | **567** |

^B^ Corresponds to item number from the list of task (Table 2)

^C^ Number of extracted task statements

## References

1.	Alexander GR. A guide to reviewing manuscripts. Matern Child Health J. 2005 Mar;9(1):113–7.

2.	Alfonso F. A critical review of the “peer review” process. Arch Cardiol Mex. 2010;80(4):272–82.

3.	Ali PA, Watson R. Peer review and the publication process. Nurs Open. 2016 Oct 1;3(4):193–202.

4.	Barroga EF. Safeguarding the Integrity of Science Communication by Restraining “Rational Cheating” in Peer Review. J Korean Med Sci. 2014 Nov;29(11):1450–2.

5.	Baxt WG, Waeckerle JF, Berlin JA. Who reviews the reviewers? Feasibility of using a fictitious manuscript to evaluate peer reviewer performance. Ann Emerg Med [Internet]. 1998;32. Available from: http://dx.doi.org/10.1016/S0196-0644(98)70006-X

6.	Bayne SC, McGivney GP, Mazer SC. Scientific composition and review of manuscripts for publication in peer-reviewed dental journals. J Prosthet Dent. 2003 Feb;89(2):201–18.

7.	Bingham C. Peer review on the Internet: a better class of conversation. Lancet. 1998 Mar;351:10–4.

8.	Brand RA. Reviewing for clinical orthopaedics and related research. Clin Orthop. 2012 Sep;470(9):2622–5.

9.	Cervera MLG, Hincapié J, Jackman J, Herrera O, Uribe CVC. Peer review: What it’s and what it’s for? Salud Uninorte. 2008;24(2):258–72.

10.	Cho YG, Park HA. Peer Review Process in Medical Journals. Korean J Fam Med. 2013 Nov;34(6):372–6.

11.	Dhammi IK, Kumar S. Process of peer review continues. Indian J Orthop. 2013 Nov;47(6):537–9.

12.	Doi SAR, Salzman-Scott SA, Onitilo AA. Validation of the CoRE Questionnaire for a Medical Journal Peer Review. Account Res-Policies Qual Assur. 2016 Jan;23(1):47–52.

13.	Einarson TR, Koren G. To accept or reject? A guide to peer reviewing of medical journal papers. J Popul Ther Clin Pharmacol. 2012;19(2):e328–33.

14.	Felton G, Swanson EA. Peer-Review. J Prof Nurs. 1995 Jan;11(1):16–23.

15.	Gasparyan AY, Ayvazyan L, Blackmore H, Kitas GD. Writing a narrative biomedical review: Considerations for authors, peer reviewers, and editors. Rheumatol Int. 2011;31(11):1409–17.

16.	Greenwood DC, Freeman JV. How to spot a statistical problem: advice for a non-statistical reviewer. BMC Med. 2015 Nov 2;13:1–3.

17.	Happell B. The responsibility of review: guidelines to promote professional courtesy and commitment through the peer review process. Int J Psychiatr Nurs Res. 2008;13(3):1–9.

18.	Hill JA. How to Review a Manuscript. J Electrocardiol. 2016 Apr 3;49(2):109–11.

19.	Hojat M, Gonnella JS, Caelleigh AS. Impartial Judgment by the “Gatekeepers” of Science: Fallibility and Accountability in the Peer Review Process [Internet]. 2003. Available from: http://ovidsp.ovid.com/ovidweb.cgi?T=JS&PAGE=reference&D=psyc4&NEWS=N&AN=2003-02402-002

20.	Ingelfinger FJ. Peer review in biomedical publication. Am J Med. 1974;56(5):686–92.

21.	Jasper M, Vaismoradi M, Bondas T, Turunen H. Validity and reliability of the scientific review process in nursing journals - time for a rethink? Nurs Inq. 2014 Jun;21(2):92–100.

22.	Knudson DV, Morrow JR, Thomas JR. Advancing Kinesiology Through Improved Peer Review. Res Q Exerc Sport. 2014 Jun;85(2):127–35.

23.	Lawrence D, Ebrall P. The scholarship of critical review: improving quality and relevance. J Can Chiropr Assoc. 2008 Dec;52(4):211–23.

24.	Lovejoy TI, Revenson TA, France CR. Reviewing Manuscripts for Peer-Review Journals: A Primer for Novice and Seasoned Reviewers. Ann Behav Med. 2011 Aug;42(1):1–13.

25.	Lyne P. Writing for publication. Peer review of papers submitted for publication. Physiother Theory Pract. 1989;5(1):17–23.

26.	Matthews JB. 2013 SSAT Presidential Address: Peer Review. J Gastrointest Surg. 2014 Jan;18(1):1–6.

27.	Mayden KD. Peer review: publication’s gold standard. J Adv Pract Oncol. 2012 Mar;3(2):117–22.

28.	Manchikanti L, Kaye AD, Boswell M, Hirsch JA. Medical journal peer review: Process and bias. Pain Physician. 2015;18(1):E1–14.

29.	Neale AV, Schwartz KL, Bowman MA. Peer reviewing for the Journal of the American Board of Family Medicine: What does it take? J Am Board Fam Med. 2006 Nov;19(6):643–7.

30.	Onitilo AA, Engel JM, Salzman-Scott SA, Stankowski RV, Doi SAR. Reliability of Reviewer Ratings in the Manuscript Peer Review Process: An Opportunity for Improvement. Account Res-Policies Qual Assur. 2013 Jul;20(4):270–84.

31.	Piqué Angordans J, Camaño Puig R. Nursing research and peer review. Index Enfermeria [Internet]. 2008;17(3). Available from: https://www.scopus.com/inward/record.uri?eid=2-s2.0-66249133965&partnerID=40&md5=9ef41b7a52f33f7884ea323bb315ca82

32.	Ribera JM, Cardellach F, Selva A. The processes of manuscript evaluation and publication in Medicina Clinica. [Spanish] Procesos de revision y de edicion en Medicina Clinica. Med Clin (Barc). 2005 Dec;125(SUPPL. 1):3–7.

33.	Ruben RJ. The promotion of academic pediatric otolaryngology by journal peer review. Int J Pediatr Otorhinolaryngol. 2003 Dec;67:S165–9.

34.	Schriger DL, Kadera SP, von Elm E. Are Reviewers’ Scores Influenced by Citations to Their Own Work? An Analysis of Submitted Manuscripts and Peer Reviewer Reports. Ann Emerg Med. 2016 Mar;67(3):401-406.e6.

35.	Shashok K. Standardization vs Diversity: How Can We Push Peer Review Research Forward? Medscape Gen Med. 2005 Feb 16;7(1):11.

36.	Siedlecki SL. The ethics of peer review: What to know before saying “yes”. Nursing (Lond). 2015 Dec;45(12):51–4.

37.	Siegel V. The promise of peer review. Dis Model Mech. 2008 Sep;1(2–3):73–7.

38.	Simons-Morton B, Abraido-Lanza AF, Bernhardt JM, Schoenthaler A, Schnitzer A, Allegrante JP. Demystifying peer review [Internet]. 2012 [cited 2002 Jan 11]. Available from: http://ovidsp.ovid.com/ovidweb.cgi?T=JS&PAGE=reference&D=psyc9&NEWS=N&AN=2012-03231-001

39.	Simpson KJ. Reviewing an Original Research Manuscript for the International Journal of Exercise Science: A Guide for Students and Professionals. Int J Exerc Sci. 2008;1(2):43–9.

40.	Skaik Y. The panacea statistical toolbox of a biomedical peer reviewer. Pak J Med Sci. 2015;31(4):999–1001.

41.	Stahel PF, Moore EE. How to review a surgical paper: a guide for junior referees. BMC Med. 2016 Feb 14;14:1–6.

42.	Tandon R. How to review a scientific paper. Asian J Psychiatry. 2014 Oct;11:124–7.

43.	Triaridis S, Kyrgidis A. Peer review and journal impact factor: the two pillars of contemporary medical publishing. Hippokratia. 2010;14(1):5–12.

44.	Wagner AK, Boninger ML, Levy C, Chan L, Gater D, Kirby RL. Peer review: issues in physical medicine and rehabilitation. Am J Phys Med Rehabil. 2003 Oct;82(10):790–802.

45.	Weil J. Peer review: An essential step in the publishing process. J Genet Couns. 2004;13(3):183–7.
